# Developing oxidative stress-responsive neuroprotective nanotheranostic agents for the image-guided treatment of radiotherapy-induced brain injury

**DOI:** 10.7150/ntno.138059

**Published:** 2026-09-24

**Authors:** Saikat Maiti, Maya Teitz, Esteban Velarde, Barbara J. Smith, Xiaoju Yang, Shana Lee, Kristen Lecksell, Anupama Kumari, Troy Kavanaugh III, Timothy Parish, Adnan Bibic, Ethel J. Ngen

**Affiliations:** 1 Russell H. Morgan Department of Radiology and Radiological Science, Johns Hopkins University School of Medicine, Baltimore, MD 21287, USA.; 2 Department of Radiation Oncology and Molecular Radiation Sciences, Johns Hopkins University School of Medicine, Baltimore, MD 21231, USA.; 3 Department of Cell Biology Imaging Facility, Johns Hopkins University School of Medicine, Baltimore, MD 21205, USA.; 4 Department of Molecular and Comparative Pathobiology, Johns Hopkins University School of Medicine, Baltimore, MD 21205, USA.; 5 Department of Pathology, Johns Hopkins University School of Medicine, Baltimore, MD 21287, USA.; 6 F.M. Kirby Research Center for Functional Brain Imaging, Kennedy Krieger Institute, Baltimore, MD 21205, USA.; 7 Johns Hopkins Sidney Kimmel Comprehensive Cancer Center, Baltimore, MD 21287, USA.

**Keywords:** radiation injury mitigation, brain injury mitigation, neuroinflammation mitigation, stimuli-responsive neuroprotective nanotheranostic agents, image-guided neurotherapy

## Abstract

**Rationale:**

Radiotherapy-induced brain injury (RIBI) is a chronic side effect, that affects up to ~90% of brain tumor survivors treated with radiotherapy. Chronic oxidative stress and neuroinflammation are key drivers of RIBI. Here, we developed oxidative stress-responsive polymeric nanotheranostic agents and evaluated their ability to reduce neuroinflammation in a preclinical mouse model of RIBI.

**Method:**

Two oxidative stress-responsive amphiphilic block copolymers possessing varied numbers of phenylboronic acid pinacol ester (BAPE) moieties to scavenge reactive oxygen species (ROS) were designed, synthesized, and characterized by proton and carbon-13 nuclear magnetic resonance spectroscopy. Polymer P2b was designed to have twice as many BAPE moieties as polymer P2a, for a structure-activity relationship study. The polymers were then formulated into nanoparticles and characterized using fluorescence spectroscopy, dynamic light-scattering, transmission and scanning electron microscopy, and fluorescence imaging. The ability of the agents to prevent the degradation of fluorescent R-phycoerythrin (RPE) protein under oxidative stress was also evaluated. Then, the cellular uptake and toxicity of the agents were evaluated in human umbilical vein endothelial cells (HUVECs). Next, the *in vivo* therapeutic efficacies of the agents were evaluated in four groups of eight-week-old female BALB/c mice. Mice from one group (Group 1) were not irradiated and were used as controls, while the right brain hemispheres of mice from the other three groups were stereotactically irradiated at 80 Gy (1.7 Gy/minute). At two weeks post-irradiation, mice were intravenously treated with either a fluorescently labelled nanoparticle or phosphate buffered saline (PBS), as follows: Group 2 (PBS); Group 3 (P2a); Group 4 (P2b); and the delivery of the nanoparticles to the irradiated brain lesion was monitored with fluorescence imaging. Next, all the mice were monitored with multi-parametric magnetic resonance imaging (mp-MRI) and immunohistochemistry (CD68, IBA1, and GFAP) at 1.5 months after nanoparticle administration.

**Results:**

Both polymers readily formed spherical nanoparticular micelles in aqueous milieu at low concentrations (~ 0.08 mg/mL), with comparable hydrodynamic diameters and zeta-potentials of 166 ± 51 nm and **-**2.52 ± 0.658 mV for P2a; and 183 ± 54 nm and **-**2.89 ± 1.58 mV for P2b. The activation kinetics of both nanoparticles was higher under oxidative stress than under normal physiological conditions. Additionally, both nanoparticles prevented the degradation of RPE protein under oxidative stress. Furthermore, both nanoparticles were taken up by HUVECs and did not cause any toxicity. *In vivo*, both nanoparticles were detected in the irradiated brain lesion at 4 h after intravenous administration and retained for at least seven days post-administration. Anatomical T_2_-weighted MRI at 1.5 months after nanoparticle administration showed significantly less edema in both P2a-treated mice (1.46 ± 0.24, *P* = 0.0071, n = 3) and P2b-treated mice (1.48 ± 0.39, *P* = 0.0130, n = 3), than in PBS-treated mice (2.90 ± 0.43). However, contrast-enhanced T_1_W MRI showed significantly less blood-brain barrier permeability in P2b-treated mice (10.72 ± 1.75) than in P2a-treated mice (15.74 ± 1.46, *P* = 0.0187, n = 3) and PBS-treated mice (14.56 ± 0.28, *P* = 0.0198, n = 3). Additionally, immunohistochemistry showed significantly less microglial activation (IBA1) in P2b-treated mice (1.98 ± 0.27) than in P2a-treated mice (3.88 ± 0.22, *P* = 0.0007, n = 3) and PBS-treated mice (6.25 ± 0.51,* P* = 0.0002, n = 3). Less infiltrative peripheral macrophages (CD68) were also detected in P2b-treated mice (4.36 ± 1.83) than in P2a-treated mice (20.79 ± 2.05, *P* = 0.00005, n = 3) and PBS-treated mice (22.95 ± 2.35, *P* = 0.00004, n = 3). Furthermore, significantly less astrogliosis (GFAP) was detected in P2b-treated mice (3.48 ± 0.36) than in P2a-treated mice (5.53 ± 0.92,* P* = 0.0019, n = 3) and PBS-treated mice (23.14 ± 4.66,* P* = 0.0030, n = 3).

**Conclusion:**

Collectively, these results showed that although both nanotheranostic agent P2a and P2b significantly reduced radiation-induced neuroinflammation, nanotheranostic agent P2b (with twice as many ROS scavengers) was more effective at reducing radiation-induced neuroinflammation in the preclinical mouse model of RIBI.

## Introduction

Radiotherapy-induced brain injury (RIBI) is a chronic and debilitating side effect that affects up to ~90% of brain tumor survivors treated with radiotherapy.^1^-^3^ Given the greater vulnerability of developing brains to ionizing radiation, survivors of childhood brain tumors, who were treated with radiotherapy at a young age, are more susceptible to developing RIBI after cancer treatment.^4^-^7^ Thus, with more pediatric brain tumor patients surviving childhood cancers and living into adulthood (thanks to advances in pediatric brain tumor diagnostic and therapeutic strategies), there has been an increase in the incidence of RIBI.^4^-^8^

Mechanistically, conventional radiotherapy generates reactive oxygen species (ROS) in aqueous biological milieu, which destroy cancer cells. However, these ROS also cause oxidative stress in healthy tissue in the tumor vicinity. High ROS concentrations in healthy tissue for prolonged periods of time cause chronic oxidative stress, which leads to neuroinflammation.^9^, ^10^ Chronic oxidative stress and neuroinflammation have been shown to play critical roles in the progression of RIBI and RIBI-associated cognitive impairment.^1^-^3^ More specifically, endothelial cells are particularly vulnerable to ROS generated during conventional radiotherapy, and are damaged by high ROS concentrations.^11^, ^12^ The damaged endothelial cells in the brain then release cytokines, which activate resident microglial cells and astrocytes into a pro-inflammatory state.^11^, ^12^ The damaged endothelial cells also cause the disruption of the blood-brain barrier (BBB), which further enhances neuroinflammation by enabling the infiltration of peripheral macrophages.^11^, ^12^ Additionally, conventional radiotherapy-induced oxidative stress directly activates resident brain microglial cells and astrocytes into a pro-inflammatory state.^1^, ^3^ In this activated pro-inflammatory state, microglial cells and astrocytes also secrete cytokines which further exacerbate neuroinflammation by recruiting more infiltrative peripheral macrophages.^1^, ^3^ Prolonged activation of these microglial cells and astrocytes causes cerebrovascular damage, which leads to ischemia, glial and neuronal cell death, neurodegeneration, and cognitive impairment.^1^, ^3^

To reduce chronic oxidative stress, neuroinflammation, and cognitive impairment associated with conventional radiotherapy, more advanced radiotherapeutic techniques are currently being developed and explored.^13^, ^14^ These include radiotherapeutic techniques such as ultra-high dose rate (FLASH) radiotherapy.^15^-^18^ However, despite the promise of FLASH radiotherapy, it is still at the developmental stages, and several concerns still need to be addressed before a path can be paved for its clinical translation.^19^ Several therapeutic strategies to mitigate RIBI-associated neuroinflammation and cognitive impairment are also being developed and evaluated.^20^, ^21^ These therapeutic strategies include the use of transplanted stem cells.^22^-^25^ However, despite the therapeutic efficacy of transplanted stem cells to reduce RIBI-associated neuroinflammation and cognitive impairment, several limitations are associated with the use of stem cells. These limitations include: 1) The need for invasive intracranial transplantation procedures to get sufficient concentrations of the therapeutic stem cells to the injured site of the brain.^26^, ^27^ 2) The need for immunosuppressant to enhance stem cell engraftment in immune-competent subjects.^27^, ^28^ 3) The risk of teratoma formation after stem cell transplantation.^29^, ^30^

Recently, stem cells have been shown to elicit therapeutic effects via paracrine signaling, in addition to through cell replacement. 31-33 Thus, other therapeutic strategies that utilize stem cell-derived products such as stem cell secretomes and extracellular vesicles are currently being explored.^34^-^36^ However, there are several challenges associated with working with stem cell-derived products such as stem cell-derived extracellular vesicles. These challenges include difficulties in the consistent large-scale production of extracellular vesicles, and difficulties in the characterization of extracellular vesicle batches to minimize inter-batch variability and molecular heterogeneity.^37^ Biocompatible and biodegradable polymeric nanoparticles can be used to overcome some of the challenges associated with the use of stem cell-derived extracellular vesicles. For instance, biodegradable and biocompatible polymeric nanoparticles can be consistently produced on a large scale and effectively characterized with minimum inter-batch variability. Additionally, polymeric nanoparticles can be designed and used for the delivery of a wide range of therapeutic payloads.

In this study, we hypothesized that biocompatible and biodegradable polymeric nanoparticles possessing ROS scavengers and delivered shortly after conventional radiotherapy could be used to alleviate radiotherapy-induced chronic oxidative stress and neuroinflammation in irradiated healthy brain tissues and ultimately improve RIBI outcomes. More specifically, we hypothesized that the sustained release of the ROS scavengers from the oxidative stress-responsive nanoparticles would ensure that high concentrations of ROS scavengers are present in the irradiated brain lesions for long periods of time and ultimately enable the sustained mitigation of chronic oxidative stress and neuroinflammation in irradiated healthy brain tissues and ultimately improve RIBI outcomes.

To test this hypothesis, we designed, synthesized, characterized, and evaluated two oxidative stress-responsive polymeric nanoparticle platforms, possessing varied numbers of phenylboronic acid pinacol ester (BAPE) moieties for ROS scavenging (**Figure 1**).^38^ We hypothesized that these BAPE moieties in the nanoparticles would sustainably scavenge ROS and mitigate chronic oxidative stress and neuroinflammation in irradiated healthy brain tissues. To evaluate the effects of the number of BAPE moieties in the nanoparticles to reduce oxidative stress, a structure-activity study was conducted. Briefly, the block copolymer P2b platform was designed to have twice as many BAPE moieties as the block copolymer P2a platform, (53 BAPE units in P2b versus 26 BAPE units in P2a (**Figure 1**)).^38^ Both amphiphilic block copolymers (P2a and P2b) were designed to also possess similar numbers of pH-responsive tertiary amine group (67 and 63 units for P2a and P2b, respectively), to stabilize the hydrophobic domain of the respective amphiphilic block copolymer-derived nanoparticles at physiological conditions (pH 7.4) and also to modulate their sustainable mitigation of the oxidative stress.^39^, ^40^ Additionally, both amphiphilic block copolymers (P2a & P2b) were designed to possess a fixed number polyethylene glycol (PEG) units (15 units), for improved circulation in the blood stream. The efficacy of the respective polymeric nanoparticles was then evaluated in our previously developed preclinical mouse model of RIBI, using our previously developed imaging biomarkers of RIBI.^41^, ^42^

The central novelty of this study is two-fold: 1) Currently, there are no drugs approved by the United States Food and Drug Administration (US FDA) for the treatment of RIBI and RIBI-associated cognitive impairment.^43^-^46^ Given the clinical symptomatic similarities between Alzheimer's disease and RIBI-associated cognitive impairment, some drugs approved by the US FDA for the management of clinical symptoms of Alzheimer's disease (such as memantine, an N-methyl-D-aspartate type glutamate receptor antagonist), are currently being repurposed and evaluated for the management of clinical symptoms of RIBI-associated cognitive impairment.^43^-^46^ However, most of these agents are low molecular weight agents (such as memantine), which are rapidly cleared from the system shortly after administration and generally require high drug doses daily and high administration frequencies daily (10 mg, twice daily for memantine) to achieve modest improvements in patients with memory problems.^45^, ^47^ Oxidative stress-responsive nanotheranostic agents capable of specifically accumulating in irradiated brain lesions, and being specifically retained in the irradiated brain lesions for long periods of time (even after the administration of low nanotheranostic agent doses), could sustainably release ROS scavengers in the irradiated brain lesions and sustainably mitigate chronic oxidative stress and neuroinflammation in the irradiated tissues over time. This could provide a means of sustainably treating RIBI using low therapeutic agent doses and eliminate the need for frequent drug administration in patients with memory problems. Ultimately, this could improve the adherence of patients with memory problems to treatment regiments/schedules and ultimately improve treatment outcomes. Additionally, this therapeutic approach could also be used for the treatment of other types of radiation injuries and neurodegenerative disorders that are also driven by chronic oxidative stress and neuroinflammation. 2) Another central novelty of this study is the use of noninvasive and clinically translatable image-guided approaches to guide the therapeutic intervention and improve treatment outcomes for RIBI. Prior research in the field has mostly relied on solely using behavioral tests (which generally require large sample sizes to compensate for large variabilities that are generally observed in animal behavior), and invasive immunohistochemical methods to evaluate the efficacy of potential RIBI therapeutic agents.^48^, ^49^ However, here, we use clinically translatable and noninvasive multi-parametric MRI and demonstrate how MRI biomarkers of RIBI can be used to guide therapeutic interventions and improve treatment outcomes for RIBI. We also demonstrate how these clinically translatable and noninvasive MRI biomarkers of RIBI can be used to noninvasively monitor the efficacy of potential therapeutic agents. Ultimately, these MRI biomarkers could provide clinically translatable and noninvasive imaging endpoint readouts of the therapeutic efficacies of the agents, which could facilitate their clinical translation. This image-guided approach can also be extended to the treatment of other types of radiation-induced injuries and neurodegenerative diseases.

## Materials and Methods

### Materials and instruments

All reagents and chemicals were obtained from either Sigma-Aldrich, Inc. (St. Louis, MO) or Thermo Fisher Scientific (Waltham, MA) and used as obtained. 4-(4,4,5,5-Tetramethyl-1,3,2-dioxaborolan-2-yl)benzyl alcohol was procured from Tokyo Chemical Industry (Tokyo, Japan), and 3,6,9,12-tetraoxatetradecane-1,14-diamine was purchased from Ambeed Inc. (Buffalo Grove, IL). The MTS (3-(4,5-dimethylthiazol-2-yl)-5-(3-carboxymethoxyphenyl)-2-(4-sulfophenyl)-2H-tetrazolium) reagent assay kit was purchased from Abcam Inc. (Waltham, MA). All antibodies were obtained from Abcam Inc. (Waltham, MA). Human umbilical vein endothelial cells were purchased from Lonza Bioscience (Rockville, MD). All reactions were monitored with thin layer chromatography using 250 µm silica gel plates with a fluorescent indicator at 254 nm from Whatman (Kent, UK). All column chromatography was done using 40-63 µm silica gel from Sorbent Technologies. Nuclear magnetic resonance (NMR) solvents containing tetramethylsilane (TMS), as an internal standard of reference, were used. The fluorophores IRDye^®^ 680RD and IRDye^®^ 800CW were obtained from LI-COR Biosciences (Lincoln, NE).

### Instruments

NMR spectra were acquired at 25 ºC using a JEOL JNM-ECZL500R spectrometer (Peabody, MA). All absorption and fluorescence spectra were recorded using a Molecular Devices SpectraMax M5 microplate reader (San Jose, CA). All transmission electron microscopy images were captured using a Hitachi 7600TEM (Schaumburg, IL). All scanning electron microscopy images were captured using a ThermoFisher Helios FIB-SEM (Waltham, MA). All dynamic light scattering experiments were conducted using a Malvern Instruments Zetasizer Nano-ZS ZEN3600 particle analyzer (Westborough, MA). All fluorescence imaging was performed on a LI-COR Pearl® Trilogy small animal imaging system (Lincoln, NE). All flow cytometry was performed using a BD Accuri™ C6 flow cytometer (Franklin Lakes, NJ). All irradiation experiments were carried out using a small animal radiation research platform (SARRP) from Xstrahl Inc. (Suwanee, GA). Complete blood counts were conducted using an IDEXX Procyte Dx™ automated clinical hematology analyzer (Westbrook, Maine).

### Synthesis of 4-(4,4,5,5-tetramethyl-1,3,2-dioxaborolan-2-yl)benzyl methacrylate (BM1)

2.5g (0.011 mol) of 4-(hydroxymethyl)phenylboronic acid pinacol ester was dissolved in anhydrous dichloromethane (60 mL) under nitrogen, in a double necked flask. Triethylamine (1.94 mL, 0.016 mol) was next added to the mixture with continuous stirring. The solution was then placed in an ice bath and 1.3 mL (0.013 mol) of methacryloyl chloride in anhydrous dichloromethane (10 mL) was added dropwise in to the solution for 1 h (**Scheme S1**).^40^ The reaction mixture was then stirred overnight at room temperature. After completion of the reaction (determined by thin layer chromatography (TLC)), the reaction mixture was quenched with saturated sodium bicarbonate and extracted with dichloromethane by liquid-liquid extraction. The organic portion of the solution was then thoroughly washed first with water and next with brine. The organic portion was then dried over anhydrous sodium sulphate and filtered. Next, the filtrate was concentrated under reduced pressure and purified by silica gel column chromatography using a hexane: ethyl acetate:: 95:5 eluant. The purified product was concentrated under reduced pressure using a rotary evaporator, and the methacrylate monomer product (BM1) was obtained (**Figure S1 and S2**) as a colorless viscous liquid (85% yield, 2.8 g). Proton (^1^H) NMR (500 MHz, CDCl_3_) δ 7.81 (d, J = 8.0 Hz, 2H), 7.37 (d, J = 8.0 Hz, 2H), 6.16 (s, 1H), 5.60 - 5.56 (m, 1H), 5.20 (s, 2H), 1.98 - 1.94 (m, 3H), 1.34 (s, 12H). Carbon-13 (^13^C) NMR (126 MHz, CDCl_3_) δ 167.30, 139.25, 136.32, 135.13, 129.13, 127.28, 125.99, 83.98, 66.42, 25.00, 18.48.

### Synthesis of poly[poly(ethylene glycol) methyl ether acrylate], P(PEGMEA), (P1)

P(PEGMEA), a macro-chain transfer agent (macro-CTA) was synthesized via the controlled reversible addition-fragmentation chain transfer (RAFT) polymerization technique. Poly(ethylene glycol) methyl ether acrylate with an average molecular weight of 480 g/mol was used in the synthesis. 4-Cyano-4(phenylcarbonothioylthio) pentanoic acid N-succinimidyl ester (CPPA-NHS ester) and azobisisobutyronitrile (AIBN) were used as the chain transfer agent and the initiator respectively, in the synthesis (**Scheme S2**). Briefly, in a 20 mL reaction vial, CPPA-NHS ester (53.5 mg, 0.142 mmol) and poly(ethylene glycol) methyl ether acrylate [PEGMEA] (1.023 g, 2.13 mmol) were dissolved in 3 mL of 1,4-dioxane. AIBN (4.7 mg, 0.028 mmol) was then added to the solution. The reaction vial was then sealed with a septum and left to stir for a few minutes until the initiator was completely solubilized. The reaction mixture was then purged thoroughly with nitrogen gas for 30 minutes and then placed in a pre-heated oil bath at 70 °C for 8 h with continuous stirring. After this, the reaction flask was dipped in a liquid nitrogen bath to quench the polymerization reaction. The reaction mixture was next left to reach room temperature, and then it was diluted with methanol. The polymer solution was next dialyzed for 24 h against methanol, using a dialysis membrane with a 3.5 kDa molecular weight cutoff. The methanol was replaced every 6 h. The solvent was next evaporated using a rotary evaporator, and the polymer was then thoroughly dried under high vacuum for 4 h. A red viscous polymer (P1) was obtained at 86% yield (0.929g), and the composition and number of monomeric units on the polymer were determined by ^1^H NMR spectroscopy (**Figure S3**). ^1^H NMR (500 MHz, CDCl_3_): δ 7.98 (br, 2H, Ph-CTA), 7.56 (br, 1H, Ph-CTA), 7.41 (br, 2H, Ph- CTA), 4.19 (br, 2H, PEGMEA), 3.65 (br, PEGMEA), 3.56 (br, PEGMEA), 3.48 (PEGMEA), 3.39 (3H, -OCH3), 2.86 (br, 4H, CTA-NHS), 2.32, 2.08, 1.68 (br, 7H, CTA; 3H, PEGMEA).

### Synthesis of block copolymers P[PEGMEA-*b*-(DPA-*r*-BM1)] (P2)

The dual functional block copolymers P2a and P2b were synthesized as follows (**Scheme S3 and S4**): First, P(PEGMEA) macro-CTA (0.2g, 0.0267 mmol), 2-(diisopropylamino)ethyl methacrylate (DPA) (0.398g, 1.9 mmol), and monomer BM1 (0.245g, 0.8 mmol for P2a; and 0.49g, 1.6 mmol for P2b) were weighed and placed in the respective reaction flasks, then dissolved in 1,4-dioxane (4 mL). AIBN (1.1mg, 0.007 mmol) was then added to the respective reaction mixtures and the reaction mixtures were stirred at room temperature for 10 minutes under nitrogen. The reaction mixtures were then purged thoroughly with nitrogen gas for 30 minutes, placed in pre-heated oil baths at 70 °C and stirred for 36 h at 70 °C. After this, the reactions were quenched by placing the reaction flasks in liquid nitrogen. The respective products were then purified by dialysis for 24 h, using a membrane with a 12 kDa molecular weight cutoff. Dialysis was done once against methanol for six hours and thrice against dichloromethane for a total of 18 h (six hours each time). The respective products were then concentrated by rotary evaporation, and the polymers were obtained as faint red solids, which were then dried under high vacuum for 4 h. The compositions and number-average molecular weights of the respective polymers were then determined by ^1^H NMR spectroscopy (**Figure S4, S5, and S6**).

P2a yield: 79% (0.671g). P2a^ 1^H NMR (500 MHz, CDCl_3_) δ 7.78 (br, 2H, Ph, BM1), 7.29 (br, 2H, Ph, BM1), 4.92 (br, 2H, -OCH_2_Ph, BM1), 4.17 (br, 2H, PEGMEA), 3.81 (br, 2H, DPA), 3.63, 3.54 (br, 34H, PEGMEA), 3.37 (s, -OCH_3_, 3H, PEGMEA), 2.95 (br, 2H, DPA), 2.59 (br, 2H, DPA), 1.9 (br, 3H, BM1; 3H, DPA), 1.32 (br, 12H, BM1), 0.97 (br, 14H, DPA; 2H, BM1; 3H, PEGMEA).

P2b yield: 77% (0.844g). P2b: ^1^H NMR (500 MHz, CDCl_3_) δ 7.81 (br, 2H, Ph, BM1), 7.31 (br, 2H, Ph, BM1), 4.93 (br, 2H, -OCH_2_Ph, BM1), 4.19 (br, 2H, PEGMEA), 3.82 (br, 2H, DPA), 3.6, 3.56 (br, 34H, PEGMEA), 3.4 (s, -OCH_3_, PEGMEA), 2.96 (br, 2H, DPA), 2.6 (br, 2H, DPA), 1.83 (br, 3H, BM1; 3H, DPA), 1.35 (br, 12H, BM1), 0.99 (br, 14H, DPA; 2H, BM1; 3H, PEGMEA).

### Determining the polymer critical aggregation concentration (CAC)

The CACs of the respective polymers P2a and P2b were determined by measuring the respective polymer encapsulation ability of a hydrophobic dye, Nile Red^®^ (9-(Diethylamino)-5*H*-benzo[*a*]phenoxazin-5-one), using fluorescence spectroscopy. Briefly a series of solutions with varying concentrations of the respective polymers ranging from 0.01 mg/mL to 1.0 mg/mL and a fixed concentration of the Nile Red^®^ dye were used for the measurements. More specifically, 15 μL of a Nile Red^®^ solution in methanol (1.83 mM) was added to a series of vials. The methanol was then removed from the vials by slow evaporation at room temperature. Solutions of the respective polymers prepared in phosphate buffered saline (PBS) at pH 7.4 were then added to the respective vials, and the final polymer concentrations in the respective vials were then adjusted to concentrations ranging from 0.01 mg/mL to 1.0 mg/mL using the required volume of PBS at pH 7.4. The solutions were next sonicated for five minutes and then allowed to stand for four hours. The solutions were then shaken just before the fluorescence spectra were recorded at an excitation wavelength (λ_ex_) of 550 nm and emission wavelength (λ_em_) maxima of 620 nm, using a Molecular Devices SpectraMax M5 microplate reader. The respective CACs were then determined from the extrapolation of the respective inflection points on the plots of maximum Nile Red^®^ fluorescence signal intensity versus polymer concentration.

### Determining the shapes of the polymeric nanoparticles

The internal shapes and structures of the respective polymeric nanoparticles were next determined by transmission electron microscopy (TEM); while the external shapes and surface morphology of the respective polymeric nanoparticles were determined by scanning electron microscopy (SEM). For TEM, the polymer solution (50 µL of 0.1 mg/mL in PBS at pH 7.4) was drop cast on a carbon-coated copper grid (400 mesh, 5-6 nm carbon film, EMS CF400-50-CU) and the excess solution was aspirated. The sample was then dried overnight in a dust-free chamber. The TEM images were next captured on a Hitachi 7600TEM (operating at 80.0 kV and room temperature, using an XR80 high-resolution high-speed (8 Mpixel, 16-bit) AMT CCD camera)). For SEM measurements, the polymer solution (50 µL, 0.1 mg/mLin PBS at pH of 7.4) was placed on a poly-L-lysine coated coverslip, dried overnight in a dust free chamber, then mounted on carbon-coated stubs and coated with 5 nm of gold-palladium (DeskV sputter coater). The SEM images were then acquired using a ThermoFisher Helios FIB-SEM at 5.0 kV.

### Determining the hydrodynamic diameters and zeta potentials of the polymeric nanoparticles

The hydrodynamic size distributions and the zeta potentials of the respective polymeric nanoparticles were determined by dynamic light scattering (DLS) measurements, using a Malvern Nano ZS instrument. Since both polymeric nanoparticles were designed to possess pH and redox responsive moieties for activation under stimuli (oxidative stress), we evaluated the hydrodynamic diameters and zeta potentials of the respective polymeric nanoparticles in both the absence of stimuli (under normoxic conditions at pH 7.4), and in the presence of stimuli (under oxidative stress, demonstrated with 0.5 mM of hydrogen peroxide (H_2_O_2_) at pH of 6.2).

For the DLS measurements in the absence of stimuli, the respective polymeric nanoparticle samples (0.1 mg/mL) were prepared in PBS at pH 7.4 and then filtered through a membrane filter (0.45 µm) to free the solutions from any dust particles. The hydrodynamic diameter and the zeta potential of the respective polymeric nanoparticles were then measured. For the DLS measurements in the presence of stimuli, the respective polymeric nanoparticle samples (0.1 mg/mL) were prepared in PBS at pH 6.2. An H_2_O_2_ solution (1.7 µL of a 1% (w/v) was then added to the respective solutions, and the solutions were then left at room temperature for four hours. The hydrodynamic diameter and the zeta potential of the respective polymeric nanoparticles were then measured. Briefly, the respective samples were placed in a temperature-controlled chamber of the instrument, and the data were acquired. The data were then processed using the instrument's software (Zetasizer Nano software v3.30) to obtain the hydrodynamic diameter and the zeta potentials.

### Determining the stability of the polymeric nanoparticles at pH 7.4

To determine the colloidal stability of the respective polymeric nanoparticles at pH 7.4, a dilution study was carried out. Briefly, nanoparticle solutions (0.1 mg/mL) were prepared in PBS at pH 7.4 and then diluted up to 100 times using PBS at pH 7.4. The solutions were then left at room temperature for two hours, and then the hydrodynamic diameters were measured using a Malvern Instruments Zetasizer Nano-ZS ZEN3600 particle analyzer at room temperature, as described above.

### Determining polymeric nanoparticle activation under stimuli by fluorescence spectroscopy

To determine the kinetics of activation of the polymeric nanoparticles in the presence and absence of stimuli, Nile Red^®^ was first encapsulated in the respective polymeric nanoparticles. The kinetics of the Nile Red^®^ dye's release from the respective activated nanoparticles maintained under different conditions of stimuli activation (different pH values and concentrations of H_2_O_2_) were then measured by quantifying the free Nile Red^®^ dye in solution using fluorescence spectroscopy at the free Nile Red^®^ dye's λ_ex_ of 550 nm and λ_em_ of 620 nm.

More specifically, the Nile Red^®^ dye encapsulation in the polymeric nanoparticles was done as follows: 15 μL of Nile Red^®^ in methanol (1.83 mM) solutions were added to several 5 mL vials. The methanol was then removed from the vials by slow evaporation at room temperature. Solutions of the respective polymers (2.0 mg/mL) in tetrahydrofuran (THF) were then added to the respective vials, and the dried Nile Red^®^ was thoroughly dissolved by slow pipetting and gentle shaking for about five minutes. The respective polymer and the Nile Red^®^ solutions were then added dropwise into 20 mL of a stirring solutions of PBS at pH 7.4, over one hour to prepare nanoparticle solutions of ~ 0.1 mg/mL. The nanoparticle solutions were then left to stir for another two hours at room temperature. The respective solutions were then dialyzed using a membrane with a molecular weight cutoff of 12 kDa, against PBS at pH 7.4 for 24 h. The outer PBS solutions were replaced every 8 h with fresh PBS at pH 7.4, to remove free dye and THF from the solution. The Nile Red^®^ encapsulated polymeric nanoparticle solutions were then collected, and the final concentrations were adjusted to 0.1 mg/mL and kept at 4 °C for the nanoparticle activation and Nile Red^®^ release studies.

For the nanoparticle activation and Nile Red^®^ release studies, the respective Nile Red^®^ encapsulated polymeric nanoparticle solutions (4 mL) were pipetted out into different vials and the pH was either maintained at 7.4 or adjusted to 6.2 using low volumes (< 50 µL) of concentrated hydrochloric acid (1 M). An H_2_O_2_ solution (diluted to 3% w/v in deionized water, from a 30% w/v stock solution) was then added to the respective vials to get solutions with final H_2_O_2_ concentrations of 0.0 mM, 0.1mM, 0.5mM and 1.0 mM. To determine the amount of the encapsulated Nile Red^®^ that was released from the nanoparticles under different conditions of activation at each time point, aliquots of the respective solutions (100 μL) were pipetted into 96- well plate and the fluorescence intensity of free Nile Red^®^ in the respective solutions was measured by fluorescence spectroscopy, using a Molecular Devices SpectraMax M5 microplate reader.

### Determining polymeric nanoparticle activation under stimuli by fluorescence imaging

The polymers were first conjugated to a fluorescent dye (IRDye^®^ 680RD) with a λ_ex_ of 685 nm and a λ_em_ of 720 nm. The respective IRDye^®^ 680RD-conjugated polymers were then used to encapsulate another fluorescent dye (IRDye^®^ 800CW) with a λ_ex_ of 785 nm and a λ_em_ of 820 nm. The IRDye^®^ 680RD-conjugated polymeric nanoparticles encapsulating IRDye^®^ 800CW were then used to image nanoparticle activation and the subsequent release and diffusion of the encapsulated IRDye^®^ 800CW dye. Briefly, whereas the 700 nm fluorescence channel (λ_ex_ = 685 nm and λ_em_ 720 nm) was used to detect nanoparticle activation/degradation; the 800 nm fluorescence channel (λ_ex_ = 785 nm and λ_em_ = 820 nm) was used to detect the release and diffusion of the nanoparticle encapsulated IRDye^®^ 800CW dye.

#### Conjugating the IRDye^®^ 680RD fluorophore to the polymers for phantom fluorescence imaging

The polymers were first conjugated to IRDye^®^ 680RD using N-hydroxysuccinimide (NHS) chemistry as described below (**Scheme S5 and S6**). The respective polymers (1 equivalent) were weighed and placed in respective reaction vials, then dissolved in anhydrous THF to make solutions having polymer concentrations of 2 mg/mL. The solutions were then continuously stirred under an inert nitrogen atmosphere, and triethylamine (25 equivalent) was then added to the respective solutions. Next, 1,14-diamino-3,6,9,12-tetraoxatetradecane (20 equivalent) in 100 µL of THF was added to the respective polymer solutions and the reaction mixtures were stirred for 4 h at room temperature. The respective solutions were then dialyzed thoroughly against THF for six hours to obtain the respective amine-functionalized polymer solutions. Next, IRDye^®^ 680RD NHS ester (1.2 equivalent) was weighed and dissolved in 50 µL of anhydrous THF under an inert nitrogen atmosphere, and the respective amine-functionalized polymer solution were added to the solution. The reaction mixtures were then stirred for 24 h in the dark for complete conjugation, then dialyzed against THF for six hours to remove the unreacted dye. The absorbance of the IRDye^®^ 680RD-conjugated polymers were then measured using a Molecular Devices SpectraMax M5 microplate reader (**Figure S7**).

#### Encapsulating the IRDye^®^ 800CW fluorophore in the polymers for phantom fluorescence imaging

The respective IRDye^®^ 680RD-conjugated polymers (1 equivalent) were then pipetted into vials containing IRDye^®^ 800CW solution (0.5 equivalent) prepared in 25 µL of dimethyl sulfoxide (DMSO). Next, the respective solutions were added dropwise to 20 mL of PBS at pH 7.4, over one hour, to get solutions with final concentrations of 0.1 mg of polymer/mL of PBS. The nanoparticle solutions were then stirred for another one hour at room temperature. The nanoparticle solutions were then dialyzed against PBS at pH 7.4 for 48 h, and the external PBS solutions were replaced every 8 h to remove the unencapsulated fluorophore. The hydrodynamic diameter and the fluorescence signal of the IRDye^®^ 800CW-encapsulated and IRDye^®^ 680RD-conjugated polymeric nanoparticles were then measured using a Malvern Nano ZS instrument and a LI-COR Pearl® Trilogy small animal imaging system respectively (**Figure S8 and S9**).

#### Imaging polymeric nanoparticle activation under stimuli by fluorescence imaging

An agarose gel solution was prepared by dissolving 2% (w/v) of agarose in PBS at pH 7.4 and the solution was then microwaved for one minute. 150 µL of the homogeneous gel was then pipetted into several 200 µL Eppendorf tubes and maintained at room temperature for one hour until the gel solidified. Next, 20 µL of the respective nanoparticle solutions (0.1 µg/µL) were placed on the top of the solidified gels. A series of solutions containing varying concentrations of H_2_O_2_ (0.0 mM, 0.1mM, 0.5mM and 1.0 mM) were prepared at pH 7.4 and pH 6.2. Then, 20 µL of each solution was pipetted on the top of the respective nanoparticles in the agarose gel tubes. The tubes were then closed and the activation of the respective nanoparticles, and the subsequent release of the encapsulated IRDye^®^ 800CW dye under different stimuli conditions (different combinations of pH and H_2_O_2_ concentrations) were imaged with fluorescence imaging at different time points using a LI-COR Pearl® Trilogy small animal imaging system. The fluorescence images were acquired at a resolution of 85 μm, and the 700 nm and 800 nm fluorescence images were then displayed using the LI-COR Pearl® Trilogy small animal imaging software, version 2.0.

The rates of nanoparticle activation, and encapsulated dye release and diffusion through the agarose gel tubes at different stimuli conditions were quantified using the NIH ImageJ software. Briefly, the respective 700 nm and 800 nm fluorescence intensity signals along each tube were measured using the profile analysis function in the NIH ImageJ software. Graphs of the respective 700 nm and 800 nm fluorescence intensities were then plotted against the distance traveled along each tube, at each time point.^50^, ^51^

### Determining the ability of the nanoparticles to protect fluorescent R-phycoerythrin (RPE) from degradation under oxidative stress

The ability of the nanoparticles to protect proteins from degradation under oxidative stress was evaluated using an RPE fluorescent protein assay.^52^ To evaluate the ability of the polymeric nanoparticles (possessing ROS scavenging moieties) to protect the RPE fluorescent protein from degradation under oxidative stress, polyethylene glycol (PEG) with a molecular weight of 7.5 kDa, (and not possessing any ROS scavenging moieties) was used as a negative control in the study.

Briefly, stock solutions of the respective polymers (0.2 mg/mL) and PEG (2 mg/mL) were prepared in 150 mM of phosphate buffer at pH 7.0. A stock solution of the fluorescent RPE protein (1.7 μM) was also prepared in 150 mM of phosphate buffer at pH 7.0. To determine the rate of degradation of the RPE fluorescent protein under normoxic conditions (pH 7.0), a 42.5 nM RPE fluorescent protein solution was prepared by adding 25 μL of the RPE fluorescent protein stock solution to 925 μL of PBS (150 mM) at pH 7.0 to get a solution with a final volume of 950 μL. Next, to test the ability of the respective polymers to protect the RPE fluorescent protein from degradation under oxidative stress (demonstrated by the presence of 10 mM of H_2_O_2_ at pH 7.0), respective RPE fluorescent protein solutions with the desired amount of the respective polymers were prepared by adding 25 μL of the RPE fluorescent protein stock solution to the required volume of the respective polymer stock solutions (0.2 mg/mL), followed by the addition of phosphate buffer (150 mM) at pH 7.0 to final volumes of 950 μL. Aliquots (190 μL) of the respectively prepared solutions (n = 3) were next pipetted into specific wells in a 96-well plate, and the fluorescence signal from solutions were measured at the start of the experiment, using a Molecular Devices SpectraMax M5 microplate reader at a λ_ex_ of 498 nm and a λ_em_ of 575 nm. Then, 10 μL of freshly prepared 200 mM H_2_O_2_ solutions (0.68% w/v) were added to each well, (except the wells containing the control RPE fluorescent protein samples and PBS blank samples, where 10 µL of 150 mM of phosphate buffer was added, instead of H_2_O_2_). Fluorescence measurements were then performed immediately after the addition of H_2_O_2_ and continuously for a total period of 60 minutes at 5-minute intervals. The fluorescence signals obtained from the respective samples were then plotted against time, and the effect of H_2_O_2_ on the loss of the RPE fluorescent signal in the presence and absence of the nanoparticles was determined.

### Determining the cellular uptake and toxicity of the polymeric nanoparticles

Human Umbilical Vein Endothelial Cells (HUVECs) purchased from Lonza Biosciences were used for these experiments. The HUVECs were cultured using the EGM^®^-2 endothelial cell growth medium-2 BulletKit^®^, purchased from Lonza Biosciences. The HUVEC cultures were maintained at 37 °C, 5% CO_2_ and 95% relative humidity, and expanded and passaged according to the Lonza Biosciences HUVEC culture protocol.

For the nanoparticle uptake study, HUVECs were seeded in six-well plates at a seeding density of 1 x10^5^ cells per well. After 24 h, when the cells were 70% - 80% confluent, the respective fluorescently labelled polymeric nanoparticles (5 µg, 10 µg, and 20 µg) were added to the wells to obtain effective nanoparticle concentration of 6.25 µg/µL, 12.5 µg/µL and 25 µg/µL, respectively. The cells were then incubated with the nanoparticles for 24 h at 37 °C. Next, the cells were washed with iced-cold 10 mM PBS. To quench extracellular fluorescence, cells were washed with 500 μL of 0.4% trypan blue in 10 mM of PBS followed by an additional single wash with 10 mM of PBS. The cells were then collected by trypsinization, (using 700 μL of 0.25% trypsin followed by incubation at 37°C for 5 minutes) and centrifugation (at a speed of 5000 rpm for 5 min at 4 °C). The cell pellets were next resuspended in 250 μL of iced-cold 10 mM PBS and kept in ice. The percentage of fluorescently labelled cells were then measured by flow cytometry, using a BD Accuri™ C6 flow cytometer, and recording ten thousand events per analysis. The FlowJo software was then used to analyse and estimate the percentage of fluorescently labelled cells.

For the nanoparticle toxicity study, the cellular viability of the HUVECs in presence of the polymeric nanoparticles was measured using the MTS assay (3-(4,5-dimethylthiazol-2-yl)-5-(3-carboxymethoxyphenyl)-2-(4-sulfophenyl)-2H-tetrazolium).^53^-^56^ Briefly, HUVECs were seeded in 96-well plates at a seeding density of 2 x10^4^ cells per well. After 24 h, the cells were treated with the respective polymeric nanoparticles at 5 µg, 10 µg, 20 µg to obtain effective nanoparticle concentrations of 6.25 µg/µL, 12.5 µg/µL and 25 µg/µL in the respective wells, while keeping the total volume at 200 µL. The viability of the HUVECs was measured after 24 h of incubation with the respective nanoparticles. Briefly, 20 μL of the MTS reagent was added to each well and incubated at 37 °C for 2 h to form soluble formazan crystals. Next, the plate was shaken briefly on a shaker, and the absorbance was measured at 490 nm using a Molecular Devices SpectraMax M5 microplate reader. The percentage viability of the untreated control cells was estimated to be 100%, and relative percentages of viability were calculated by normalizing the absorbance of the nanoparticle-treated wells to the absorbance of the control untreated wells.

### Animals

Female three-to-four-week-old immune competent BALB/cAnNTac (BALB/c) mice were obtained from Taconic Biosciences. The mice were irradiated at eight weeks old, as described below. The mice irradiated at eight weeks old are roughly the equivalent of humans irradiated as middle to late adolescents (15-21-years-old).^57^-^59^ Female mice were used in our current study to expand on our previous finding with this group of mice.^41^ All animal procedures were approved by the Johns Hopkins University Animal Care and Use Committee (JHU ACUC).

### Study design

Four groups of eight-week-old female BALB/c mice were used in this study. Mice from three of the four groups were irradiated at eight weeks old, as previously described.^60^-^64^ Mice from one of the four groups (Group 1 mice) were not irradiated and were used as controls in the study. To determine the nanoparticle dosing strategy, we opted to first evaluate the effectiveness of using a single nanoparticle dose, in this proof-of-concept study. A dose of 20 µg was chosen because the nanoparticles were formulated at a final concentration of (0.1 mg/mL), and since the maximum intravenously injectable volume approved for mice in our JHU ACUC protocol was 200 µL, 200 µL of the respective nanoparticle formulations were administered to the mice and this corresponded to 20 µg of the respective nanoparticles.

To determine the best time point after irradiation to administer the nanotheranostic agents in our RIBI mouse model, we used our previously developed multimodal imaging biomarkers of RIBI (**Figure S10 and S11**).^41^, ^42^ Based on these imaging biomarkers, we determined that two weeks post-irradiation was the best time point to administer the nanotheranostic agents, because at two weeks post-irradiation, although the permeability of the blood-brain barrier (BBB) was detected, no signs of neuroinflammation could be detected at this time point.^41^, ^42^ Maximum neuroinflammation was detected at one month post-irradiation in this RIBI model and coincided with the on-set of mild and transient cognitive impairment (**Figure S10**).^41^, ^42^ Thus, we hypothesized that if the nanotheranostic agents were administered at two weeks post-irradiation, they would specifically accumulate in the irradiated brain lesions (since the BBB was permeable) and prevent or reduce neuroinflammation at later time points.

To determine the best time point at which to evaluate the effectiveness of the nanotheranostic agents to mitigate neuroinflammation, we chose the two months post-irradiation time point, because at this time point, significant neuroinflammation could still be detected in the mice (**Figure S10**).^41^, ^42^ Additionally, maximum astrogliosis was detected in the RIBI model at two months post-irradiation (**Figure S11**).^41^ Astrogliosis is induced by neuroinflammation and is a secondary biomarker of neuroinflammation.^65^ Thus, we hypothesized that if the nanotheranostic agents worked effectively, they would significantly reduce both neuroinflammation and astrogliosis in the preclinical model of RIBI, at two months post-irradiation.

Thus, at two weeks post-irradiation, the irradiated mice were treated intravenously with either a fluorescently labelled polymeric nanoparticle or PBS, as follows: Group 2 (PBS-treated); Group 3 (nanoparticle P2a-treated); Group 4 (nanoparticle P2b-treated). The delivery of the respective agents to the irradiated mouse brain lesions was then monitored by *in vivo* fluorescence imaging, using a LI-COR Pearl® Trilogy small animal imaging system. Next, to evaluate the toxicity of the polymeric nanoparticles, complete blood count tests and comprehensive serum chemistry tests were conducted two weeks after nanoparticle administration (one month post-irradiation). The mice were next monitored with multi-parametric magnetic resonance imaging (mp-MRI) at 1.5 months after nanoparticle administration (two months post-irradiation). After the MRIs, the mice were transcardially perfused, and tissue samples were harvested. Immunohistochemistry was performed on brain tissue samples for signs of neuroinflammation and astrogliosis, while histology was performed on samples of organs such as the livers and kidneys for signs of nanoparticle toxicity.

### Irradiation

The mice were anesthetized with a 2% isoflurane and oxygen mixture, and then irradiated using a small animal radiation research platform (SARRP, Xstrahl Inc., Suwanee, GA) as previously described.^60^-^63^ Briefly, computed tomography (CT) images were first acquired, then a target in the right brain hemisphere (with iso-center at the hippocampus) was chosen on the CT images using the following coordinates: Anterior posterior (AP) = + 3 mm relative to the lambda; medial lateral (ML) = + 0.5 mm relative to the midline; and dorsal ventral (DV) = - 2 mm relative to the skull base. The mice were then irradiated at a dose of 80 Gy and a dose rate of 1.7 Gy per minute, using a single image-guided focal X-ray beam with a 3 mm x 3 mm collimator.

### Synthesis of the fluorescently labelled polymeric nanoparticles for *in vivo* pharmacokinetic and biodistribution analyses

Next, to evaluate the *in vivo* pharmacokinetics and biodistribution of the polymeric nanoparticles, the respective polymers were first conjugated to a fluorescent dye (IRDye^®^ 800CW dye) with a λ_ex_ of 785 nm and a λ_em_ of 820 nm. The respective IRDye^®^ 800CW-conjugated polymers were then used to encapsulate another fluorescent dye (IRDye^®^ 680RD) with a λ_ex_ of 685 nm and a λ_em_ of 720 nm. This was different from what was done for the phantom imaging study where the IRDye^®^ 680RD fluorophore was conjugated to the respective polymers and the IRDye^®^ 800CW was encapsulated in the polymers. For the *in vivo* study, the IRDye^®^ 800CW fluorophore was conjugated to the respective polymers to enable the direct detection of the polymers, since a preliminary *in vivo* fluorescence imaging study showed a high background 720 nm fluorescence signal from the mouse organs. Thus, the IRDye^®^ 680RD was encapsulated in the respective IRDye^®^ 800CW-conjugated polymers, and the feasibility of detecting its release and diffusion from the activated nanoparticle was evaluated as a proxy imaging biomarker of the BAPE ROS scavenging moieties, also released from the activated nanoparticles.

#### Conjugating the IRDye® 800CW fluorophore to the polymers for *in vivo* fluorescence imaging

The polymers were conjugated to IRDye^®^ 800CW NHS ester using NHS chemistry, as described below (**Scheme S7 and S8**). The respective polymers (1 equivalent) were weighed and placed in a reaction vial, then dissolved in anhydrous THF to make solutions having polymer concentrations of 2 mg/mL. The solutions were continuously stirred under an inert nitrogen atmosphere, and then triethylamine (25 equivalent) was added to the respective solutions. Next, 1,14-diamino-3,6,9,12-tetraoxatetradecane (20 equivalent) in 100 µL of THF was added to the respective polymer solutions and the reaction mixtures were stirred for 4 h at room temperature. The solutions were then dialyzed thoroughly against THF for six hours to obtain the respective amine-functionalized polymer solutions. Next, IRDye^®^ 800CW NHS ester (1.2 equivalent) was weighed and dissolved in 50 µL of anhydrous THF under an inert nitrogen atmosphere, and the respective amine-functionalized polymer solutions were added to the solutions. The reaction mixtures were then stirred for 24 h in the dark for complete conjugation. The reaction mixtures were next dialyzed against THF for six hours to remove the unreacted dye. The absorbance of the IRDye^®^ 800CW-conjugated polymers was then measured by absorbance spectroscopy (**Figure S12**).

#### Encapsulating the IRDye^®^ 680RD fluorophore in the polymers for *in vivo* fluorescence imaging

The IRDye^®^ 800CW-conjugated polymers (1 equivalent) were then pipetted into respective vials containing IRDye^®^ 680RD solutions (0.5 equivalent) prepared in 25 µL of DMSO. Next, the solutions were added dropwise to 20 mL of PBS at pH 7.4, over one hour, to get respective solutions of final concentrations of 0.1 mg of polymer/mL of PBS. The nanoparticle solutions were then stirred for another one hour at room temperature. The dye-encapsulated nanoparticle solutions were then dialyzed against PBS at pH 7.4 for 48 h, with the external PBS solution being replaced every 8 h to remove (unencapsulated) fluorophore. The dye-encapsulated nanoparticle solutions were then collected and the concentrations adjusted to 0.1 mg/mL. The solutions were next filtered through a membrane filter (0.45 µm) to free the solutions from any dust particles. The hydrodynamic diameter (**Figure S13**) and the fluorescence (**Figure S14**) of the respective nanoparticle solutions were then measured using a DLS system and a LI-COR Pearl® Trilogy small animal imaging system, respectively, before administration to mice.

### Determining the *in vivo* pharmacokinetics of the polymeric nanoparticles

The IRDye^®^ 800CW-conjugated nanoparticles were administered intravenously to irradiated mice at 2 weeks post-irradiation at a nanoparticle dose of 0.8 mg/kg (200 μL, 0.1 µg/µL). The mice were then imaged by fluorescence imaging at different time points over seven days, to determine the presence of the nanoparticles in the irradiated brain lesion as well as in the other mouse organs, with a LI-COR Pearl® Trilogy small animal imaging system. Irradiated mice, that were intravenously administered PBS at pH 7.4 (instead of the nanoparticles) at 2 weeks post-irradiation, were also imaged and used as controls in the experiment. Additionally, non-irradiated control mice intravenously administered the respective nanoparticles at a dose of 0.8 mg/kg (200 μL, 0.1 µg/ µL) were also imaged and used as controls in the experiment.

All fluorescence images were acquired at a resolution of 85 μm, and the 800 nm fluorescence images were displayed using the LI-COR Pearl® Trilogy small animal imaging software version 2.0. The 800 nm fluorescence channel (λ_ex_ = 785 nm and λ_em_ = 820 nm) was used to detect the pharmacokinetic of the respective nanoparticles.

### Determining the *in vivo* biodistribution of the polymeric nanoparticles

The respective IRDye^®^ 800CW-conjugated and IRDye^®^ 680RD dye-encapsulated nanoparticle solutions (were intravenously administered to irradiated mice at 2 weeks post-irradiation, at a nanoparticle dose of 0.8 mg/kg (200 μL, 0.1 µg/µL). The mice were then sacrificed at different time points after nanoparticle administration (4 h and 24 h), their organs harvested and imaged using fluorescence imaging to determine the presence of the nanoparticles in the brain as well as in the other organs of the mice, with a LI-COR Pearl® Trilogy small animal imaging system. Irradiated mice that were intravenously administered PBS at pH 7.4 (instead of the nanoparticles) at 2 weeks post-irradiation were also sacrificed at different time points after PBS administration (4 h and 24 h), their organs harvested, imaged, and used as controls in the experiment. Additionally, non-irradiated control mice intravenously administered the nanoparticle solutions at a nanoparticle dose of 0.8 mg/kg (200 μL, 0.1 µg/ µL) were also sacrificed at different time points after nanoparticle administration (4 h, and 24 h), their organs harvested, imaged and used as additional controls in the experiment.

All fluorescence images were acquired at a resolution of 85 μm. The 700 nm and 800 nm fluorescence images were then displayed using the LI-COR Pearl® Trilogy small animal imaging software version 2.0. Whereas the 800 nm fluorescence channel was used to detect the biodistribution of the respective nanoparticles; the 700 nm fluorescence channel was used to evaluate the feasibility of detecting the release and biodistribution of the nanoparticle encapsulated IRDye^®^ 680RD. The detection of the IRDye^®^ 680RD released from the activated nanoparticles was also evaluated as a proxy imaging biomarker of the BAPE ROS scavenging moiety, also released from the activated nanoparticles.

### Nanoparticle blood and serum toxicity assays

The respective IRDye^®^ 800CW-conjugated and IRDye^®^ 680RD dye-encapsulated nanoparticle solutions were intravenously administered to irradiated mice at 2 weeks post-irradiation at a nanoparticle dose of 0.8 mg/kg (200 μL, 0.1 µg/µL). Blood samples (200 μL) were then collected from the mice at 2 weeks after intravenous nanoparticle administration via submandibular vein blood draws. Aseptic techniques and single use lancets were used for the blood drawing of each mouse. Briefly, the mice were restrained, and the submandibular vein was punctured with a lancet (only the tip of the lancet entered the tissue at a shallow depth of 1-2 mm). The blood sample was then collected in collection vials. Gentle pressure was then applied with a gauze sponge until bleeding stopped, and the mice were returned to their cages.

For the complete blood count analysis, vials containing the anticoagulant ethylenediaminetetraacetic acid (EDTA) were used for blood collection, and the samples were analyzed within 4 h of sample collection. All complete blood count analysis were performed at the Johns Hopkins University Phenocore, Phenotyping and Experimental Pathology Core, using an IDEXX Procyte Dx™ (Westbrook, Maine) automated clinical hematology analyzer, with up to 18 blood cell parameters.

For the comprehensive serum chemistry tests, blood samples were collected using vials obtained from IDEXX Bioanalytics, and containing a clot activator and a gel barrier to separate- the serum from the blood clot during centrifugation. The blood sample vials were gently inverted after collection, then the samples were allowed to clot for approximately 20 minutes at room temperature. The samples were next centrifuged at 2,000 to 2,500 relative centrifugal force for 10 to 15 minutes. The serum was drawn off and transferred into plain, non-additive, silicone-coated plastic tubes, stored at -20°C and shipped on dry ice to IDEXX Bioanalytics (North Grafton, MA) for the analysis.

### Magnetic resonance imaging (MRI)

All mice were anesthetized with a 2% isoflurane and air mixture, and all MRIs were acquired using an 11.7T Bruker Biospec horizontal bore scanner (Billerica, MA), equipped with a 23 mm Bruker mouse head volume radiofrequency coil. The Paravision 6.1.0 software was used for all image acquisitions.

All *in vivo* contrast-enhanced T_1_-weighted images were acquired before and after the bolus intravenous administration of 200 µL of a 0.25 M (2 mmol/Kg) gadoteridol (ProHance®) solution, (Bracco Diagnostics Inc., Singen, Germany), prepared in 0.01M PBS. T_1_-weighted images were acquired using a spin echo pulse sequence with the following acquisition parameters. Sequence = rapid acquisition with refocused echoes; echo time = 5.5 milliseconds (ms); effective echo time = 5.5 ms; rapid acquisition with refocused echo factor = 4; repetition time = 557 ms; number of averages = 3; field of view = 18 × 18 mm; matrix size = 256 × 256 pixels; and slice thickness = 0.5 mm. Final contrast-enhanced T_1_-weighted MRI analyses were performed using the NIH ImageJ software. In brief, two regions of interest (ROIs) were drawn on each axial image to cover the entire brain for the analysis (**Figure S15**). One ROI was drawn around the entire right hemisphere (irradiated), while the second ROI was drawn around the entire left hemisphere (control). Bright pixel analysis of the defined ROIs was then performed, as previously reported.^41^, ^51^, ^64^, ^66^, ^67^ A sample size of three (n =3) was used for all contrast-enhanced T_1_-weighted MRI analyses.

All *in vivo* anatomical T_2_-weighted MRIs were acquired using a spin echo pulse sequence with the following acquisitioning parameters: Sequence = rapid acquisition with refocused echoes; echo time = 6.1 ms; effective echo time = 18.3 ms; rapid acquisition with refocused echo factor = 8; repetition time = 1500 ms; number of averages = 2; field of view = 18 × 18 mm; matrix size = 256 × 256 pixels; and slice thickness = 0.5 mm. Final T_2_-weighted MRI analyses were performed with the NIH ImageJ software. Briefly, two ROIs were drawn on each axial image to cover the entire brain for the analysis, as described above (**Figure S15**). T_2_-weighted MRI hyperintensity signal quantification was done using bright pixel analyses, as previously reported.^41^, ^66^ On the other hand, T_2_-weighted MRI hypointensity signal quantification was done using dark pixel analyses, as previously reported.^41^, ^66^ A sample size of three (n =3) was used for all T_2_-weighted MRI analyses.

All *in vivo* T_2_^*^-weighted images were acquired using a gradient echo pulse sequence. Sequence: multiple gradient echoes; echo time = 3 ms; echo spacing = 3.5 ms; number of echoes = 7; effective echo times =3, 6.5, 10, 13.5, 17, 20.5, 24 ms; repetition times = 800 ms; number of averages = 4; number of repetitions = 1; field of view = 18 × 18 mm; matrix size = 128 × 128 pixels; and slice thickness = 0.5 mm. Final T_2_^*^-weighted MRI analyses were performed on the images with an effective echo time of 24 ms, using the NIH ImageJ software. Two ROIs were drawn on each axial image to cover the whole brain for the analyses, as described above (**Figure S15**), and dark pixel analyses was carried out, as previously reported.^41^, ^66^ A sample size of three (n =3) was used for all T_2_^*^-weighted MRI analyses.

### Immunohistochemistry to evaluate the nanoparticle effects on neuroinflammation

At 2 months post-irradiation, mice were transcardially perfused with 30 mL of heparinized PBS (1% heparin in 0.01M PBS), then 30 mL of a 4% paraformaldehyde (PFA) solution (4% PFA in 0.01M PBS), using a flow rate of 10 mL/min. The mouse heads were then stored overnight at 4 ºC in 4% PFA, then at 4 ºC in PBS for 48 hours. The brains were next extracted, paraffin-embedded, and sectioned into 4 µm slices. Histology and immunohistochemistry were next carried out on the tissue sections. For neuroinflammation detection, a rabbit anti-CD68 (1:100, ab283654, Abcam) and a rat anti-IBA1 (1:100, ab283346, Abcam) primary antibody were utilized; followed by an Alexa Fluor® 488 goat anti-rabbit (1:200, ab150077, Abcam) and an Alexa Fluor® 647 goat anti-rat (1:1000, ab150077, Abcam) secondary antibody, respectively. For astrogliosis and neuron loss, a rat anti-GFAP (1:100, ab279291, Abcam) and rabbit Anti NeuN (1:100, ab177487, Abcam) primary antibody were utilized; followed by an Alexa Fluor® 647 goat anti-rat (1:1000, ab150077, Abcam) and an Alexa Fluor® 488 goat anti-rabbit (1:200, ab150077, Abcam) secondary antibody, respectively. The slides were then counter stained with DAPI in fluoroshield mounting media (ab104139, Abcam). All fluorescence image acquisitions were performed using a Zeiss Axio Scan.Z.1 Slide Scanner. Fluorescence image analyses were performed using the NIH ImageJ software. Briefly, two ROIs (**Figure S15**) were drawn for the analysis. Pixel analysis of the defined ROIs was then performed, as previously reported.^41^

### Histological evaluation of nanoparticle toxicity to the liver and kidney

At 2 months post-irradiation, mice were transcardially perfused as described above. The mouse organs were then stored overnight at 4 ºC in 4% PFA, then at 4 ºC in PBS for 48 hours. The mouse livers and kidneys were then paraffin-embedded and sectioned into 4 µm slices. Histology was next carried out on the tissue sections. Briefly, tissue sections were stained with hematoxylin and eosin (H&E) for histology as previously described.^64^ All image acquisitions were performed using a Nano Zoomer S210 Hamamatsu. All image analyses were performed using the NIH ImageJ software and pixel analysis, as previously reported.^41^, ^64^

### Statistical analyses

All data points were presented as the mean ± standard deviation of three independent experiments, except when otherwise stated. All statistical analyses were done using the GraphPad Prism 10.4.1 software. Two dataset comparisons were made using unpaired two-tailed Student's t-tests, except when otherwise stated. Multiple comparisons were made using the ordinary two-way ANOVA test. The results were considered statistically significant at *P* < 0.05.

## Results

### Two block co-polymers were synthesized in high yields

In this study, we designed, synthesized, and characterized two block copolymers possessing varied numbers of BAPE moieties for ROS scavenging (**Figure 1A and 1B**). To evaluate the effects of the number of BAPE moieties in the block copolymers to reduce oxidative stress, a structure-activity study was conducted. Briefly, the block copolymer P2b platform was designed to have twice as many BAPE moieties as the block copolymer P2a platform, (53 BAPE units in P2b versus 26 BAPE units in P2a). Both amphiphilic block copolymers P2a and P2b were designed to also contain a fixed number of polyethylene glycol (PEG) units, for improved circulation in the blood stream.

The block copolymers were synthesized in a three-step reaction via a radical polymerization technique: RAFT polymerization. First, a phenylboronic acid pinacol methacrylate monomer (**BM1**) was synthesized, purified via column chromatography, and obtained in high yield (85%). Next, a PEGylated and NHS ester-functionalized chain transfer agent P(PEGMEA_480_) macro-CTA (**P1**) was synthesized, purified via dialysis, and obtained in high yield (86%). The number-average molecular weight was then determined by end group analysis of the ^1^H NMR spectrum. Briefly, the phenyl protons of the CTA moiety were compared to the terminal methyl protons of the PEGMEA_480_ moiety. The number-average molecular weight was determined to be ~ 7.5 kDa, and the number of PEGMEA_480_ units was determined to be 15. In the third step, the synthesized **BM1**, and 2-(diisopropylamino)ethyl methacrylate (DPA) were incorporated as block segments of the PEGylated chain transfer agent P(PEGMEA_480_) macro-CTA (**P1**) at varying ratios to form two distinct block copolymers. The polymerization reactions were then quenched and dialyzed thoroughly to obtain the purified block copolymers P[PEGMEA_480_-*b*-(DPA-*r*-BM1)], **P2a** and **P2b** respectively. The number-average molecular weight as well as the composition of the synthesized block copolymers were determined by ^1^H NMR spectroscopy from the ratios of the relative intensities of the characteristic protons of the incorporated monomer moieties (**Figure S6**).

### Both block co-polymers formed micellar nanoparticles at low CACs

The CACs of the respective polymers (P2a and P2b) were determined by measuring the respective polymer encapsulation ability of a hydrophobic Nile Red^®^ dye, using fluorescence spectroscopy. Nile Red^®^ has a very weak fluorescence signal in aqueous milieu due to its high hydrophobicity and very low water solubility. In the absence of the polymers, the fluorescence signal of Nile Red® was very low. The fluorescence signal of Nile Red® increased gradually with increasing polymer concentration and then increased drastically once the CAC of the polymer was reached, (as the formation of a polymer hydrophobic core provided stability for the Nile Red^®^ molecules). The CAC was determined from the inflection point of the two distinct Nile Red® fluorescence signal intensity characteristics on a graph of the Nile Red® fluorescence signal intensity versus polymer concentration (**Figure 1C and 1D**). Similar CACs of ~ 0.08 mg/mL were determined for both the block copolymer P2a and P2b (**Figure 1C and 1D**). This indicated that both polymers aggregated to form nanoparticles in aqueous milieu at low polymer concentration of ~ 0.08 mg/mL.

### Both block co-polymers formed spherical micellar nanoparticles

TEM images (**Fig. 1E and 1F**) and SEM images (**Figure 1G and 1H**) revealed that both block co-polymers P2a and P2b respectively formed spherical micellar nanoparticles in aqueous milieu. TEM and SEM images also revealed that the nanoparticles formed by the respective block co-polymers in aqueous milieu were of similar sizes.

### Both block co-polymers formed spherical micellar nanoparticles of similar hydrodynamic diameters, polydispersity indices and zeta potentials

Since both polymeric nanoparticles were designed to possess pH and redox responsive moieties for activation under stimuli (oxidative stress), we evaluated the hydrodynamic diameters and zeta potentials of the respective polymeric nanoparticles both in the absence of stimuli (under physiological conditions at pH 7.4) and in the presence of stimuli (under oxidative stress, demonstrated with 0.5 mM of H_2_O_2_ at pH 6.2).

Under physiological conditions (pH 7.4), both nanoparticles had comparable hydrodynamic diameters, polydispersity indices and zeta potentials (**Figure 1I, 1J and Table 1**). Hydrodynamic diameters of 166 ± 51 nm and 183 ± 54 nm were measured for nanoparticles P2a and P2b respectively. The slightly larger hydrodynamic diameter of nanoparticle P2b was attributed it having more BAPE moieties than nanoparticle P2a (53 BAPE units in P2b versus 26 BAPE units in P2a). Polydispersity indices of 0.073 ± 0.02 and 0.087 ± 00 were measured for nanoparticles P2a and P2b respectively. These polydispersity indices below 0.1 indicated the uniformity of the respective nanoparticle solutions.^68^ Finally, zeta potentials of **-**2.52 ± 0.658 mV and **-**2.89 ± 1.58 mV were measured for nanoparticles P2a and P2b respectively. The negative zeta potentials of the respective nanoparticles could limit their non-specific uptake in healthy off-target tissues.

Under oxidative stress, the hydrodynamic diameters of the activated/degraded nanoparticles were significantly reduced to 30.7 ± 22.6 nm and 25.6 ± 3.6 nm for nanoparticles P2a and P2b, respectively (**Figure 1K, 1L and Table 1**). Polydispersity indices of 0.92 ± 0.11 and 1.00 ± 0.00 were also measured for the activated/degraded nanoparticles P2a and P2b respectively. These polydispersity indices close to one indicated highly heterogenous activated/degraded nanoparticle solutions.^68^ Furthermore, zeta potentials of **+** 4.82 ± 3.01 mV and **+** 2.99 ± 0.97 mV were measured for activated/degraded nanoparticles P2a and P2b respectively.

### Both micellar nanoparticles were stable after serial dilution at pH 7.4

Nanoparticle dilution occurs after intravenous nanoparticle administration into mice or humans. This nanoparticle dilution could lead to the destabilization of some polymeric micellar nanoparticles. Thus, a nanoparticle dilution study was carried out to determine the colloidal stability of the respective polymeric micellar nanoparticles under physiological conditions (pH 7.4). Overall, no changes in the hydrodynamic diameters of the respective nanoparticles were detected after dilution of either of the nanoparticles up to 100 times using PBS at pH 7.4 (**Figure 1M and 1N**). This showed that both micellar nanoparticles P2a and P2b were stable after dilution and could be administered intravenously into mice and humans.

### Fluorescence spectroscopy revealed that the activation of both nanoparticles was faster under oxidative stress

To determine the activation kinetics of the polymeric nanoparticles in the presence and absence of stimuli (oxidative stress), Nile Red^®^ was first encapsulated in the respective polymeric nanoparticles (**Figure 2A**). The kinetics of the Nile Red^®^ dye's release from the respective nanoparticles maintained under different conditions of stimuli activation (different pH values and concentrations of H_2_O_2_) were then measured by quantifying the free Nile Red^®^ dye in solution using fluorescence spectroscopy.

The activation kinetics of the respective nanoparticles under different conditions of oxidative stress (demonstrated using varying concentrations of H_2_O_2_ and at pH 6.2) were compared to the activation kinetics of the respective nanoparticles under physiological conditions (demonstrated using pH 7.4 and the absence of H_2_O_2_). Overall, the activation kinetics of both nanoparticles (**Figure 2B and 2C**) were faster at pH 6.2 (under oxidative stress) compared to at pH 7.4 (under normal physiological). Additionally, the activation kinetics of both nanoparticles (**Figure 2B and 2C**) were faster at higher concentrations of H_2_O_2_ (higher oxidative stress) than at lower concentrations of H_2_O_2_ (lower oxidative stress). For instance, at pH 6.2, the activation kinetics at 1.0 mM H_2_O_2_ > activation kinetics at 0.5 mM H_2_O_2_ > activation kinetics at 0.1 mM H_2_O_2_.

Interestingly, whereas the activation kinetics of nanoparticle P2a was significantly faster than that of nanoparticle P2b at physiological conditions (*P* = 0.0024, n = 3); the activation kinetics of nanoparticle P2b was significantly faster (*P* < 0.05, n = 3) than that of P2a under oxidative stress (**Figure S16**). More specifically, at pH 7.4, 22.92% ± 0.57% and 21.97% ± 0.85% of the encapsulated Nile Red^®^ dye had been released from nanoparticle P2a and P2b respectively, within 15 days (360 h) of nanoparticle activation. However, at pH 6.2 and 0.1 mM H_2_O_2_, 49.23% ± 0.93% and 56.51% ± 0.78% of the encapsulated Nile Red^®^ dye had been released from nanoparticle P2a and P2b respectively, within 15 days of nanoparticle activation. At pH 6.2 and 0.5 mM H_2_O_2_, 57.36% ± 0.93% and 70.36% ± 1.12% of the encapsulated Nile Red^®^ dye had been released from nanoparticle P2a and P2b respectively, within 15 days of nanoparticle activation. At pH 6.2 and 1.0 mM H_2_O_2_, 75.49% ± 0.63% and 76.00% ± 0.80% of the encapsulated Nile Red^®^ dye had been released from nanoparticle P2a and P2b respectively, within 15 days (360 h) of nanoparticle activation/degradation.

### Fluorescence imaging confirmed that the release of the nanoparticle-encapsulated dye was faster under oxidative stress

Fluorescence images of agarose gel phantoms on which the 700 nm fluorescently labelled and 800 nm fluorophore-encapsulated nanoparticles P2a and P2b were respectively placed revealed that the release of the encapsulated fluorophore was faster under oxidative stress than under normoxic conditions (**Figure 2D and 2E**).

Graphs of the 700 nm fluorescence signal changes along the length of the agarose gel phantom tubes kept at different conditions of oxidative stress were used to evaluate the diffusion of the respectively activated 700 nm fluorescently labelled nanoparticles (**Figure 2F**). A high 700 nm fluorescence signal was detected at the top of the agarose gel tubes (0.0 to 0.1 inches), over the 15-day imaging period. This indicated that the activated nanoparticles did not significantly diffuse along the length of the agarose gel phantom tubes over the 15-day imaging period, due to the high molecular weights of the nanoparticles.

Graphs of the 800 nm fluorescence signal changes along the length of the agarose gel phantom tubes kept under different conditions of oxidative stress, were used to evaluate the release and diffusion of the encapsulated 800 nm fluorophore from the activated nanoparticles over time (**Figure 2G**). The shift in the 800 nm fluorescence signal along the length of the agarose gel phantom tubes over the 15-day imaging period indicated the release and diffusion of the encapsulated 800 nm fluorophore over time. The release and diffusion of the encapsulated 800 nm fluorophore was highly dependent on the nanoparticle activation conditions. Overall, faster release and diffusion rates of the 800 nm fluorophore were detected at pH 6.2 (indicative of oxidative stress) compared to pH 7.4 (indicative of physiological conditions) for both nanoparticles P2a and P2b. Faster release and diffusion rates of the 800 nm fluorophore were also detected at higher H_2_O_2_ concentrations, indicative of the oxidative stress severity (1 mM H_2_O_2_ > 0.5 mM H_2_O_2_ > 0.1 mM H_2_O_2_ > 0.0 mM H_2_O_2_) for both nanoparticles P2a and P2b. Interestingly, overall the 800 nm fluorophore release and diffusion rates (clearance kinetics) from the activated nanoparticle P2a were two-folds slower than those from nanoparticle P2b (**Figure 2G and Figure S17**). This suggested that the cargo released from nanoparticle P2a might be better retained in its adjacent tissues, than that from P2b.

### The nanoparticles protected the RPE fluorescent protein from degradation under oxidative stress

The ability of the nanoparticles to protect the fluorescent RPE protein from degradation under oxidative stress was evaluated using a fluorescence assay (**Figure 3A**). The fluorescence spectra of a 42.5 nM (0.0085 nmol) RPE solution showed that in the absence of oxidative stress, the RPE fluorescence signal decreased by 22.29% ± 1.2 8%; while in the presence of oxidative stress, the RPE fluorescence signal decreased two-folds more, 56.73% ± 5.58% (**Figure 3B and Figure S18**). In the presence of oxidative stress and a negative control agent PEG (10 µg, 1.33 nmol) the RPE fluorescence signal still decreased two-folds more, 57.05% ± 3.41% (**Figure 3C, 3D and Figure S18**). This showed that PEG was ineffective at protecting the RPE protein from degradation under oxidative stress. However, in the presence of oxidative stress and nanoparticle P2a (1 µg, 0.034 nmol), the RPE fluorescence signal decreased to 21.53% ± 1.21% (**Figure 3C and Figure S18**). This showed that nanoparticle P2a was effective at protecting the RPE protein from degradation under oxidative stress. In the presence of oxidative stress and nanoparticle P2b (1 µg, 0.0271 nmol), the RPE fluorescence signal decreased to 19.59% ± 1.40% (**Figure 3D and Figure S18**). This also showed that nanoparticle P2b was effective at protecting the RPE protein from degradation under oxidative stress. As little as 1 µg of either nanoparticle P2a (0.034 nmol) or P2b (0.0271 nmol) was sufficient to prevent the degradation of 42.5 nM (0.0085 nmol) of RPE. Increasing the amount of either nanoparticles to 2 µg, (0.068 nmol for P2a or 0.0542 nmol for P2b) or 4 µg (0.135 nmol for P2a or 0.108 nmol for P2b) had the same protective effect on the RPE solution (**Figure 3C, 3D and Figure S18**). This fluorescence assay showed the protective effects of both polymeric nanoparticles P2a and P2b.

### The nanoparticles were taken up by endothelial cells and were not toxic to the endothelial cells

Both nanoparticles P2a and P2b were taken up by HUVECs at all nanoparticle concentrations tested, in a concentration dependent manner (**Figure 3E and 3F**). However, the uptake of nanoparticle P2b was statistically higher than that of P2a at lower nanoparticle concentrations of 6.25 µg/µL (*P* < 0.05), but not at higher nanoparticle concentrations. Briefly, at nanoparticle concentrations of 6.25 µg/µL, 20.80% ± 1.49% and 48.53% ± 7.82% of HUVECs contained nanoparticle P2a and P2b respectively. At nanoparticle concentrations of 12.5 µg/µL, 55.23% ± 10.53% and 75.00% ± 1.44% of HUVECs contained nanoparticle P2a and P2b respectively. And at nanoparticle concentrations of 25 µg/µL, 72.18% ± 4.56% and 80.75% ± 7.54% of HUVECs contained nanoparticle P2a and P2b respectively.

Additionally, both nanoparticles were not cytotoxic to HUVECs at any of the nanoparticle concentrations tested (**Figure 3G**). Briefly, the MTS assay showed > 90% HUVEC viability after 24 hours of incubation with either nanoparticles P2a or P2b at all the nanoparticle concentrations tested (6.25 µg/µL, 12.5 µg/µL, or 25 µg/µL).

### Fluorescence imaging revealed the accumulation and retention of both nanoparticles in irradiated lesions of the brain for at least seven days

Fluorescence images of the IRDye^®^ 800CW-conjugated polymeric nanoparticles administered intravenously to the irradiated mice at 2 weeks post-irradiation, showed statistically significant accumulation of the respective nanoparticles in the irradiated brain lesions at 4 hours post-administration (**Figure 4A, 4B, 4C, Table S1, S2 and S3**). The 820 nm fluorescence signals from the respective nanoparticles were detected in the irradiated brain lesions throughout the seven-day imaging period (**Figure 4A, 4B, 4C, Table S1, S2 and S3**). By comparing the ratio of the fluorescence signal detected from the irradiated brain lesion ROI to that detected from a non-irradiated region ROI of the body (**Figure S15**), a ratio of 1.64 ± 0.41 was detected in the mice before nanoparticle administration. Ratios of 3.27 ± 0.59 and 3.90 ± 0.33 were detected at 4 h after the administration of nanoparticles P2a and P2b, respectively. These ratios remained constant throughout the seven-day imaging period and ratios of 4.07 ± 0.17 and 3.73 ± 0.15 could still be detected at seven days after the administration of nanoparticles P2a and P2b, respectively. An 820 nm fluorescence signal was also detected from the abdomen of the mice throughout the seven-day imaging period, indicating nanoparticle uptake in the liver (**Figure S19**).

### Biodistribution analyses of *ex vivo* fluorescence images revealed that approximately 1% of the administered nanoparticles P2a and P2b were detected in the irradiated brain lesions at 4 h and 24 h post-administration

Biodistribution studies of the nanoparticles were carried out using IRDye^®^ 800CW-conjugated and IRDye^®^ 680RD-encapsulated nanoparticles. The 820 nm fluorescence images showed that 1.1% ± 0.2% and 0.9% ± 0.2% of the respective nanoparticles P2a and P2b were detected in the irradiated brain lesions at 4 h post-administration (**Figure 4D - 4H and Figure S20**). Similar nanoparticle percentages were detected in the irradiated brain lesions at 24 h after nanoparticle administration (**Figure 4D - 4H and Figure S20**). These nanoparticle percentages corresponded to ~ 0.2 µg of the respective nanoparticles being delivered to the irradiated lesions of the brain. These nanoparticle percentages in the irradiated brain lesions were ~ 3-fold higher than those in the brains of non-irradiated control mice that received the nanoparticles (**Figure S21**). Much higher nanoparticle concentrations were detected in the liver (52.5% ± 0.1% and 32.9% ± 4.8% for P2a and P2b, respectively), and kidneys (37.8% ± 1.1% and 58.9% ± 3.7% for P2a and P2b, respectively) than in the irradiated brain lesions at 4 h after nanoparticle administration. The respective nanoparticle percentages in the liver and kidneys were similar at 4 h and 24 h after nanoparticle administration (**Figure 4D - 4H, and Figure S20**).

The 720 nm fluorescence images could not be used to effectively quantify the *in vivo* release of the encapsulated IRDye^®^ 680RD dye from the activated nanoparticles, due to the high inherent background from the tissues at this wavelength (**Figure S22**).

### The nanoparticles were not toxic to mice

Complete blood count analyses and comprehensive serum chemistry tests were conducted to evaluate the potential systemic toxicity of the respective nanoparticles at a dose of 0.8 mg/kg (200 μL, 0.1 µg/µL; or 20 µg), at 15 days after the intravenous administration of the respective nanoparticles. No statistically significant differences were detected between any of the mouse groups evaluated in either the complete blood count tests (**Figure 5A**) or the comprehensive serum chemistry tests (**Figure 5B**). These findings confirmed the safety of both nanoparticles P2a or P2b at the dose evaluated.

Additionally, H&E histology conducted on the kidney and liver tissues harvested from the mice at 1.5 months after nanoparticle administration showed no signs of kidney or liver toxicity (**Figure 6A - 6B**), despite high nanoparticle accumulation in these organs. This further confirmed the safety of both nanoparticles P2a or P2b.

Furthermore, no statistically significant weight differences were detected between any of the irradiated mouse groups during the 2-month study period (**Figure 6C**). This further suggested no toxicity from either nanoparticle P2a or P2b, at the dose tested. However, a statistically significant difference was detected between the non-irradiated mouse group and the irradiated mouse groups (*P* < 0.05). This suggested radiation-induced toxicity in the irradiated mice.

### *In vivo* multi-parametric MRI revealed reduced BBB permeability and edema in the irradiated and nanoparticle-treated mice compared to the irradiated control mice

*In vivo* contrast-enhanced T_1_W MRI conducted at two months post-irradiation (and 1.5 months after nanoparticle administration) showed BBB permeability in all the irradiated mouse groups (**Figure 7A, 7B, and Table S4**). However, as expected, no BBB permeability was detected in the non-irradiated mice (**Figure 7A, 7B, and Table S4**). Interestingly, the degree of BBB permeability was less in the irradiated mice that received nanoparticle P2b compared to the irradiated mice that received either PBS or nanoparticle P2a (**Figure 7A, 7B, Table S4 and S5**). Quantitative bright pixel analysis of the contrast-enhanced T_1_W MRI signal ratios of the irradiated brain hemispheres compared to the non-irradiated contralateral brain hemispheres revealed a ratio of 10.72 ± 1.75 in the irradiated mice that received nanoparticle P2b (**Figure 7E, Table S4 and S5**), compared to 14.56 ± 0.28 in the irradiated mice that received PBS (*P* = 0.0198, n = 3); and 15.74 ± 1.46-fold in the irradiated mice that received nanoparticle P2a (*P* = 0.0187, n = 3). This indicated that nanoparticle P2b (with more ROS scavengers) was more effective at reducing BBB permeability than nanoparticle P2a (with less ROS scavengers). Mechanistically, the higher number of ROS scavengers (53) released from nanoparticle P2b and their faster rate of release under oxidative stress putatively more effectively protected the endothelial cells of the BBB from radiation-induced damage than the lower number of ROS scavengers (26) released from nanoparticle P2a at a slower release rate under oxidative stress.

*In vivo* T_2_W MRI also conducted at two months post-irradiation (and 1.5 months after nanoparticle administration) revealed statistically less hyperintensity (indicative of less edema), in the irradiated mice that received either nanoparticle P2a or P2b than in the irradiated mice that received PBS (**Figure. 7C, 7E, Table S6 and S7**). As expected, no T_2_W MRI hyperintensity was detected in the non-irradiated control mice (**Figure 7C, 7E, and SI Table S6**). Quantitative bright pixel analysis of the T_2_W MRI hyperintensity signal ratios of the irradiated brain hemispheres compared to the non-irradiated contralateral brain hemispheres revealed a ratio of 2.90 ± 0.43 in the irradiated mice that received PBS (**Figure 7E, Table S6 and S7**), compared to 1.46 ± 0.24 in the irradiated mice that received nanoparticle P2a (*P* = 0.0071, n = 3); and 1.48 ± 0.39 in the irradiated mice that received nanoparticle P2b (*P* = 0.0130, n = 3). These ratios detected in the irradiated mice that received either nanoparticles P2a or P2b were comparable to those detected in the non-irradiated control mice (1.07 ± 0.34). This indicated that both nanoparticles P2a and P2b were effective in reducing radiation-induced edema (**Figure 7C, 7E, Table S6 and S7**). Thus mechanistically, we postulated that the ROS scavengers released from both nanotheranostic agents effectively reduced radiation-induced chronic oxidative stress, which resulted in reduced edema in the irradiated tissues.

No significant differences were detected between the different mouse groups using the less sensitive T_2_W MRI hypointense signal (**Figure 7C, 7E, Table S8 and S9**).

*In vivo* T_2_^*^W MRI at two months post-irradiation (and 1.5 months after nanoparticle administration) showed significant hemorrhage (hypointensity) in all the irradiated mouse groups compared to the non-irradiated mice (**Figure 7D, 7E, Table S10, and S11**). Quantitative dark pixel analysis of the T_2_^*^W MRI signal ratios of the right brain hemispheres compared to the left contralateral brain hemispheres revealed a ratio of 1.17 ± 0.41 in the non-irradiated mice (**Figure 7E, Table S10, and S11**), compared to ratios of 4.44 ± 1.10 in the irradiated mice that received PBS (*P* = 0.0085, n = 3); 5.06 ± 0.24 in the irradiated mice that received nanoparticle P2a (*P* = 0.0001, n = 3); and 4.11 ± 1.43 in the irradiated mice that received nanoparticle P2b (*P* = 0.0268, n = 3). This indicated that at the nanotheranostic agent doses evaluated (0.8 mg/Kg, 20 µg) neither nanoparticle P2a nor P2b effectively reduced radiation-induced hemorrhage at the intermediate stages of RIBI (**Figure 7E, Table S10, and S11**). Thus, we are currently evaluating the feasibility of using higher nanotheranostic agent doses and multiple dose treatment regiments to reduce radiation-induced hemorrhage in RIBI.

### CD68 and IBA1 immunohistochemistry of the brain tissue samples showed reduced neuroinflammation in the irradiated and nanoparticle-treated mice compared to the irradiated control mice

CD68 immunohistochemistry conducted at two months post-irradiation (and 1.5 months after nanoparticle administration) showed significantly more CD68 (green) expression in all the irradiated mice compared to the non-irradiated control mice (**Figure 8A**). Quantification of the respective CD68 fluorescence signals in the right brain hemisphere normalized to the left contralateral brain hemisphere of the same mouse (**Figure 8B, Table S12, and S13**), gave a ratio of 1.08 ± 0.43 in the non-irradiated mice compared to ratios of 22.95 ± 2.35 in the irradiated mice that received PBS (*P* = 0.00009, n = 3); 20.79 ± 2.05 in the irradiated mice that received nanoparticle P2a (*P* = 0.00008, n = 3); and 4.36 ± 1.83 in the irradiated mice that received nanoparticle P2b (*P* = 0.03913, n = 3). Interestingly, a statistically significant difference in CD68 staining was also detected between the irradiated mice that received nanoparticle P2b and the irradiated mice that received either PBS (*P* = 0.0004, n = 3) or nanoparticle P2a (*P* =0.0005, n = 3). This suggested that nanoparticle P2b was more effective at controlling CD68 neuroinflammation (the infiltration of peripheral macrophages) than nanoparticle P2a. These CD68 findings correlated with the contrast-enhanced T_1_W MRI results, which showed that nanoparticle P2b (with more ROS scavengers) was more effective in reducing BBB permeability than nanoparticle P2a (with less ROS scavengers). Mechanistically, this suggested that the higher number of ROS scavengers (53) released from nanoparticle P2b could putatively more effectively protect the endothelial cells of the BBB from radiation-induced damage than the lower number of ROS scavengers (26) released from nanoparticle P2a. This reduced BBB permeability in the P2b-treated mouse group further resulted in less infiltration of CD68-positive peripheral macrophages into the irradiated brain lesions of the mice, compared to the P2a-treated mouse group.

IBA1 immunohistochemistry also showed significantly more IBA1 (red) expression in the irradiated mice that received PBS or nanoparticle P2a compared to the non-irradiated control mice at 1.5 months after nanoparticle administration (**Figure 8A**). Interestingly, no statistically significant differences in IBA1 (red) expression were detected at 1.5 months after nanoparticle administration in the irradiated mice that received nanoparticle P2b compared to the non-irradiated control mice. Quantification of the respective IBA1 fluorescence signals in the right brain hemisphere normalized to the left contralateral brain hemisphere of the same mice (**Figure 8B, Table S14, and S15**), at 1.5 months after nanoparticle administration gave a ratio of 1.20 ± 0.54 in the non-irradiated control mice compared to ratios of 6.25 ± 0.51 in the irradiated mice that received PBS (*P* = 0.0003, n = 3); 3.88 ± 0.22 in the irradiated mice that received nanoparticle P2a (*P* = 0.0014, n = 3); and 1.98 ± 0.27 in the irradiated mice that received nanoparticle P2b (*P* = 0.0912, n = 3). Interestingly, a statistically significant difference in IBA1 staining was also detected between the irradiated mice that received PBS and the irradiated mice that received either nanoparticle P2a (*P* = 0.0018, n = 3) or P2b (*P* = 0.0002, n = 3). Furthermore, a statistically significant difference in IBA1 staining was also detected between the irradiated mice that that received nanoparticle P2a and the irradiated mice that received nanoparticle P2b (*P* = 0.0007, n = 3). This suggested that although both nanoparticle P2a and P2b were effective at controlling microglial activation (IBA1), nanoparticle P2b was more effective at controlling microglial activation than nanoparticle P2a. Mechanistically, the higher number of ROS scavengers (53) released from nanoparticle P2b putatively more effectively reduced radiation-induced chronic oxidative stress than the lower number of ROS scavengers (26) released from nanoparticle P2a. Ultimately, this resulted in lesser microglial activation in the P2b-treated mice compared to the P2a-treated mice.

### GFAP immunohistochemistry of brain tissue samples showed reduced astrogliosis in the irradiated and nanoparticle-treated mice compared to the irradiated control mice

GFAP immunohistochemistry (**Figure 9A**) showed statistically more GFAP (red) expression in all the irradiated mice compared to the non-irradiated mice at two months post-irradiation (and 1.5 months after nanoparticle administration). Quantification of the respective GFAP fluorescence signals in the right brain hemisphere normalized to the left contralateral brain hemisphere of the respective mice at 1.5 months after nanoparticle administration (**Figure 9B, Table S16, and S17**), gave a ratio of 0.78 ± 0.13 in the non-irradiated mice compared to ratios of 23.14 ± 4.66 in the irradiated mice that received PBS (*P* = 0.0011, n = 3); 5.53 ± 0.92 in the irradiated mice that received nanoparticle P2a (*P* = 0.0009, n = 3); and 3.48 ± 0.36 in the irradiated mice that received nanoparticle P2b (*P* = 0.0003, n = 3). Interestingly, a statistically significant difference in GFAP staining was also detected between the irradiated mice that received nanoparticle P2b and the irradiated mice that received either PBS (*P* = 0.0030, n = 3) or nanoparticle P2a (*P* = 0.0019, n = 3). This suggested that although both nanoparticle P2a and P2b were effective at controlling astrogliosis (GFAP), nanoparticle P2b was more effective than nanoparticle P2a. Mechanistically, the higher number of ROS scavengers (53) released from nanoparticle P2b more effectively reduced radiation-induced chronic oxidative stress than the lower number of ROS scavengers (26) released from nanoparticle P2a. Ultimately, this resulted in reduced astrocyte activation in the P2b-treated mice compared to the P2a-treated mice.

No statistically significant change in the NeuN (green) fluorescence signal was detected in the irradiated brain hemispheres compared to the non-irradiated brain hemispheres over the 2-month post-irradiation study period (**Figure 9A and 9B**). This indicated no changes in neuronal cell death within this time frame.

## Discussion

In this study, we designed, synthesized, characterized, formulated, and preclinically evaluated two oxidative stress-responsive polymeric nanotheranostic agents, possessing varied numbers of BAPE moieties for ROS scavenging and the sustained reduction of neuroinflammation, in our previously developed preclinical mouse model of RIBI. Overall, both block co-polymers P2a and P2b were synthesized in high yield (> 75%) and formed polymeric micellar nanoparticles in aqueous milieu at low polymer concentrations (~ 0.08 mg/mL). Additionally, both nanoparticles were stable at up to 100-fold dilution, confirming their ability to be administered intravenously.

Phantom *ex vivo* studies confirmed that the activation of both nanotheranostic agents was higher under oxidative stress than under normal physiological conditions. This suggested that the nanotheranostic agents would be activated mainly in injured/diseased tissues with high oxidative stress, but not in healthy tissues with low oxidative stress. Ultimately, this could minimize off target side effects from the agents. Additionally, as little as 1 µg of either of the nanotheranostic agents was sufficient to protect the fluorescent R-phycoerythrin protein from degradation under oxidative stress. Furthermore, both nanotheranostic agents were effectively taken up by endothelial cells (which are vulnerable to radiation), and the nanotheranostic agents were not toxic to these endothelial cells. Complete blood count tests and comprehensive serum chemistry tests also showed that the nanotheranostic agents were not toxic at the dose used in the study (0.8 mg/Kg). Histological evaluation of mouse organs (livers and kidneys) at 1.5 months after nanotheranostic agent administration, also confirmed that the nanotheranostic agents were not toxic at the dose used in the study.

Fluorescence imaging showed that ~ 1% (~ 0.2 µg) of the respectively injected nanotheranostic agents were detected in the irradiated brain lesions of mice at four hours after the intravenous administration of 20 µg (0.8 mg/Kg) of the nanotheranostic agents. Additionally, the nanotheranostic agents were retained in the irradiated brain lesions for at least seven days after nanoparticle administration. This prolonged retention of the nanotheranostic agents in the irradiated brain lesions enabled them to sustainably control chronic oxidative stress and neuroinflammation. Anatomical T_2_W MRIs that were acquired at 1.5 months after nanotheranostic agent administration showed significantly reduced brain edema (hyperintensity) in the irradiated mice that were treated with either nanotheranostic agents P2a or P2b compared to the irradiated mice that were treated with PBS. Additionally, contrast-enhanced T_1_W MRIs acquired at 1.5 months after nanotheranostic agent administration showed that the nanotheranostic agent P2b was more effective than the nanotheranostic agent P2a at reducing BBB permeability.

Immunohistochemistry performed at 1.5 months after nanotheranostic agent administration also confirmed significantly reduced microglial activation (IBA1) in the irradiated mice that were treated with either nanotheranostic agents P2a or P2b compared to the irradiated mice that were treated with PBS. However, the nanotheranostic agent P2b was more effective than the nanotheranostic agent P2a at reducing microglial activation. Additionally, CD68 immunohistochemistry showed that the nanotheranostic agent P2b was more effective than the nanotheranostic agent P2a at reducing the infiltration of peripheral macrophages into the brain (CD68 expression). Furthermore, significantly reduced astrogliosis (GFAP expression) was detected in the irradiated mice that were treated with either nanotheranostic agents P2a or P2b compared to the irradiated mice that were treated with PBS. However, the nanotheranostic agent P2b was more effective than the nanotheranostic agent P2a at reducing astrogliosis.

Taken together, our results showed that although both nanotheranostic agents P2a and P2b reduced neuroinflammation at the intermediate phases of RIBI, nanotheranostic agent P2b that possessed twice as many ROS scavengers released at a 5% to 13% faster rate (under oxidative stress) was more effective at mitigating neuroinflammation at the intermediate phases of RIBI than nanotheranostic agent P2a. Mechanistically, this resulted in nanotheranostic agent P2b more effectively controlling oxidative stress and protecting the endothelial cells of the BBB from radiation-induced damage better than nanotheranostic agent P2a. This putatively reduced chronic oxidative stress in the irradiated P2b-treated mice resulted in turn to reduced BBB permeability and the reduced infiltration of CD68-positive peripheral macrophages, than in irradiated P2a-treated mice. This reduced chronic oxidative stress also resulted in reduced microglia and astrocyte activation in the irradiated P2b-treated mice compared to irradiated P2a-treated mice. Collectively, these findings also suggested that these nanotheranostic agents that significantly reduced neuroinflammation and astrogliosis at the intermediate phases of RIBI could also reduce long-term cognitive impairment in RIBI. Thus, we are currently evaluating the dose-dependent effects of the nanotheranostic agents on mitigating long-term cognitive impairment.

Given the findings from this study, we postulate that nanotheranostic agent P2b (with a higher number of ROS scavengers released at a faster rate under oxidative stress) might be more suitable and effective at controlling high levels of acute oxidative stress and neuroinflammation; while nanotheranostic agent P2a (with a lower number of ROS scavengers released at a slower rate under oxidative stress) might be more suitable and effective at controlling low levels of chronic oxidative stress. Thus, we are currently evaluating the long-term therapeutic efficacies of nanotheranostic agents P2a and P2b in a long-term study (12-month post-irradiation).

Despite the demonstrated therapeutic benefits of the developed nanotheranostic agents, there are a few limitations associated with the preclinical model of RIBI that was used to evaluate the agents.^41^ These include: 1) A non-tumor bearing mouse model was used to simplify the study and eliminate any potentially confounding complexities of variable tumor responses to radiotherapy. 2) Since in human brain tumor survivors, RIBI is usually detected several years (and sometimes even decades) after radiotherapy, to compensate for the relatively shorter lifespans of mice compared to humans, a radiation dose higher than that typically used in the clinical setting was used to accelerate the occurrence of RIBI.^69^ This enabled the detection of the three clinical phases of RIBI (acute, early delayed, and late phase) and the treatment of RIBI within the relatively short lifespan of the mice. 3) Given the relatively small size of the mouse brain, a single radiation dose regiment was used to minimize variable irradiation of the target region, as opposed to the fractionated radiation regimens typically used in clinical settings. Thus, our future studies will include evaluating these nanotheranostic agents in radiation-responsive brain tumor mouse models of RIBI, prior to clinical translation.

## Conclusion

Chronic oxidative stress and neuroinflammation are key drivers of RIBI. In this study, we showed that oxidative stress-responsive polymeric nanotheranostic agents can be used to sustainably reduce radiation-induced neuroinflammation after radiotherapy. These nanotheranostic agents are safe and effective and could provide a means to sustainably control neuroinflammation in pediatric brain tumor survivors who were treated with radiotherapy at a young age and face the risk of developing RIBI later-on in life. Ultimately, these nanotheranostic agents could provide a means to sustainably prevent RIBI-associated neuroinflammation in pediatric brain tumor survivors. Furthermore, these sustained release nanotheranostic agents could improve the adherence of patients with memory problems to treatment regiments/schedules and ultimately improve RIBI treatment outcomes.

## Supplementary Material

Supplementary schemes.

## Figures and Tables

**Figure 1:**
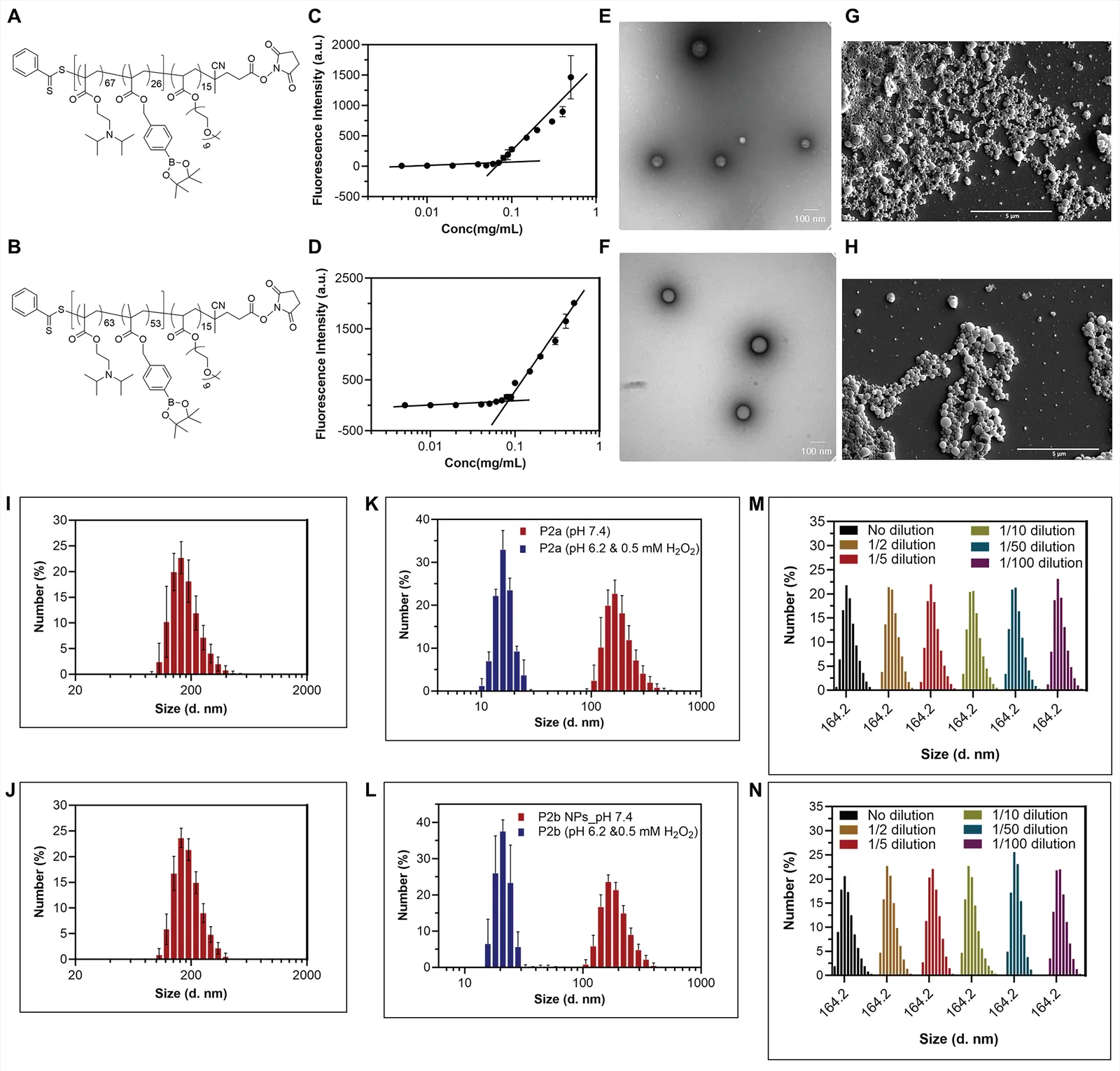
** Structure and characterization of polymeric micellar nanoparticles.** Structure of block co-polymers P2a (**A**) and P2b (**B**). The block co-polymer P2b was designed to have twice as many BAPE ROS scavenging moieties as the block copolymer P2a. Determination of the CAC of block co-polymers P2a (**C**) and P2b (**D**). Similar CACs of ~ 0.08 mg/mL were determined for both the block copolymer P2a and P2b. This indicated that both polymers readily formed nanoparticles in aqueous milieu at low polymer concentrations. TEM images of nanoparticles P2a (**E**) and P2b (**F**). SEM images of nanoparticles P2a (**G**) and P2b (**H**). Both block co-polymers P2a and P2b formed spherical micellar nanoparticles in aqueous milieu. Hydrodynamic diameters of nanoparticle P2a (**I**) and P2b (**J**) at physiological pH (pH of 7.4). Hydrodynamic diameters of 166 ± 51 nm and 183 ± 54 nm were measured for nanoparticles P2a and P2b respectively, at physiological pH. The slightly larger hydrodynamic diameter of nanoparticle P2b was attributed it having more BAPE moieties than nanoparticle P2a. Hydrodynamic diameters of nanoparticle P2a (**K**) and P2b (**L**) at physiological pH compared to under oxidative stress (demonstrated by pH of 6.2 and 0.5 mM H_2_O_2_). The hydrodynamic diameters of the activated/degraded nanoparticles were significantly reduced to 30.7 ± 22.6 nm and 25.6 ± 3.6 nm for nanoparticles P2a and P2b, respectively, under oxidative stress. Stability of the nanoparticles P2a (**M**) and P2b (**N**) at pH 7.4. Both micellar nanoparticles P2a and P2b were stable after 100-fold dilution. This indicated that both nanoparticles were sufficiently stable and could be administered intravenously into mice and humans.

**Figure 2:**
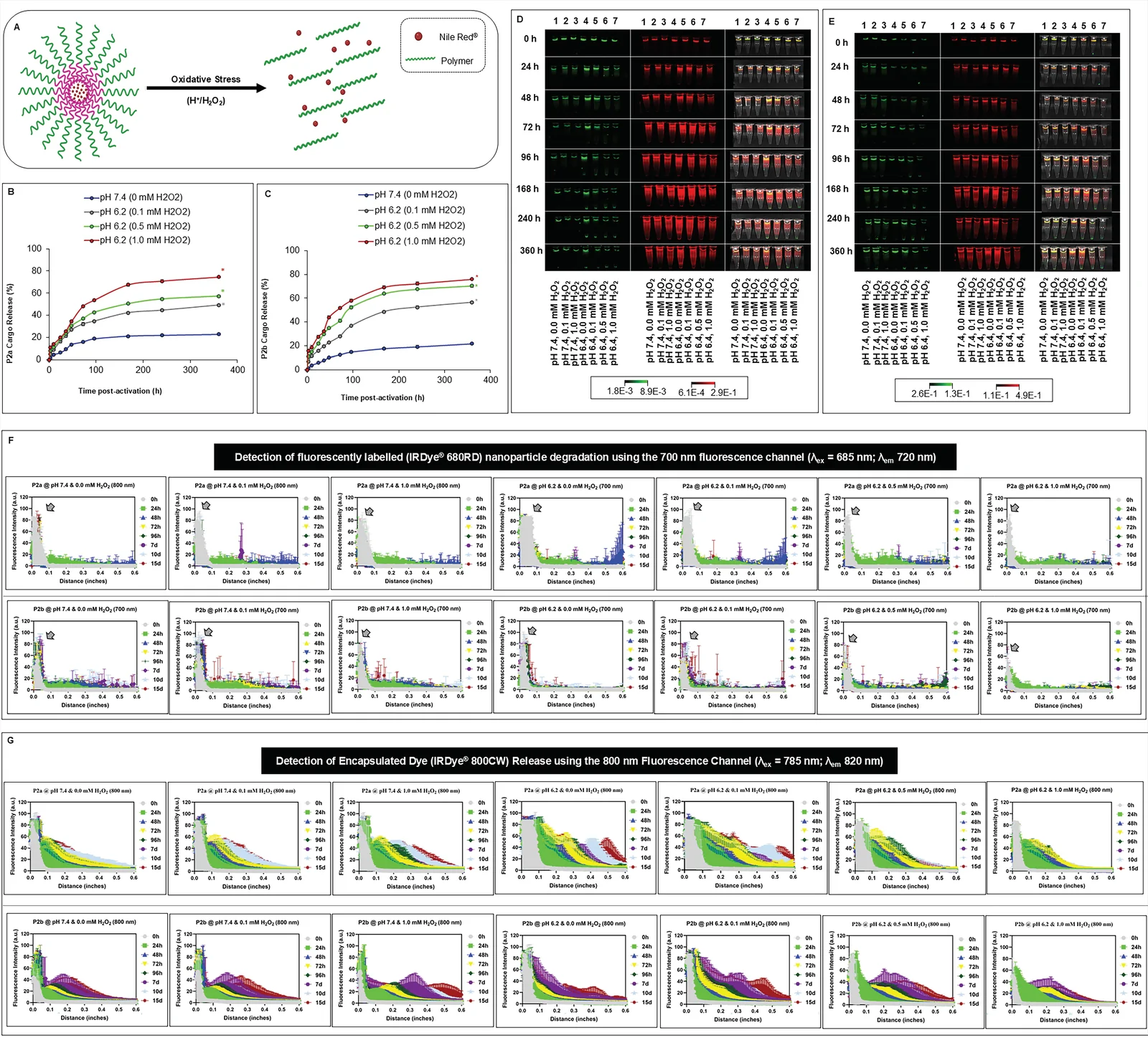
** Kinetics of polymeric nanoparticle activation and cargo (fluorophore) release**. **A**) Schematic of nanoparticle activation and encapsulated fluorophore release from the activated nanoparticle under oxidative stress (H^+^/ H_2_O_2_). Release kinetics of an encapsulated fluorophore (Nile Red®) under varying degrees of oxidative stress (demonstrated by varying concentrations of H_2_O_2_ at pH 6.2) compared to under physiological conditions (demonstrated by pH 7.4 and no H_2_O_2_) from nanoparticle P2a (**B**) and P2b (**C**) measured by fluorescence spectroscopy (p ≤ 0.05). The activation kinetics of the respective nanoparticles were faster under oxidative stress (pH 6.2) compared to under normoxic conditions (pH 7.4). Additionally, the activation kinetics of the respective nanoparticles were faster at higher degrees of oxidative stress (higher concentrations of H_2_O_2_) than at lower degrees of oxidative stress (lower concentrations of H_2_O_2_). Fluorescence images of agarose gel phantoms under different conditions of oxidative stress, on which the 700 nm fluorescently labelled and 800 nm fluorophore-encapsulated nanoparticles p2a (**D**) and P2b (**E**) were placed. Fluorescence images were acquired over a 15-day (360 h) post-placement period to evaluate the nanoparticle activation kinetics and the release and clearance kinetics of the 800 nm encapsulated fluorophore. The release of the nanoparticle-encapsulated 800 nm fluorophore was faster under oxidative stress for the respective nanoparticles. **F**) Graphs of the 700 nm fluorescence signal changes along the length of the agarose gel phantom tubes, kept at different conditions of oxidative stress over 15 days (360 h). The high 700 nm fluorescence signal (gray arrow) at the top of the gel (0.0 to 0.1 inches of the gel tube) over the 15-day imaging period indicated no diffusion of the 700 nm fluorescently labelled nanoparticles over time. This was due to the high molecular weights of the nanoparticles. **G**) Graphs of the 800 nm fluorescence signal changes along the length of the agarose gel phantom tubes kept at different conditions of oxidative stress, over 15 days (360 h). The shift in the 800 nm fluorescence signal over the 15-day imaging period indicated diffusion of the released nanoparticle-encapsulated 800 nm fluorophore along the length of the tube over time. Faster release and diffusion rates of the 800 nm fluorophore were detected at pH 6.2 compared to pH 7.4 for both nanoparticles P2a and P2b. Faster release and diffusion rates of the 800 nm fluorophore were also detected at higher H_2_O_2_ concentrations.

**Figure 3:**
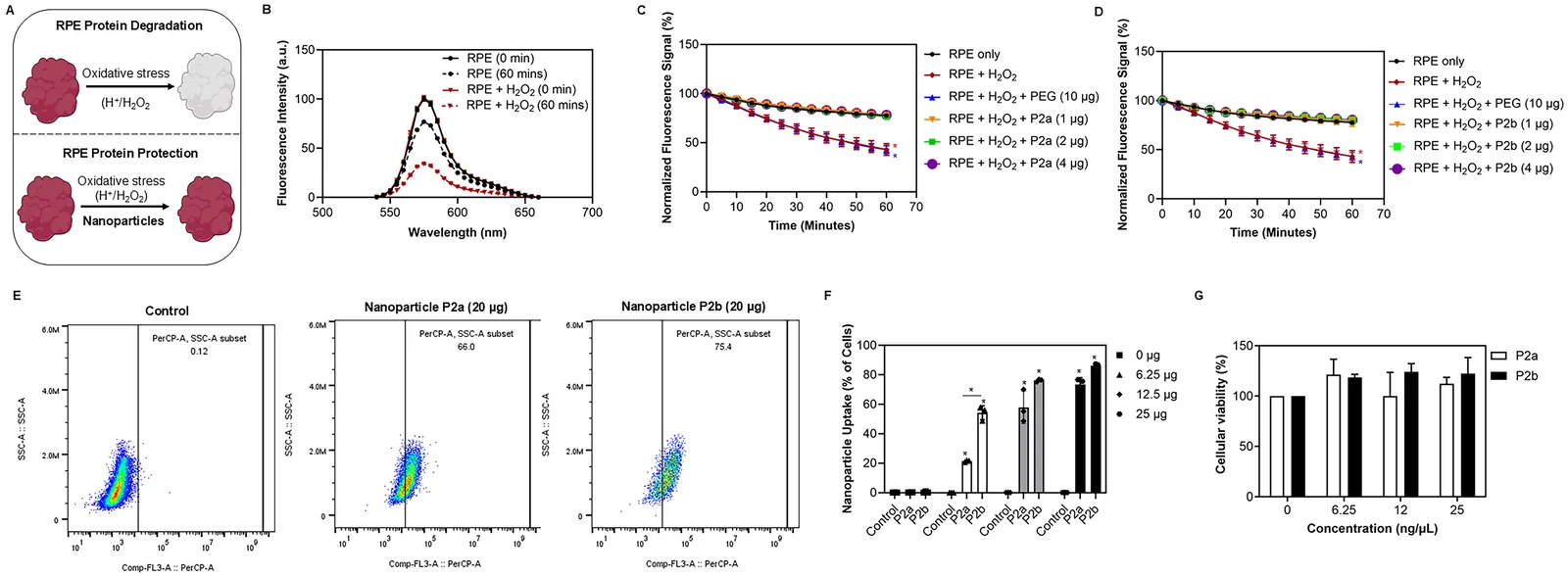
** Protection of fluorescent RPE protein from degradation under oxidative stress by the oxidative stress-activable nanoparticles. A**) Schematic of the degradation of RPE protein under oxidative stress and its protection by the oxidative stress-activable nanoparticles P2a or P2b, under oxidative stress. **B**) Fluorescence spectra of RPE degradation in the absence and presence of oxidative stress (pH 6.2 and 10 mM H_2_O_2_). Kinetics of RPE degradation under oxidative stress and its protection in the presence of nanoparticles P2a (**C**) and P2b (**D**) respectively (*P* < 0.05, n = 3). As little as 1 µg of either nanoparticle P2a (0.034 nmol) or P2b (0.0271 nmol) was sufficient to prevent the degradation of 42.5 nM (0.0085 nmol) of RPE. Increasing the amount of nanoparticles to 2 µg, (0.068 nmol for P2a or 0.0542 nmol for P2b) or 4 µg (0.135 nmol for P2a or 0.108 nmol for P2b) had the same protective effect on the RPE solution. However, the control PEG polymer without ROS scavengers (10 µg, 1.33 nmol) was unable to protect RPE from degradation under oxidative stress. **E**) Flow cytometry images of HUVECs with no nanoparticle, HUVECs with nanoparticle P2a, and HUVECs with nanoparticle P2b, respectively. This showed the cellular uptake of the nanoparticles. **F**) Nanoparticle uptake in HUVECs showed that both nanoparticles were effectively taken up by the cells, with P2b being slightly more taken up than P2a at 6.25 µg/µL (*P* < 0.05, n = 3). **G**) Cell viability of HUVECs, after incubation with up to 20 µg (25 µg/µL) for 24 h, showed no toxicity from either nanoparticle (*P* < 0.05, n = 3).

**Figure 4:**
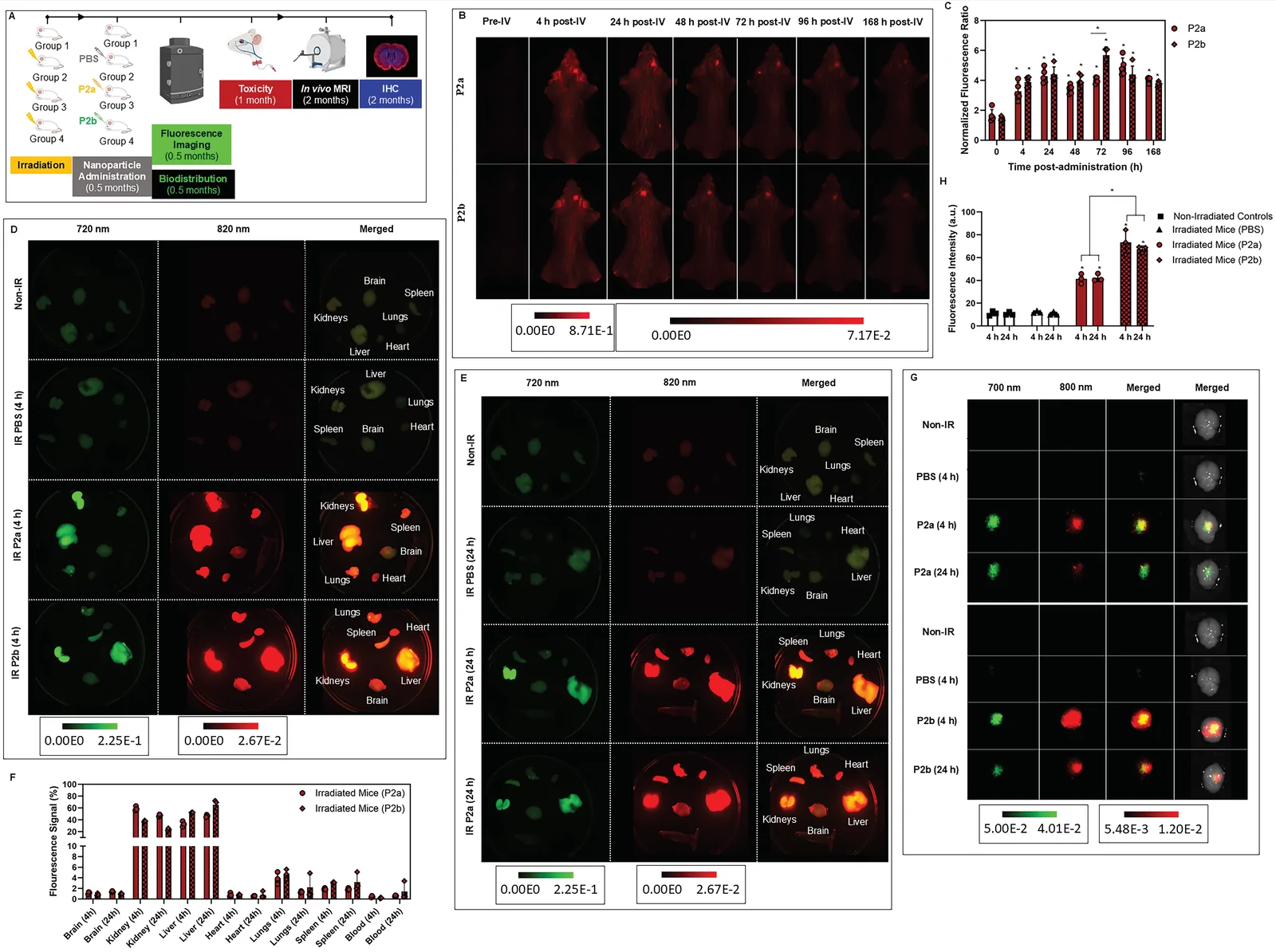
** Fluorescence imaging of nanoparticle pharmacokinetics and biodistribution. A**) Schematic of *in vivo* experimental design. **B**) Fluorescence imaging (820 nm) of nanoparticle pharmacokinetics over a seven-day period after the intravenous administration of the respective nanoparticles P2a and P2b (0.8 mg/Kg) in irradiated mice. This showed the specific accumulation and retention of the respective nanoparticles in the irradiated brain lesions of the mice throughout the seven-day imaging period. **C**) Quantification of the ratio of the 820 nm fluorescence signal from the irradiated brain lesions compared to that from a non-irradiated region of the body over a seven-day period after the intravenous administration of the respective nanoparticles P2a and P2b (0.8 mg/Kg) in irradiated mice (*P* < 0.05, n = 3). This showed statistically significant accumulation and retention of the respective nanoparticles in the irradiated brain lesions of the mice throughout the seven-day imaging period. Fluorescence imaging of nanoparticle biodistribution in irradiated mice at 4 h (**D**) and 24h (**E**) after the intravenous administration (0.8 mg/Kg) of the respective nanoparticles P2a and P2b. **F**) Quantification of the percentage 820 nm fluorescence signals of nanoparticle biodistribution in irradiated mice at 4 h and 24 h after the intravenous administration of the respective nanoparticle P2a and P2b. This showed that approximately 1% of the administered nanoparticles P2a and P2b were detected in the irradiated brain lesions at 4 h and 24 h post-administration. **G**) Comparison of the fluorescence images of the nanoparticle biodistribution in the brains of irradiated mice at 4 h and 24 h after the intravenous administration of the respective nanoparticle P2a and P2b. **H**) Quantification of the 820 nm fluorescence signals of nanoparticle biodistribution in irradiated mouse brains at 4 h and 24 h after intravenous nanoparticle administration (*P* < 0.05, n = 3). This showed an ~ 3-fold higher accumulation of the nanoparticles in the irradiated brain lesions compared to the brains of non-irradiated control mice that received the nanoparticles.

**Figure 5:**
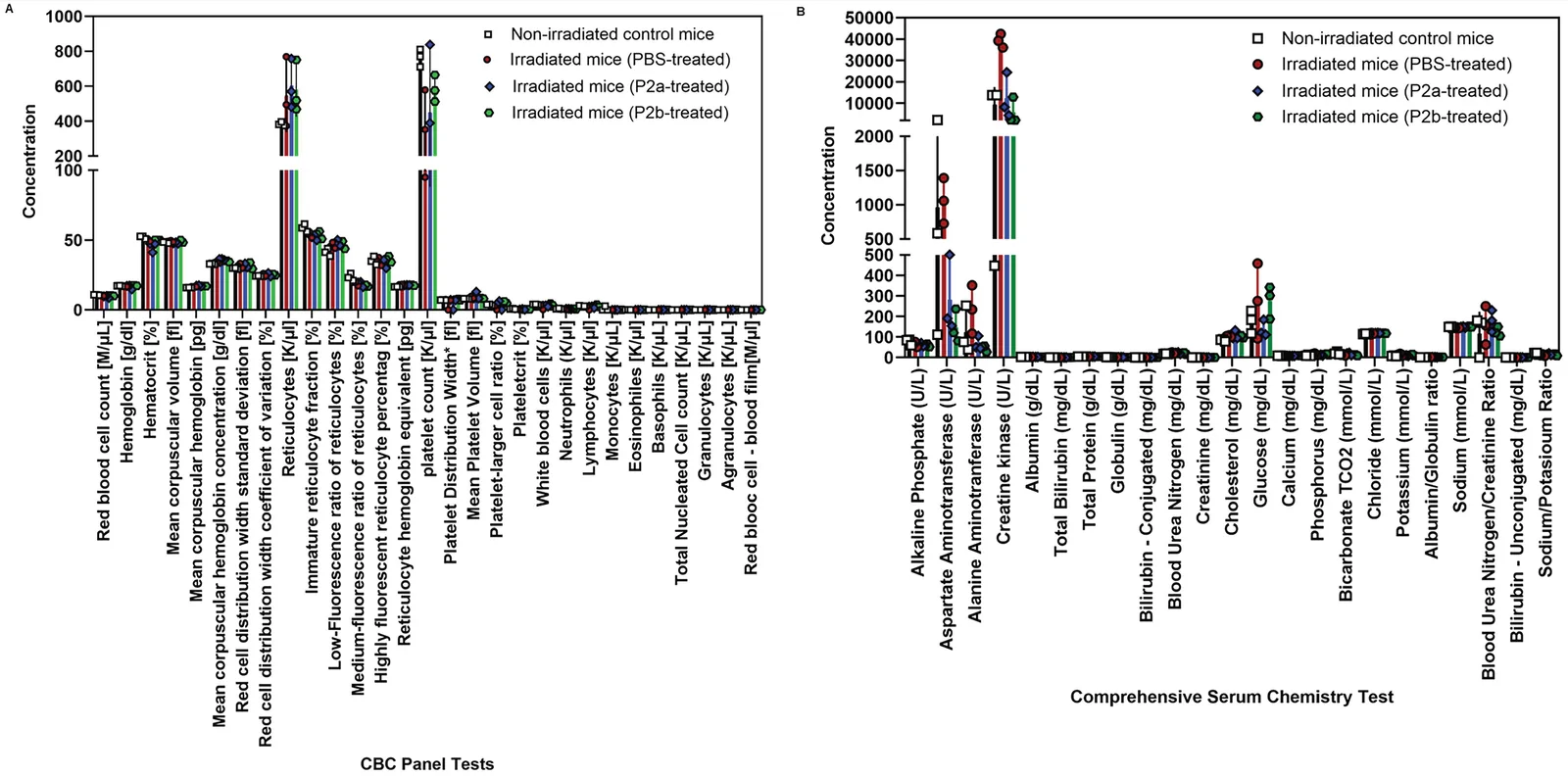
** Nanoparticle toxicity evaluation. A**) Complete blood count analyses conducted 15 days after nanoparticle administration. No statistically significant differences were observed between the different mouse groups.** B**) Comprehensive serum chemistry tests conducted 15 days after nanoparticle administration. No statistically significant differences were observed between the different mouse groups. These confirmed the safety of both nanoparticles P2a or P2b.

**Figure 6:**
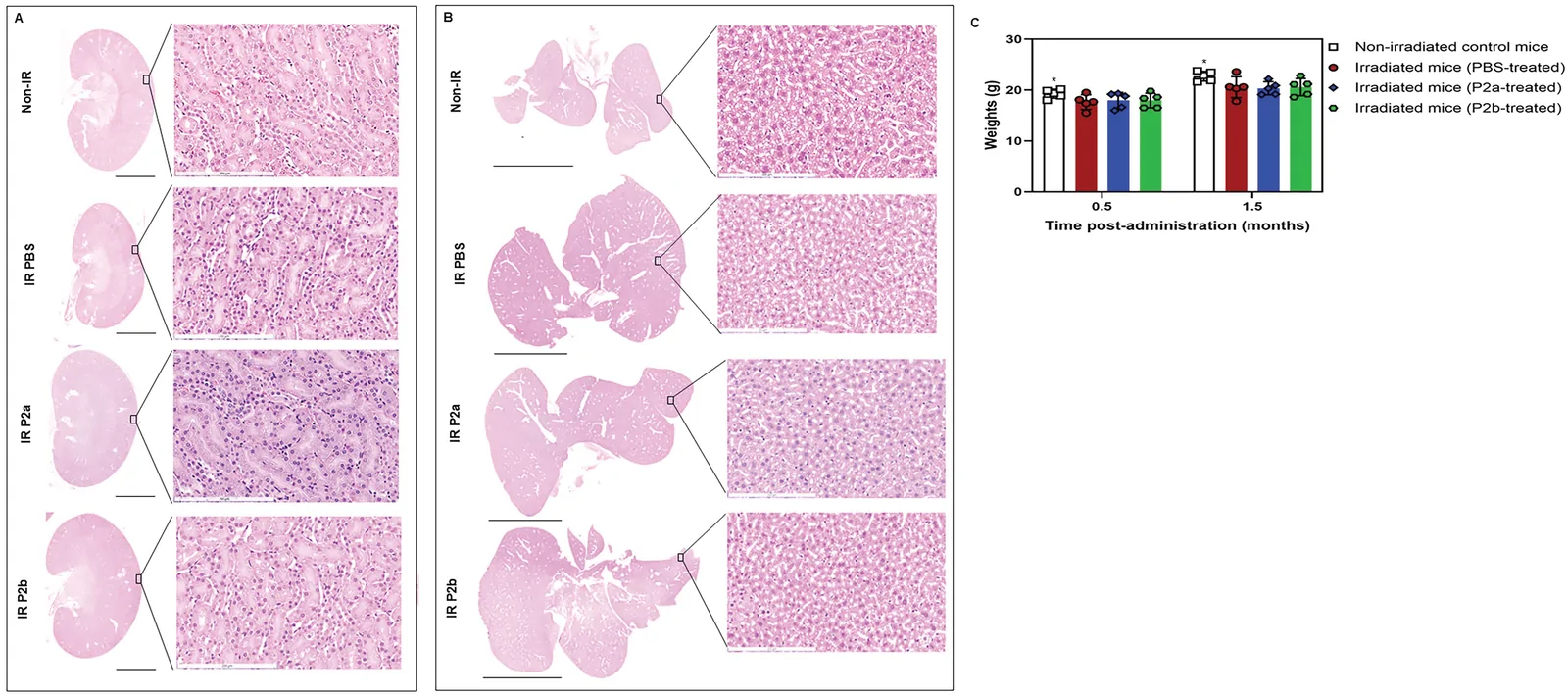
** Evaluation of nanoparticle toxicity by histology. A**) H&E optical images of representative kidneys from the respective mouse groups at 1.5 months after nanoparticle administration (5 mm and 200 µm scale bars respectively). **B**) H&E optical images of representative livers of mice from the respective groups at 1.5 months after nanoparticle administration (10 mm and 200 µm scale bars respectively). No signs of kidney or liver toxicity were detected. These further confirmed the safety of both nanoparticles P2a or P2b. **C**) Weight changes in mice at 0.5 and 1.5 months after nanoparticle administration. No statistically significant differences were detected between the irradiated mice that received PBS versus the irradiated mice that received one of the nanoparticles. This confirmed the safety of both nanoparticles P2a or P2b. However, statistically significant differences (*P* <0.05) were detected between the non-irradiated control mice and the irradiated mice that received either PBS or one of the nanoparticles. This suggested radiation-induced toxicity in the irradiated mice.

**Figure 7:**
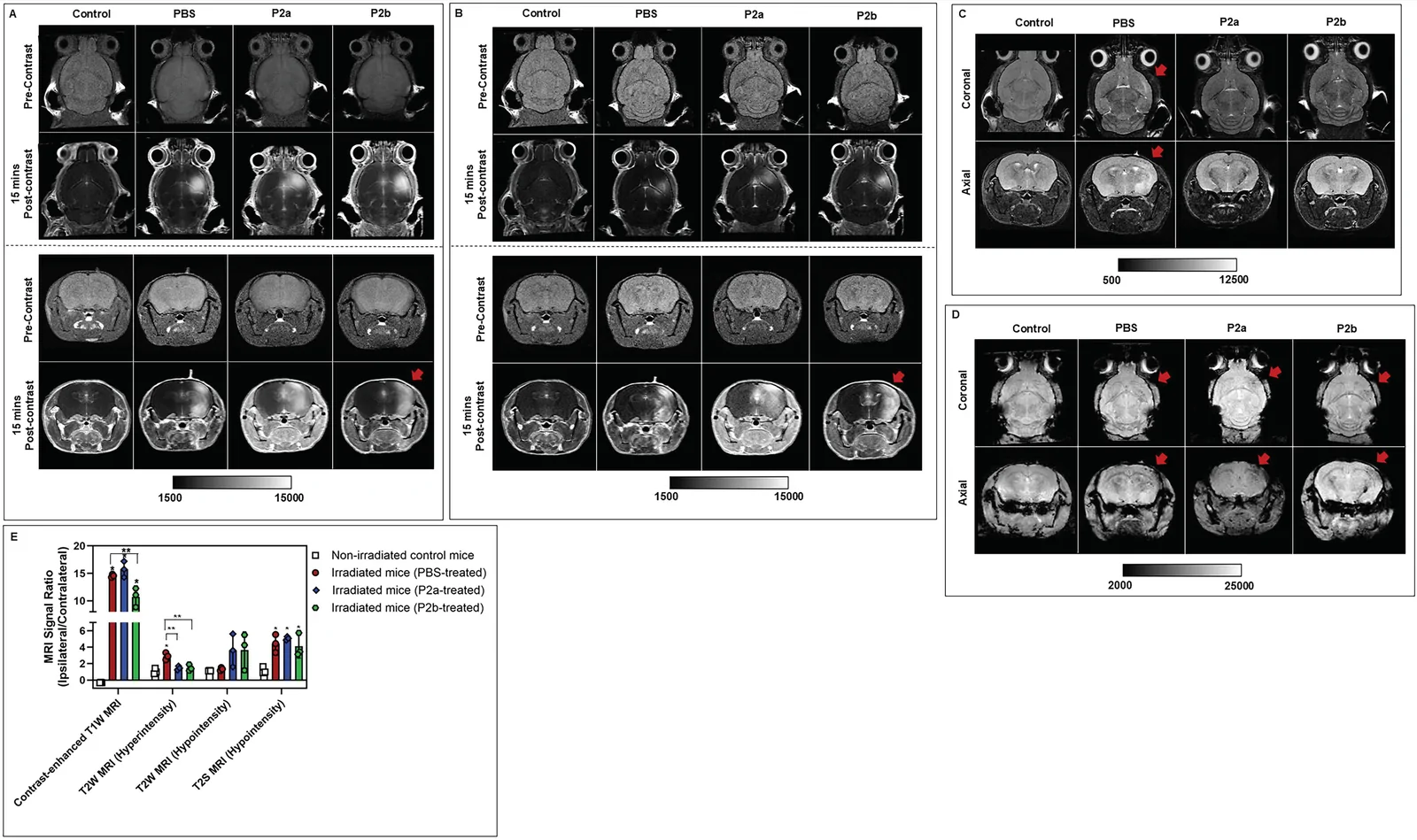
***In vivo* multi-parametric MRI evaluation of nanoparticle efficacy.** Evaluation of changes in BBB permeability, detected using *in vivo* contrast-enhanced T_1_W MRI, at 1.5 months after nanoparticle administration: **A**) Gray-scale coronal and axial views of Z-projections of several slices. **B**) Gray-scale coronal and axial views of single slices. This showed that the degree of BBB permeability was less in the irradiated mice that received nanoparticle P2b (red arrow) compared to the irradiated mice that received either PBS or nanoparticle P2a. **C**) Evaluation of anatomical changes using the respective *in vivo* T_2_W MRI hyperintensity and hypointensity signals at 1.5 months after nanoparticle administration: Gray-scale coronal and axial views of single slices. This showed less hyperintensity (indicative of less edema) in the irradiated mice that received either nanoparticle P2a or P2b than in the irradiated mice that received PBS (red arrow). **D**) Evaluation of hemorrhage changes using the more sensitive *in vivo* T_2_^*^W MRI hypointensity signal at 1.5 months after nanoparticle administration: Gray-scale coronal and axial views of single slices. This showed significant hemorrhage (hypointensity) in all the irradiated mouse groups (red arrow) compared to the non-irradiated mice. This suggested that higher doses of the nanoparticles may be needed to effectively reduce radiation-induced hemorrhage in RIBI. **E**) Quantification of the respective multi-parametric MRI signals in the irradiated brain hemisphere normalized to the non-irradiated contralateral brain hemisphere of the respective mice, at 1.5 months after nanoparticle administration (*P* <0.05, n = 3).

**Figure 8:**
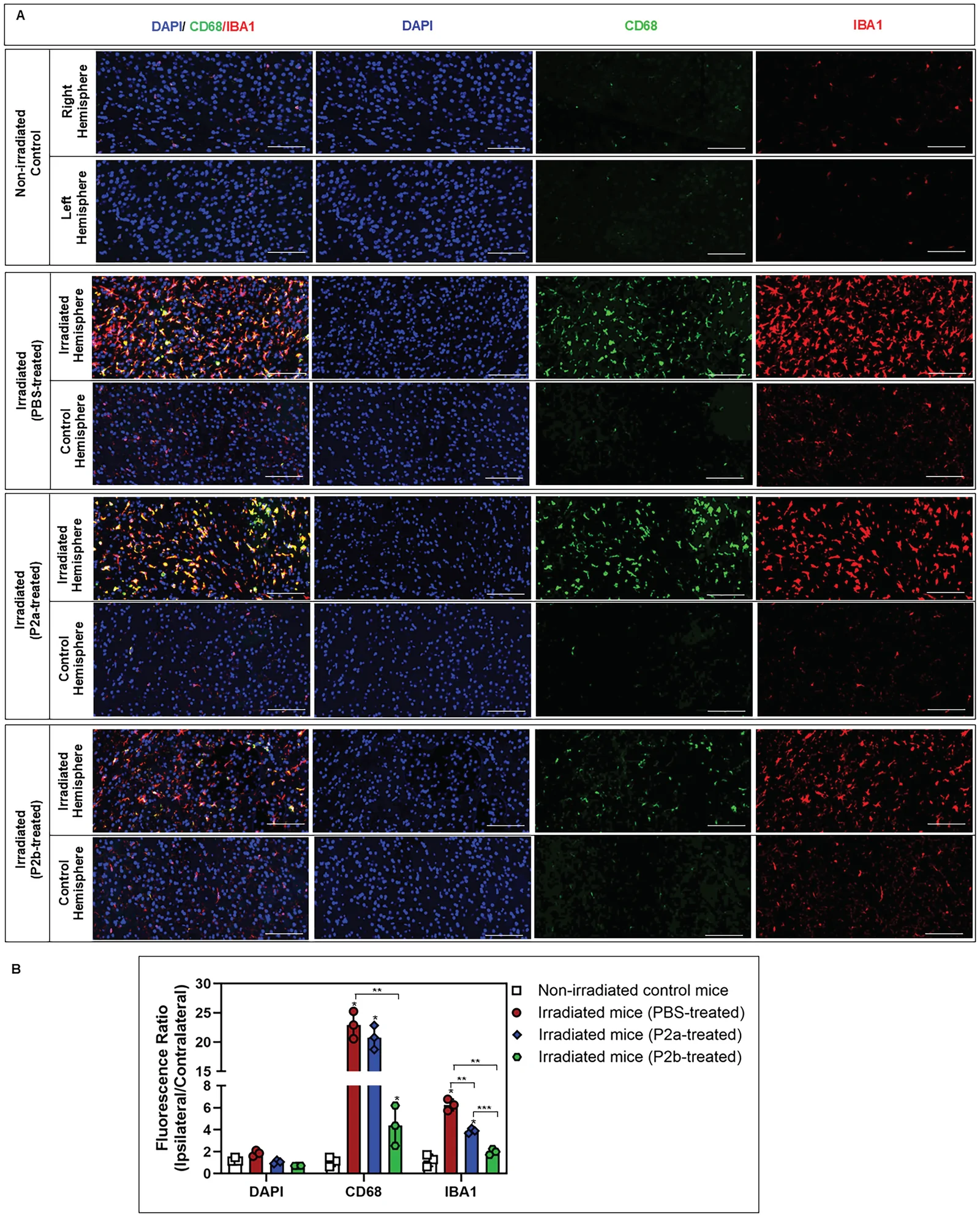
** Evaluation of nanoparticle mitigation of neuroinflammation using CD68 (green) and IBA1 (red) immunohistochemistry. A**) Fluorescence images of representative mouse brain regions from the respective study groups (200 µm scale bar). **B**) Quantification of the respective CD68 (Green) and IBA1 (red) fluorescence signals in the irradiated brain hemisphere normalized to the non-irradiated contralateral brain hemisphere of the respective mice, at 1.5 months after nanoparticle administration. A statistically significant difference in CD68 staining was detected between the irradiated mice that received nanoparticle P2b and the irradiated mice that received either PBS (*P* = 0.0004, n = 3) or nanoparticle P2a (*P* =0.0005, n = 3). Statistically significant differences in IBA1 staining were detected between the irradiated mice that received either nanoparticles P2a (*P* = 0.0018, n = 3) or P2b (*P* = 0.0002, n = 3) and the irradiated mice that received PBS. Additionally, the irradiated mice that received nanoparticle P2b had significantly less neuroinflammation (IBA1 staining) than the irradiated mice that received nanoparticles P2a (*P* =0.0007, n = 3). This suggested that nanoparticle P2b was more effective at controlling neuroinflammation than nanoparticle P2a.

**Figure 9:**
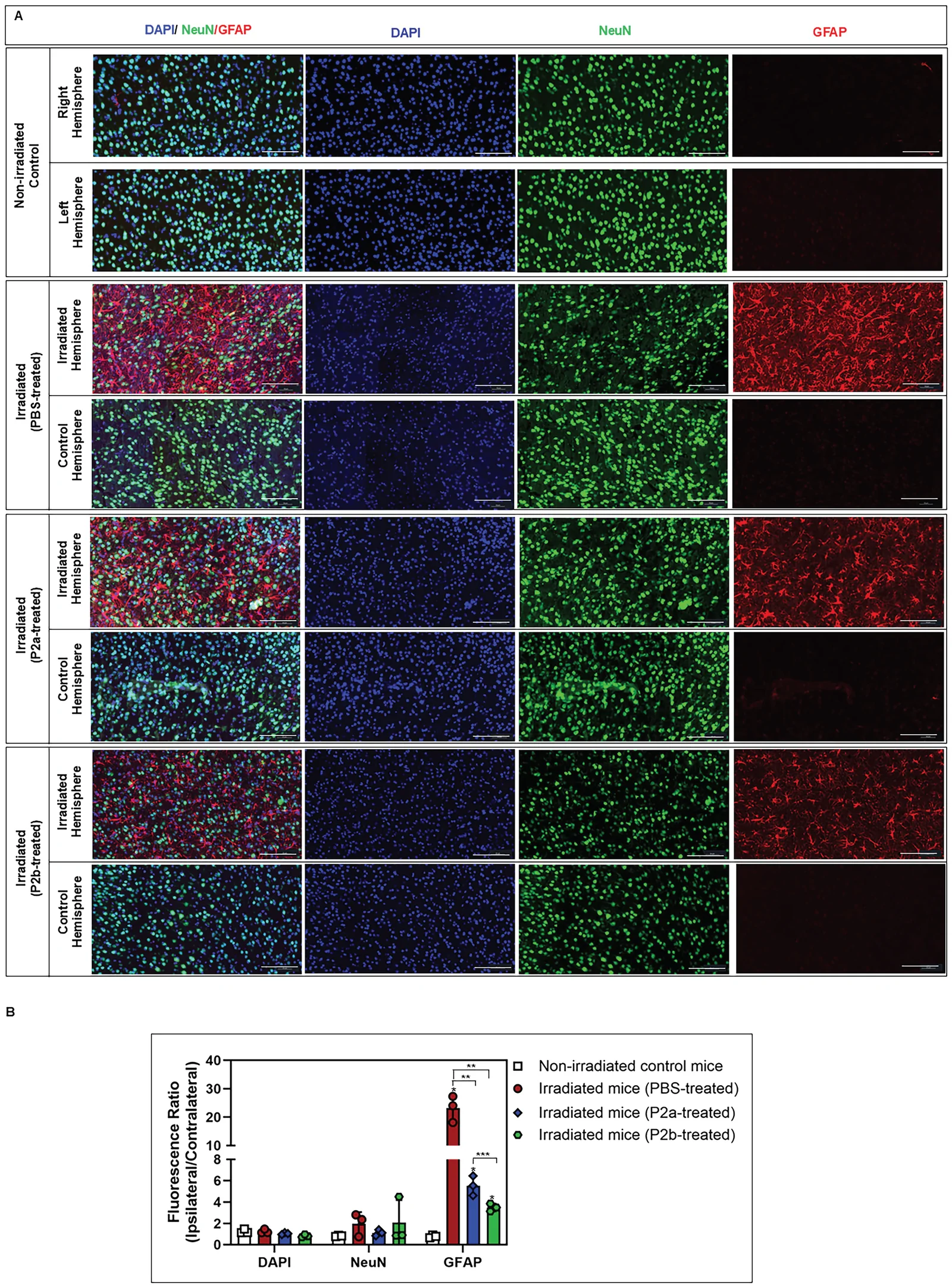
** Evaluation of nanoparticle mitigation of neuronal cell loss and astrogliosis using NeuN (green) and GFAP (red) immunohistochemistry. A**) Fluorescence images of representative mouse brain regions from the respective study groups (200 µm scale bar). **B**) Quantification of the respective NeuN (Green) and GFAP (red) fluorescence signals in the irradiated brain hemisphere normalized to the non-irradiated contralateral brain hemisphere of the respective mice, at 1.5 months after nanoparticle administration. No statistically significant differences in NeuN staining were detected between any of the mouse groups at this time point. Statistically significant differences in GFAP staining were detected between the irradiated mice that received either nanoparticles P2a or P2b and the irradiated mice that received PBS (*P* <0.05, n = 3). This suggested the effectiveness of both nanotheranostic agents at controlling astrogliosis. Additionally, the irradiated mice that received nanoparticle P2b had significantly less astrogliosis (GFAP) than the irradiated mice that received nanoparticles P2a (*P* =0.0230, n = 3). This suggested that nanoparticle P2b was more effective at controlling astrogliosis than nanoparticle P2a.

**Table 1:** Hydrodynamic diameters, poly dispersity indices (PDIs), and zeta potentials of nanoparticles

	Hydrodynamic diameters (nm)		Poly dispersity indices		Zeta potentials (mV)	
Nanoparticles	pH 7.4	pH 6.2 & 0.5 mM H_2_O_2_	PDI (pH 7.4)	PDI (pH 6.2 & 0.5 mMH_2_O_2_)	pH 7.4	pH 6.2 & 0.5 mM H_2_O_2_
P2a	166 ± 51	30.7 ± 22.63	0.073 ± 0.02	0.92 ± 0.11	**-** 2.52 ± 0.66	**+** 4.82 ± 3.01
P2b	183 ± 54	25.62 ± 3.57	0.087 ± 0.00	1.00 ± 0.00	**-** 2.89 ± 1.58	**+** 2.99 ± 0.97

## Data Availability

All the datasets and data processing methods used in this study are available for sharing upon the receipt of reasonable requests made to the corresponding author.
