# Supplementary material for: Developing oxidative stress-responsive neuroprotective nanotheranostic agents for the image-guided treatment of radiotherapy-induced brain injury
Source: Nanotheranostics. 2026 Sep 24;10:224–52. doi: 10.7150/ntno.138059 (PMC13619439; doi:10.7150/ntno.138059)
Supplement: Supplementary file 1 [file ntnov10p0224s1.pdf]

Supplemental Digital Content 1

Synthetic Scheme S1 .... **2**
Synthetic Scheme S2 .... **2**
Synthetic Scheme S3 .... **3**
Synthetic Scheme S4 .... **3**
Synthetic Scheme S5 .... **4**
Synthetic Scheme S6 .... **4**
Synthetic Scheme S7 .... **5**
Synthetic Scheme S8 .... **5**

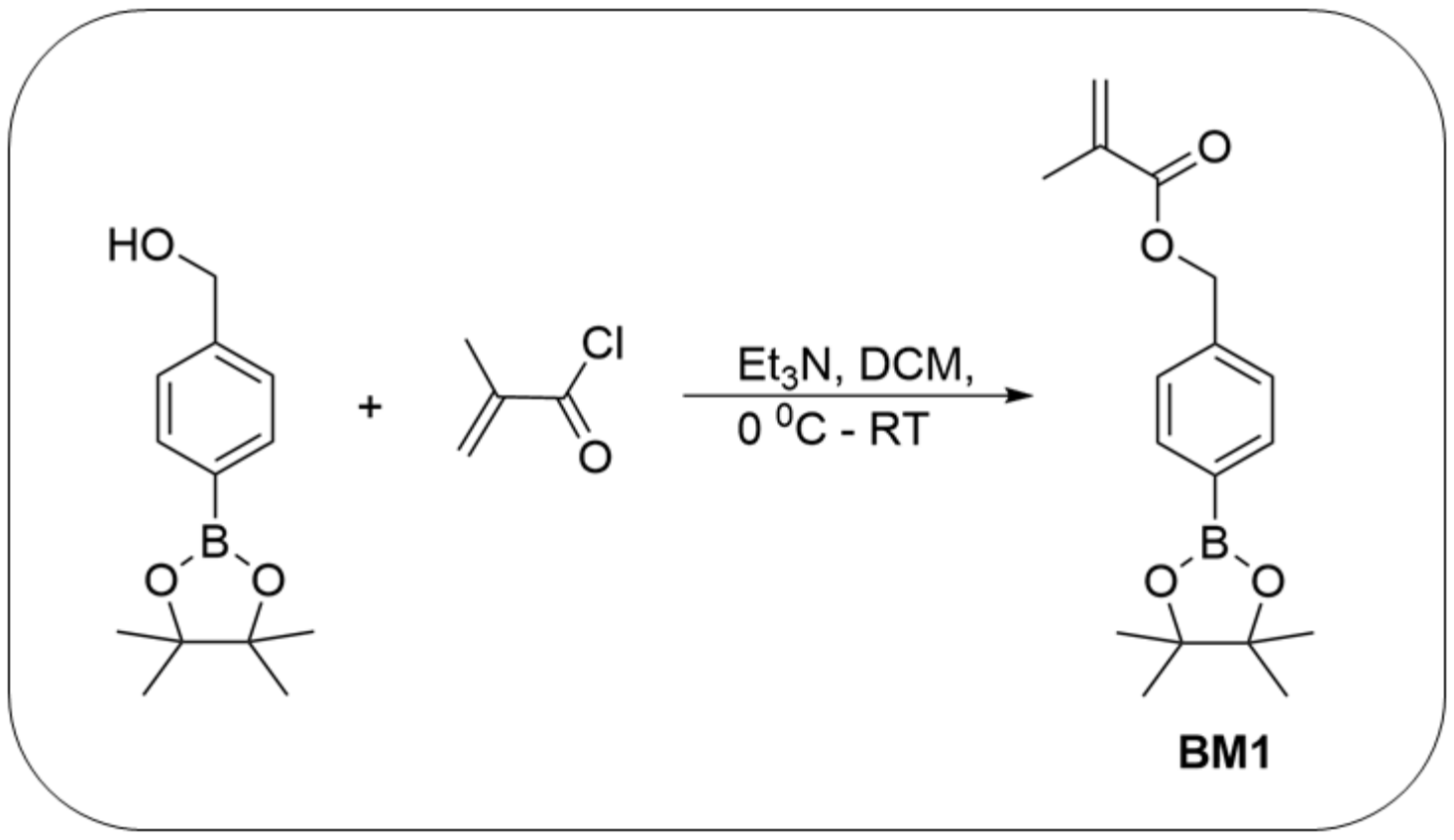

Supplemental Scheme S1. *Reactants:* 4-(hydroxymethyl)phenylboronic acid pinacol ester (0.011 mol) and methacryloyl chloride (0.013 mol). *Reagents:* 0.016 mol of triethylamine (Et$_3$N) and 60 mL of dichloromethane (DCM).

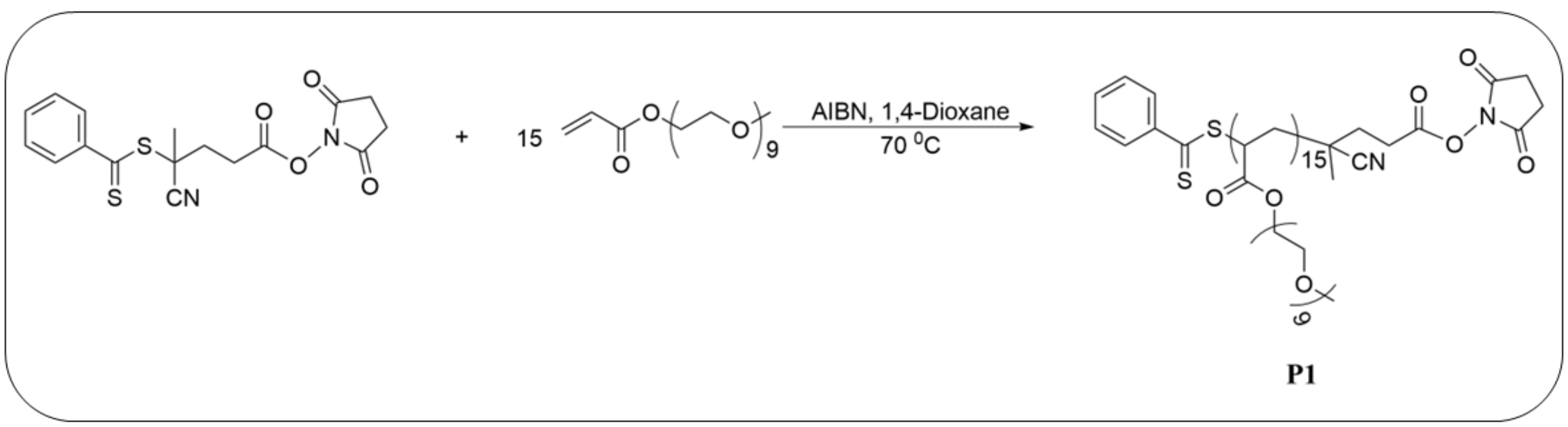

Supplemental Scheme S2. *Reactants*: 4-Cyano-4(phenylcarbonothioylthio)pentanoic acid N-succinimidyl ester (0.142 mmol) and poly(ethylene glycol) methyl ether acrylate (2.13 mmol). *Reagents:* 0.028 mmol of azobisisobutyronitrile (AIBN) and 3 mL of 1,4-dioxane.

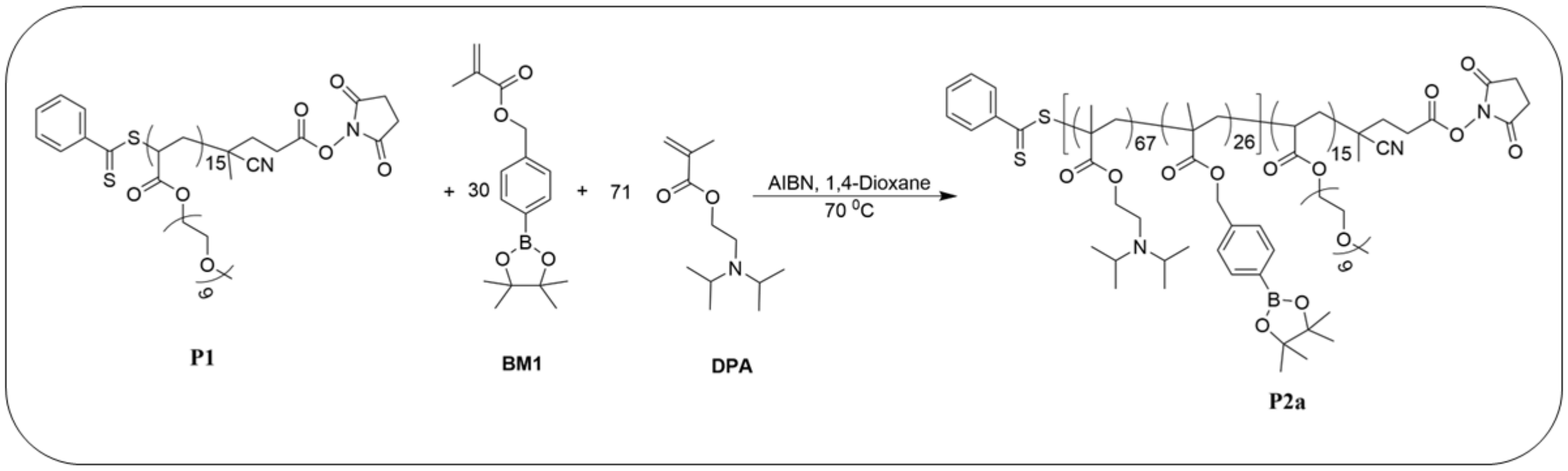

Supplemental Scheme S3. *Reactants*: P(PEGMEA) macro-CTA (P1) (0.0267 mmol), 2-(diisopropylamino)ethyl methacrylate (DPA) (1.9 mmol), and monomer BM1 (0.8 mmol). *Reagents:* 0.007 mmol of AIBN and 4 mL of 1,4-dioxane.

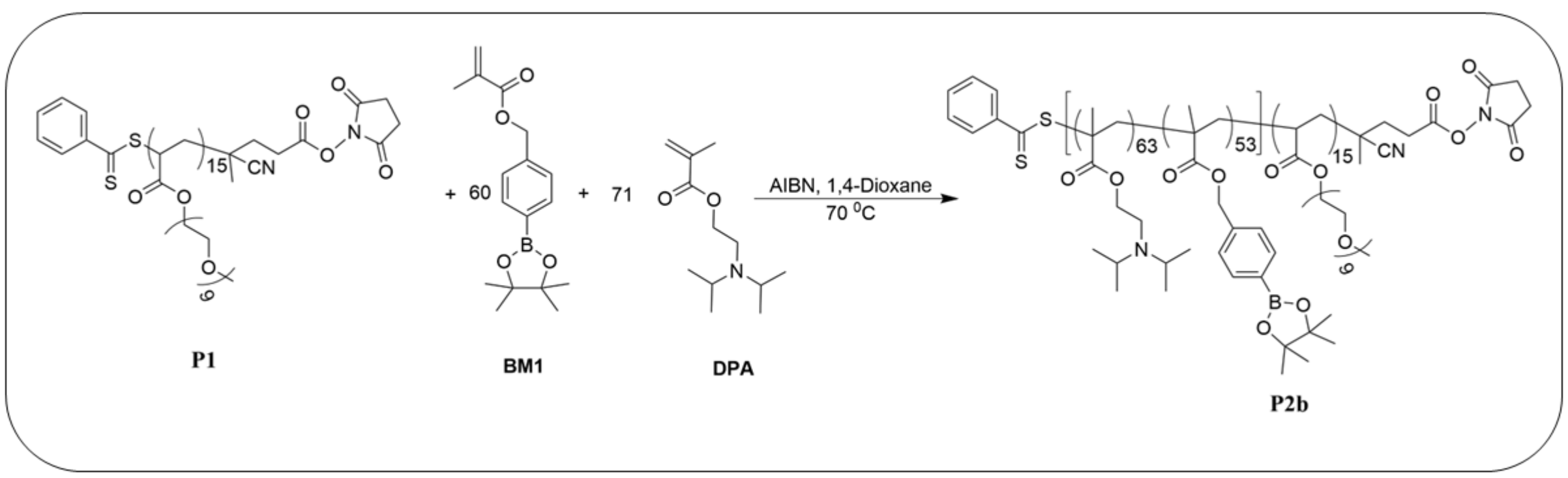

Supplemental Scheme S4. *Reactants*: P1 (0.0267 mmol), DPA (1.9 mmol), and monomer BM1 (1.6 mmol). *Reagents:* 0.007 mmol of AIBN and 4 mL of 1,4-dioxane.

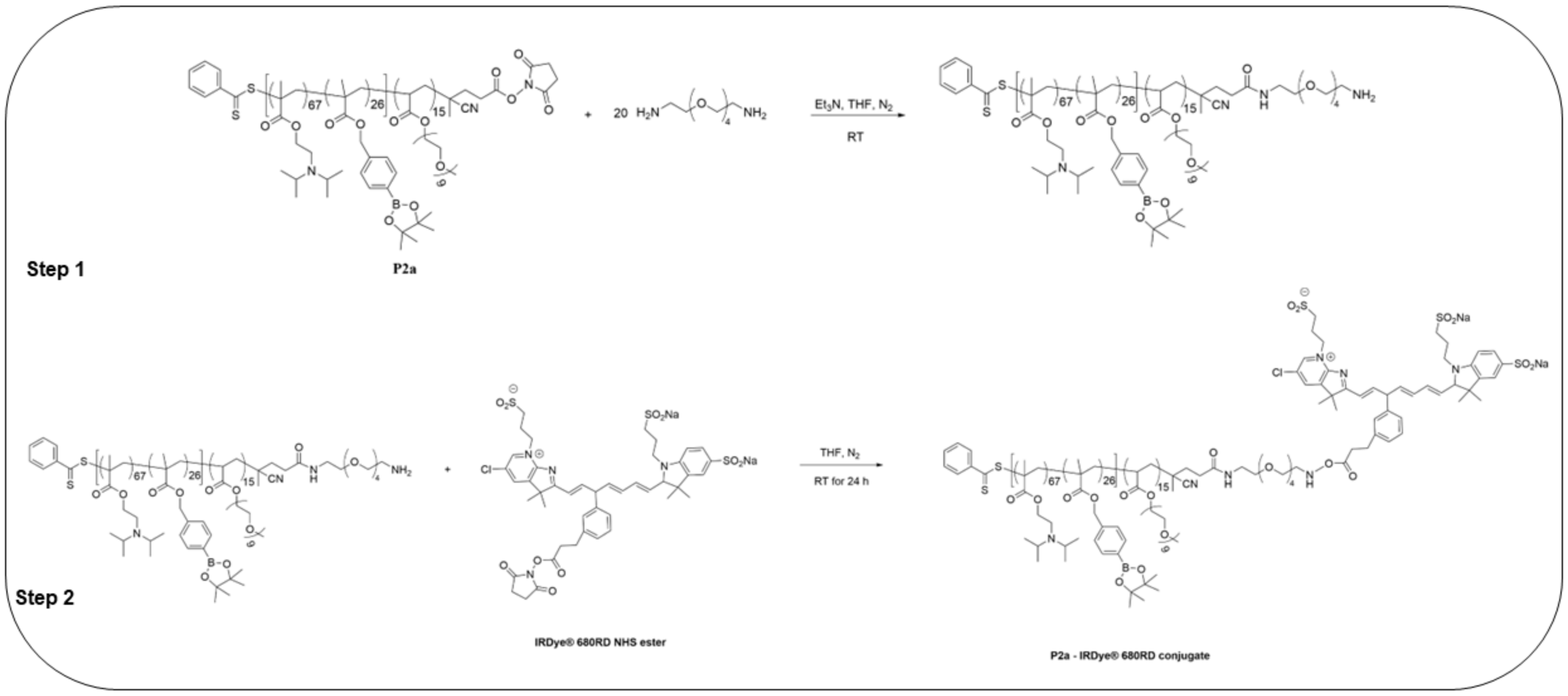

Supplemental Scheme S5. *Reactants*: P2a (0.27 µmol), 1,14-diamino-3,6,9,12-tetraoxatetradecane (5.4 µmol), and IRDye® 680RD NHS ester (0.324 µmol). *Reagents:* 6.75 µmol of Et_3N and 4 mL of tetrahydrofuran (THF), under inert atmosphere.

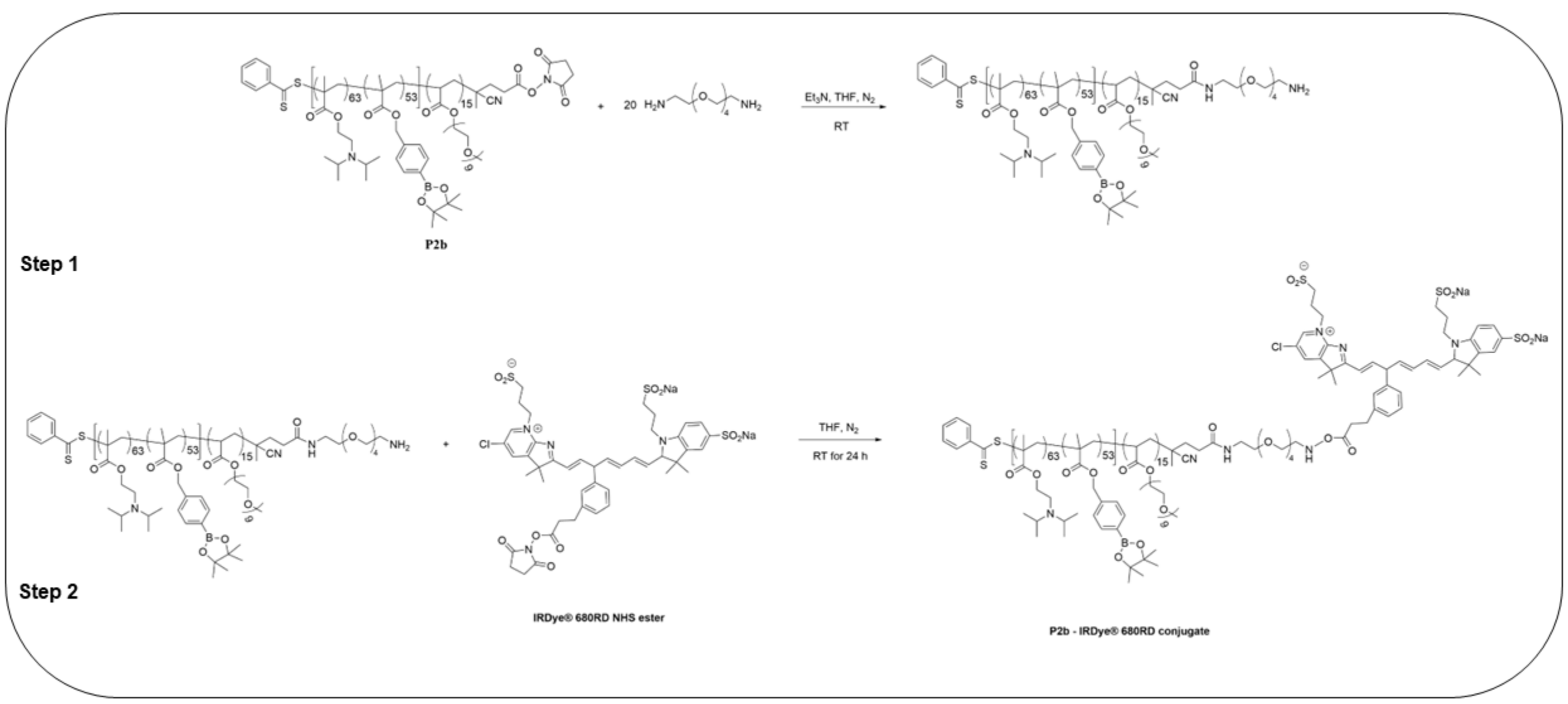

Supplemental Scheme S6. *Reactants*: P2b (0.22 µmol), 1,14-diamino-3,6,9,12-tetraoxatetradecane (4.4 µmol), and IRDye® 680RD NHS ester (0.26 µmol). *Reagents:* 5.5 µmol of Et_3N and 4 mL of THF, under inert atmosphere.

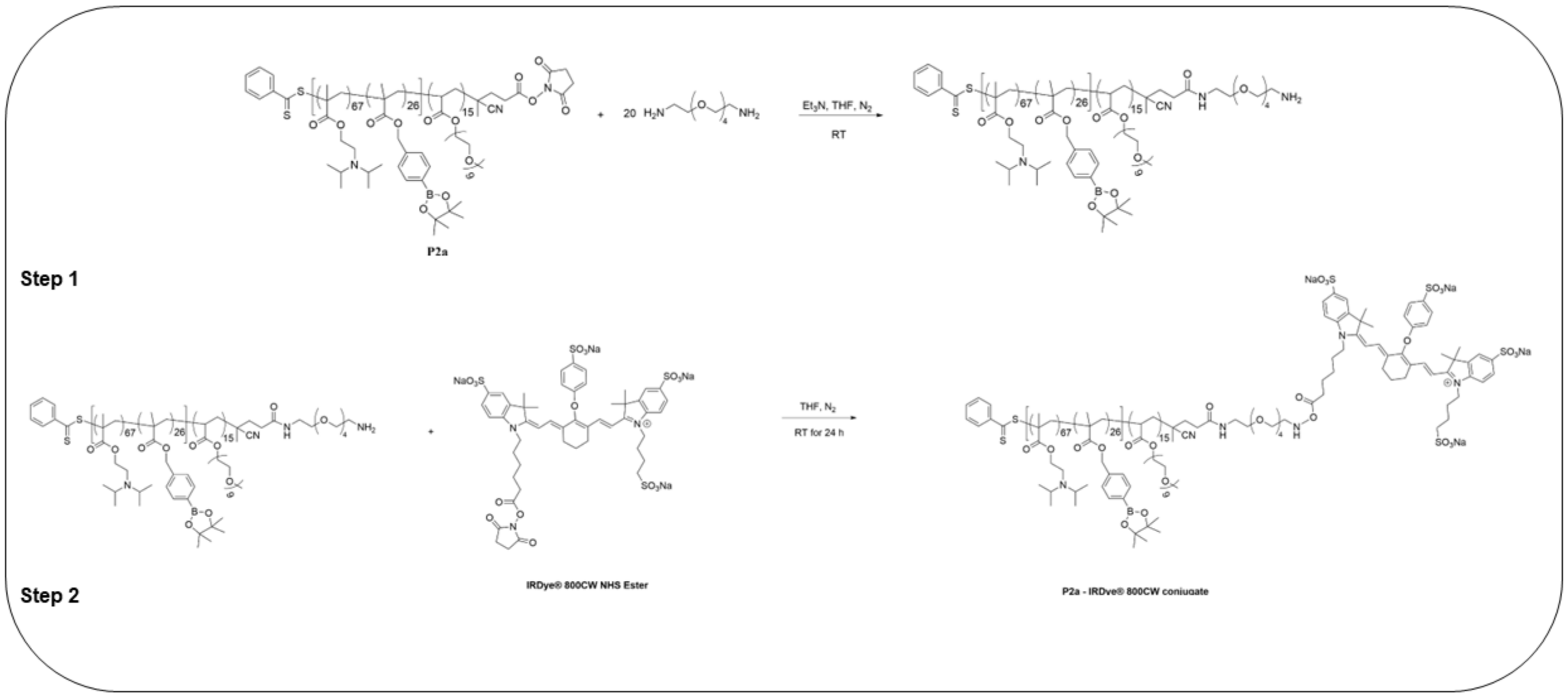

Supplemental Scheme S7. *Reactants*: P2a (0.27 µmol), 1,14-diamino-3,6,9,12-tetraoxatetradecane (5.4 µmol), and IRDye® 800CW NHS ester (0.324 µmol). *Reagents:* 6.75 µmol of Et_3N and 4 mL of THF, under inert atmosphere.

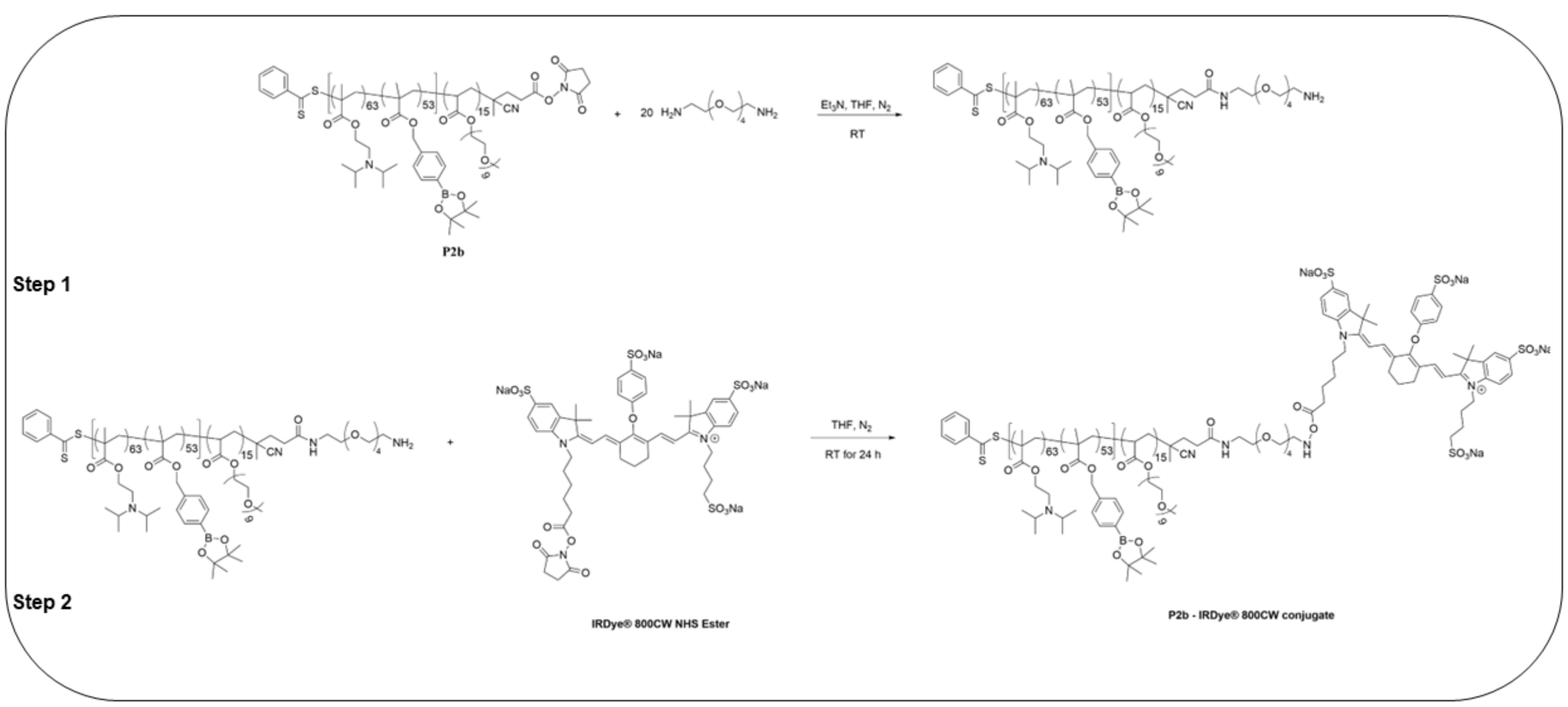

Supplemental Scheme S8. *Reactants*: P2b (0.22 µmol), 1,14-diamino-3,6,9,12-tetraoxatetradecane (4.4 µmol), and IRDye® 800CW NHS ester (0.26 µmol). *Reagents:* 5.5 µmol of Et_3N and 4 mL of THF, under inert atmosphere.

Supplemental Digital Content 2

Figure S1 2
Figure S2 3
Figure S3 4
Figure S4 5
Figure S5 6
Figure S6 7
Figure S7 7
Figure S8 8
Figure S9 8
Figure S10 9
Figure S11 11
Figure S12 12
Figure S13 13
Figure S14 13
Figure S15 14
Figure S16 15
Figure S17 16
Figure S18 17
Figure S19 18
Figure S20 18
Figure S21 19
Figure S22 21

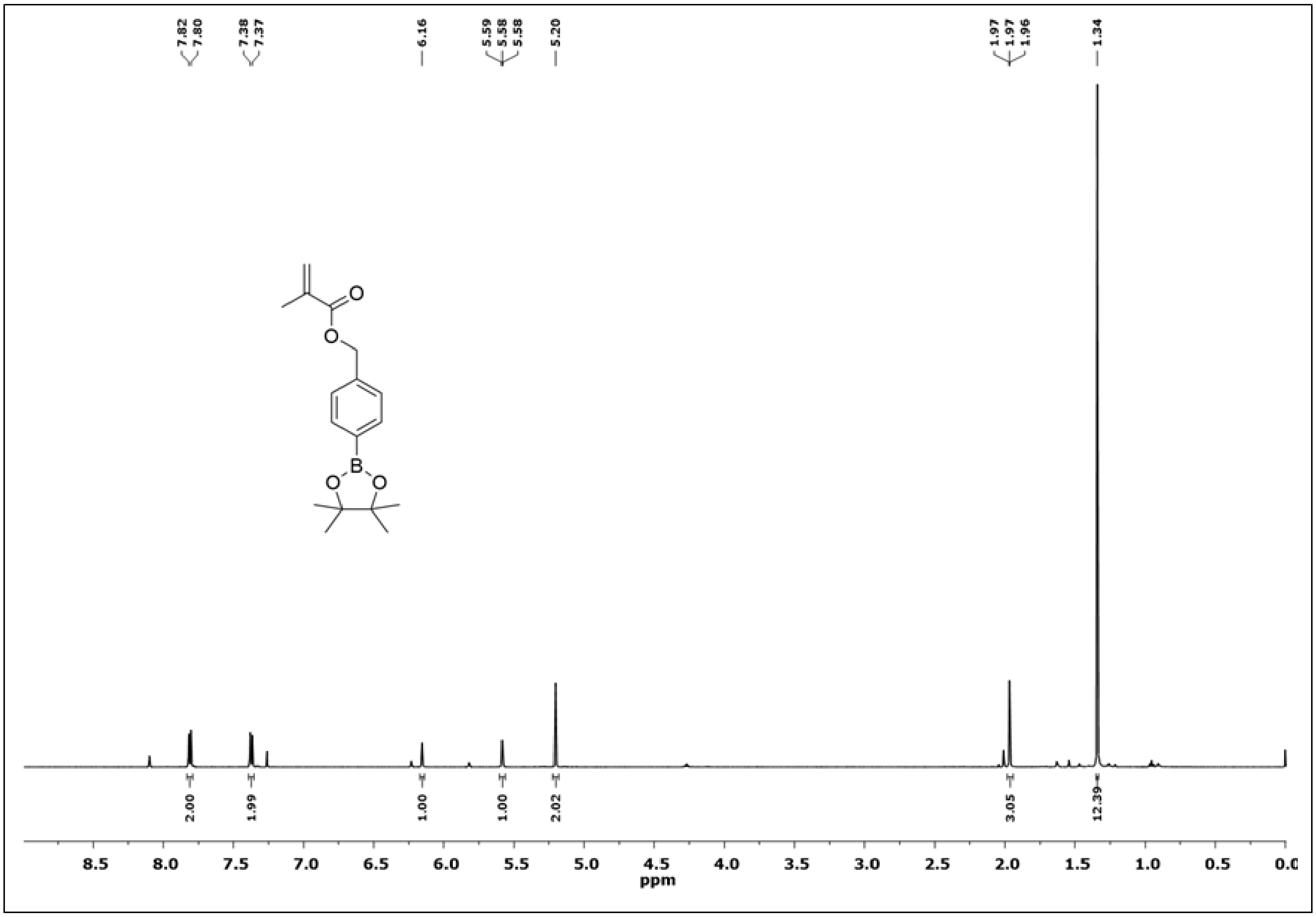

Supplemental Figure S1: ^{1}H NMR spectrum (in $CDCl_3$) of BM1.

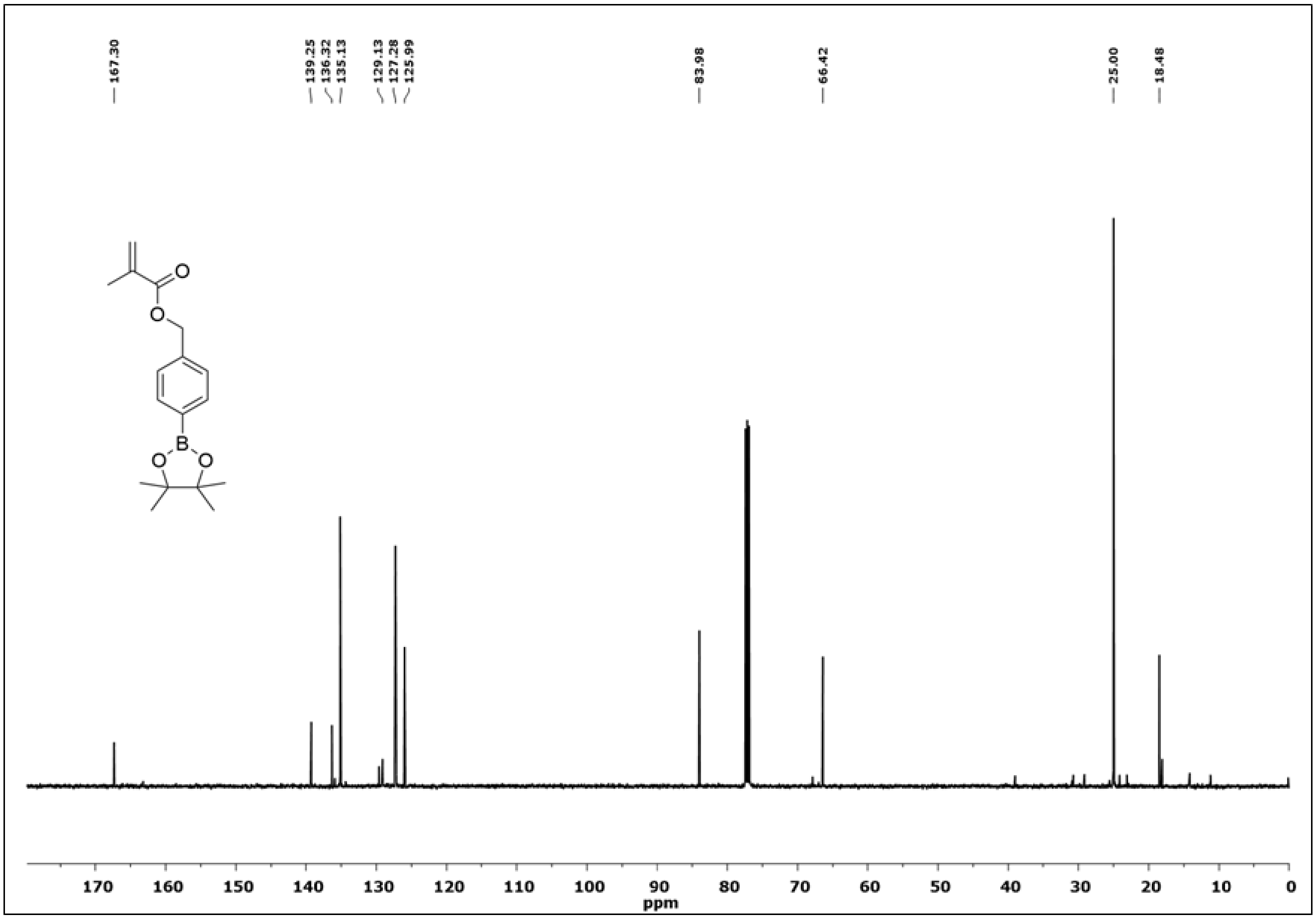

Supplemental Figure S2: ^{13}C NMR spectrum (in $CDCl_3$) of BM1.

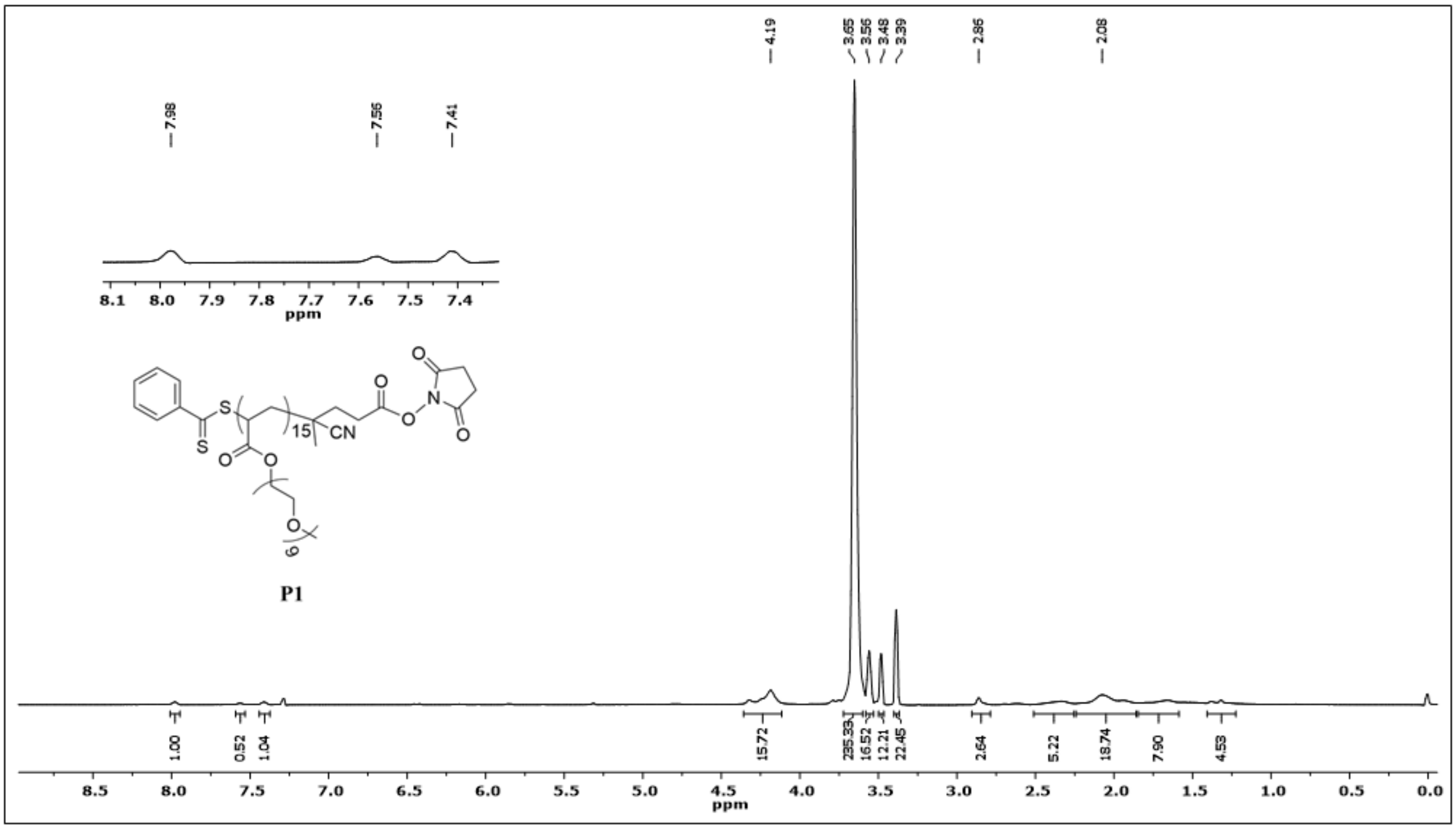

Supplemental Figure S3: ^{1}H NMR spectrum of **P1** (P(PEGMEA$_{480}$) macro-CTA) in $CDCl_3$.

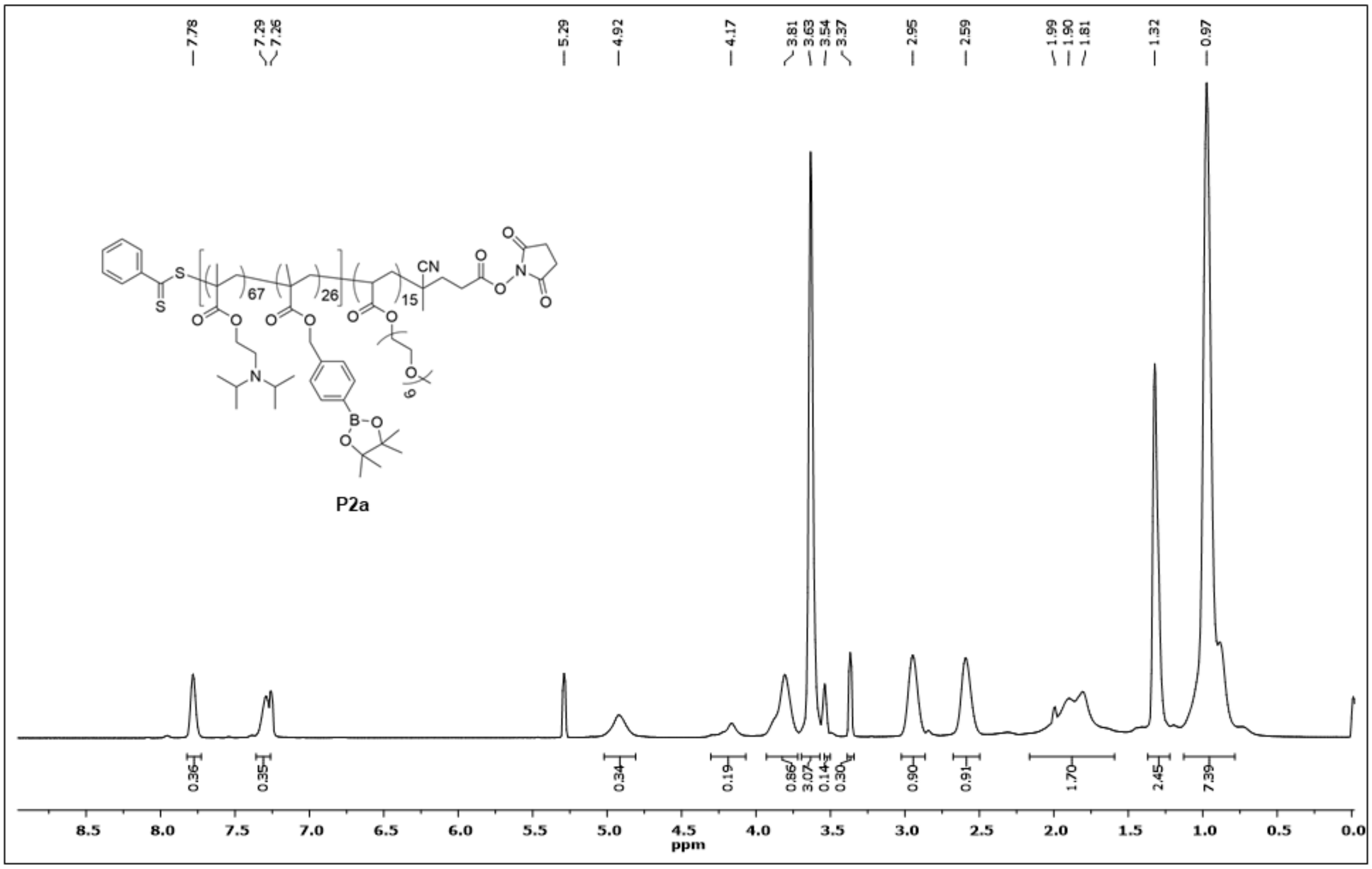

Supplemental Figure S4: ^{1}H NMR spectrum of block co-polymer P2a in $CDCl_3$.

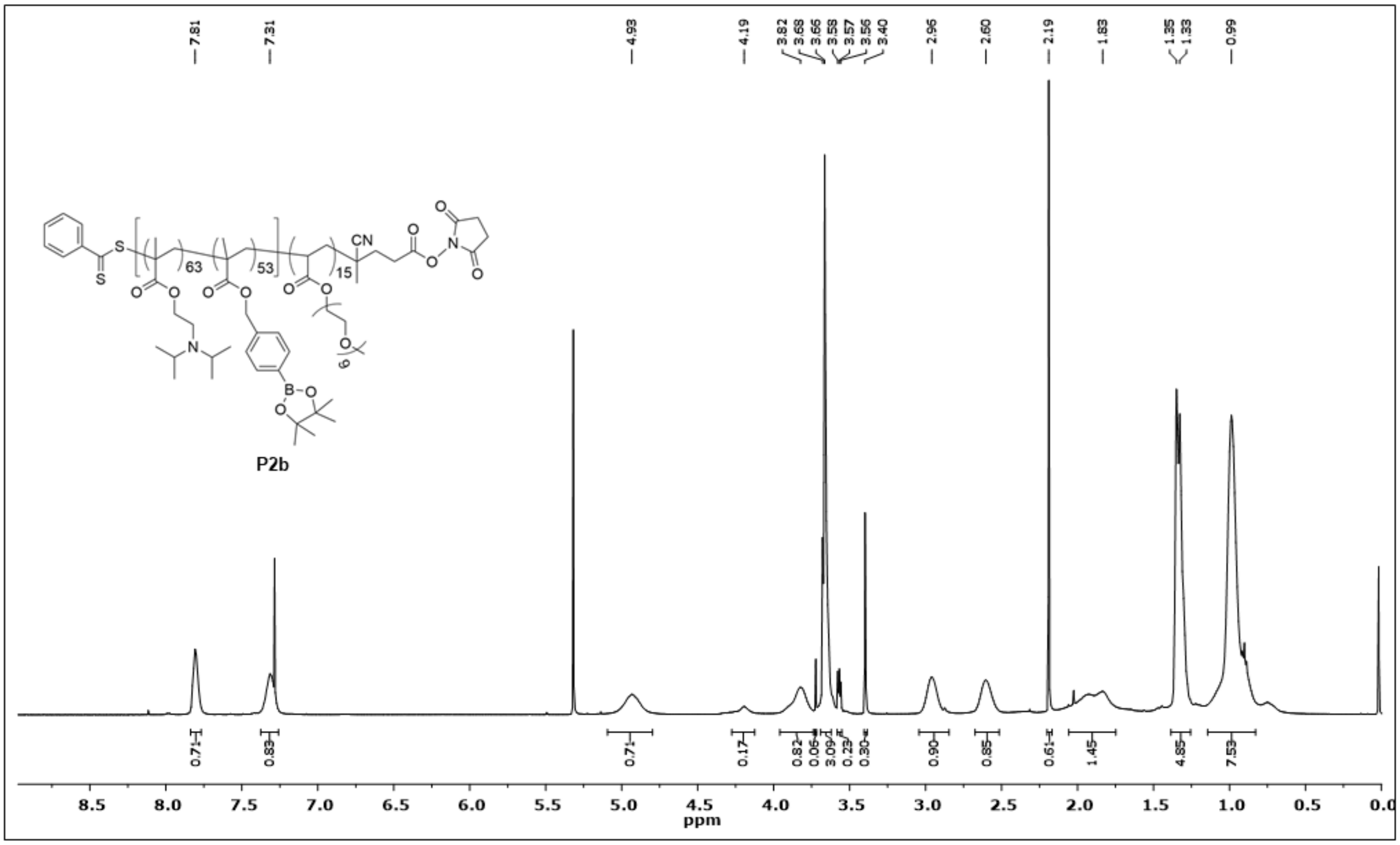

Supplemental Figure S5: ^{1}H NMR spectrum of block co-polymer P2b in $CDCl_3$.

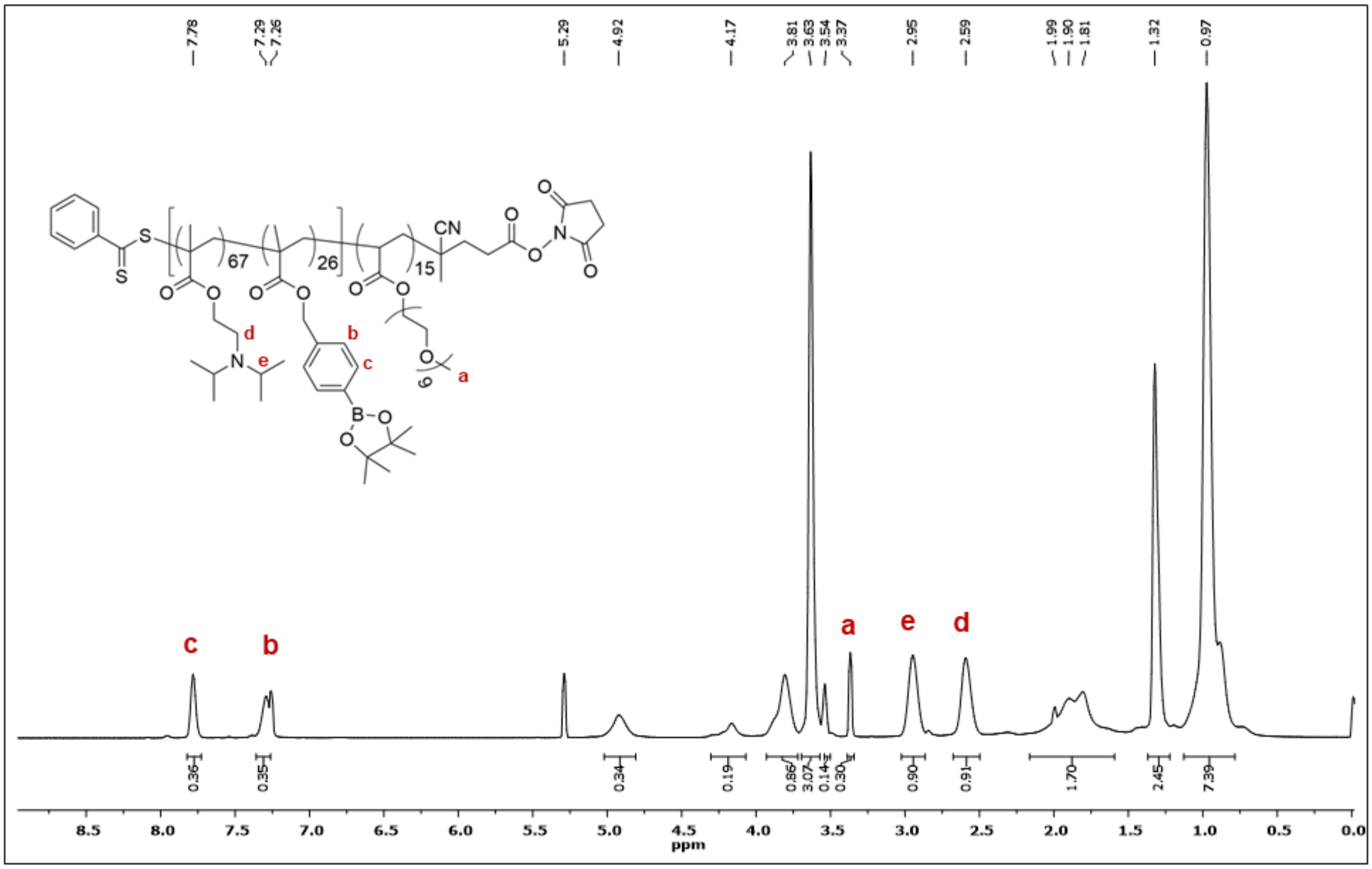

Supplemental Figure S6: Description of ^{1}H NMR spectral characterization of block co-polymer P2a in $CDCl_3$.

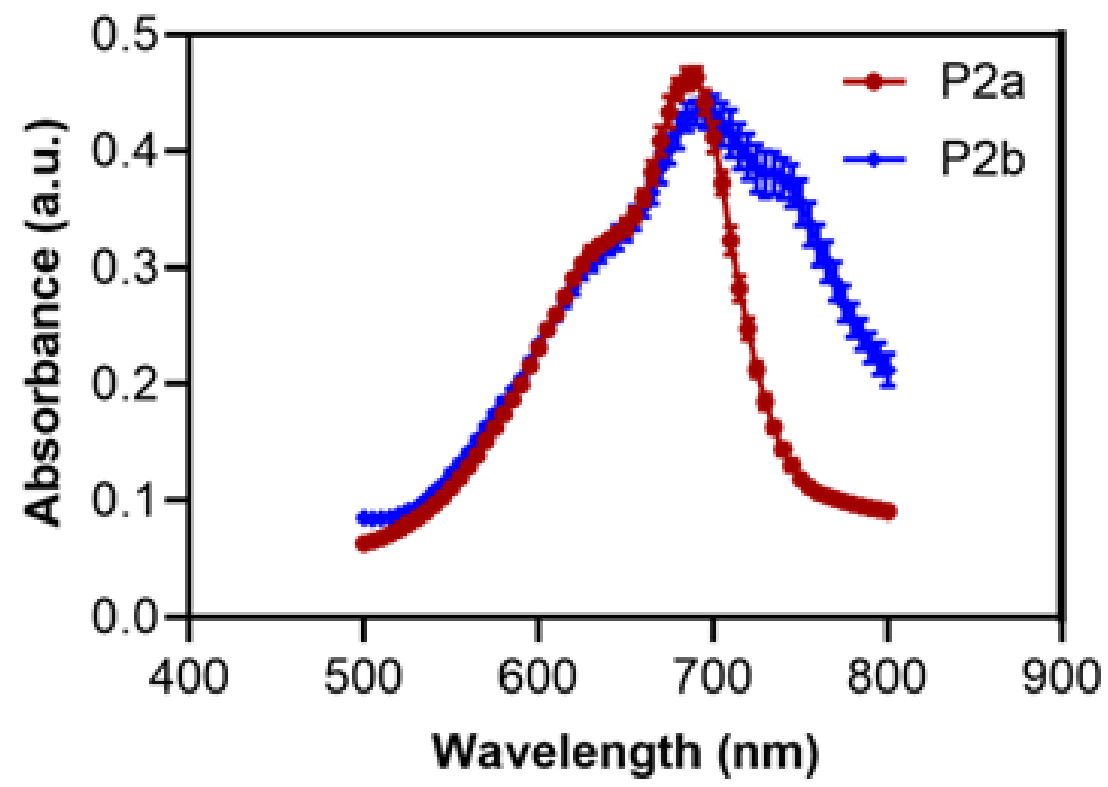

Supplemental Figure S7. Absorbance spectra of the respective polymers P2a and P2b in tetrahydrofuran (2 mg/mL) after conjugation to IRDye® 680RD.

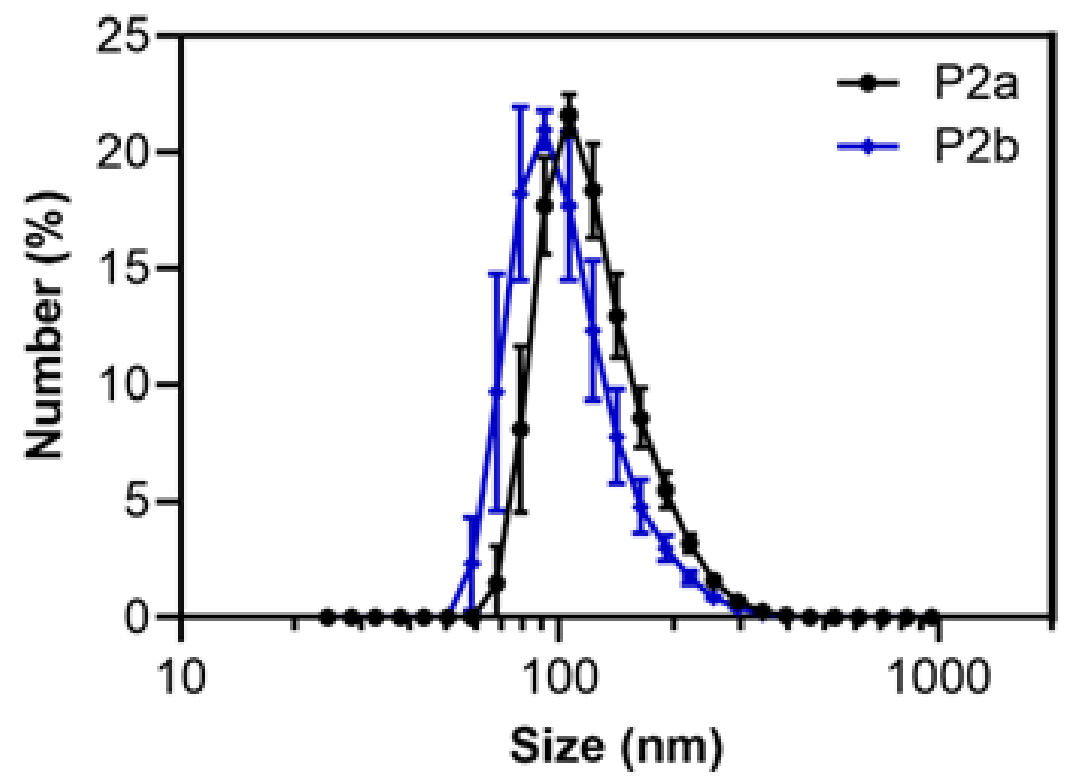

Supplemental Figure S8. Determination of the hydrodynamic diameters of the IRDye® 680RD-conjugated and IRDye® 800CW-encapsulated polymeric nanoparticles P2a and P2b by dynamic light scattering (DLS) at pH of 7.4.

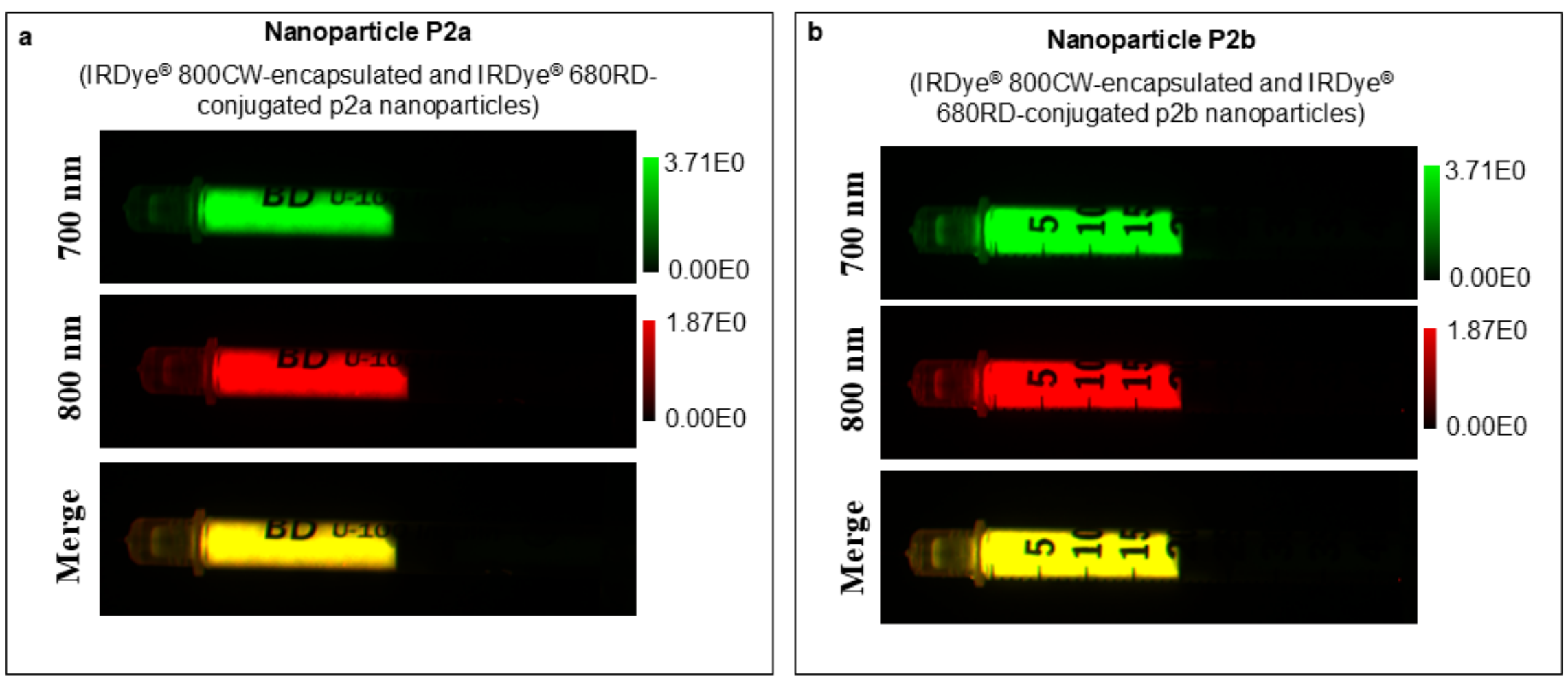

Supplemental Figure S9. Fluorescence images of IRDye® 680RD-conjugated and IRDye® 800CW-encapsulated nanoparticle P2a (**a**) and P2b (**b**) before the phantom study.

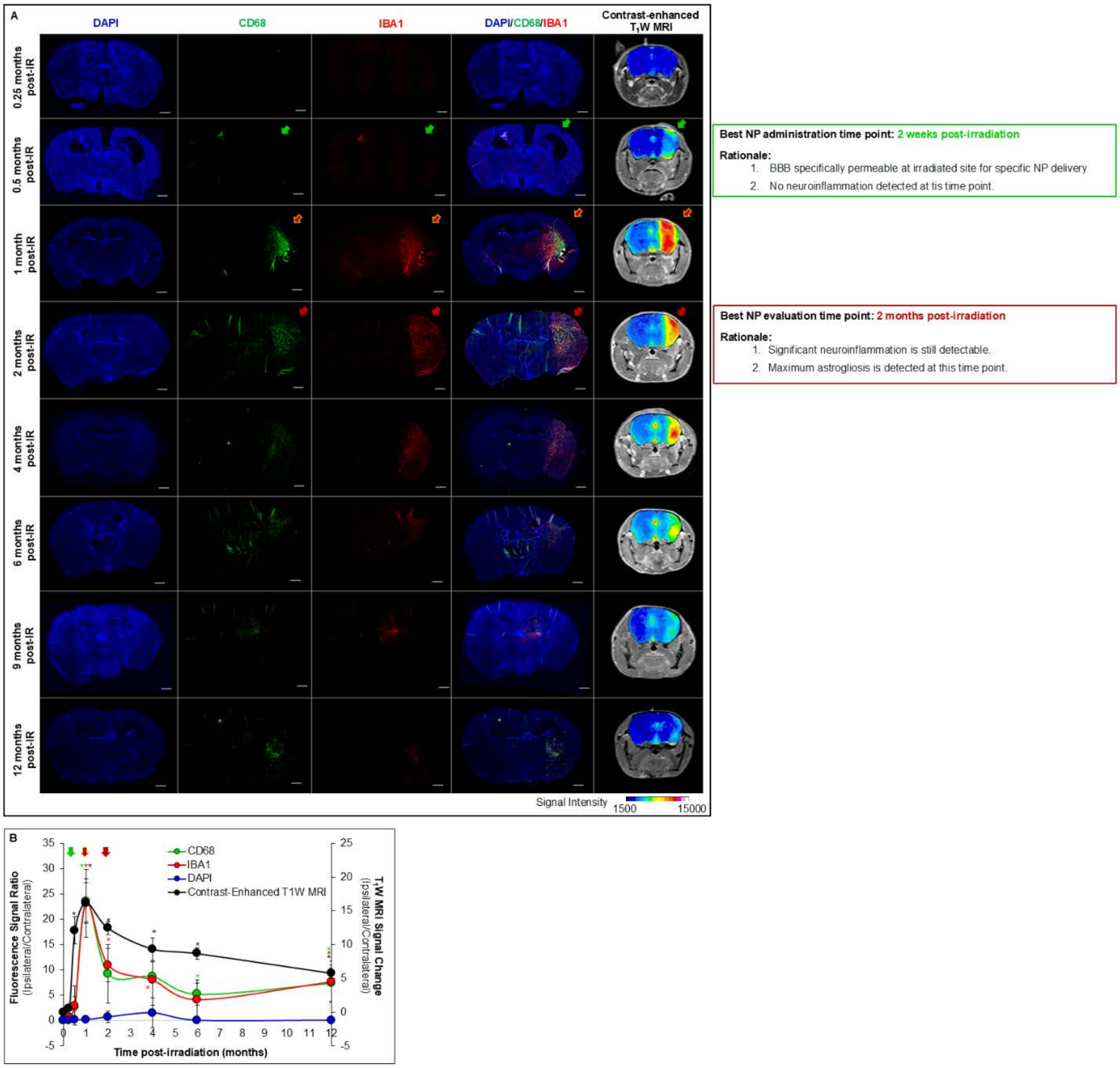

Supplemental Figure S10: Determination of the best nanoparticle administration and evaluation time points. A) Detection of neuroinflammation with CD68 (green) and IBA1 (red) immunohistochemistry at different time points post-irradiation in mice irradiated at 8-weeks-old and monitored for 12 months post-irradiation (2 mm scale bar). Maximum neuroinflammation was detected one month post-irradiation and corresponded with the maximum BBB permeability detected one month post-irradiation with *in vivo* contrast-enhanced T_1W MRI. **B**) Quantification of the respective fluorescence signals and the contrast-enhanced T_1W MRI signals in the irradiated

brain hemispheres normalized to the non-irradiated brain hemispheres at different time points post-irradiation ($P < 0.05$, n = 3). Maximum neuroinflammation was detected one month post-irradiation, and this corresponded with maximum BBB permeability detected one month post-irradiation with *in vivo* contrast-enhanced T_1W MRI. Reprinted with permission from Teitz M, Velarde E, Yang X, Lee S, Lecksell K, Terrillion C, Bibic A, Ngen EJ. Developing Magnetic Resonance Imaging Biomarkers of Neuroinflammation, Cognitive Impairment, and Survival Outcomes for Radiotherapy-Induced Brain Injury in a Preclinical Mouse Model. Invest Radiol. 2026 Jan 1;61(1):10-25. doi: 10.1097/RLI.0000000000001173. PMID: 40095964; PMCID: PMC12353168. Copyright 2025 Wolters Kluwer Health, LLC.

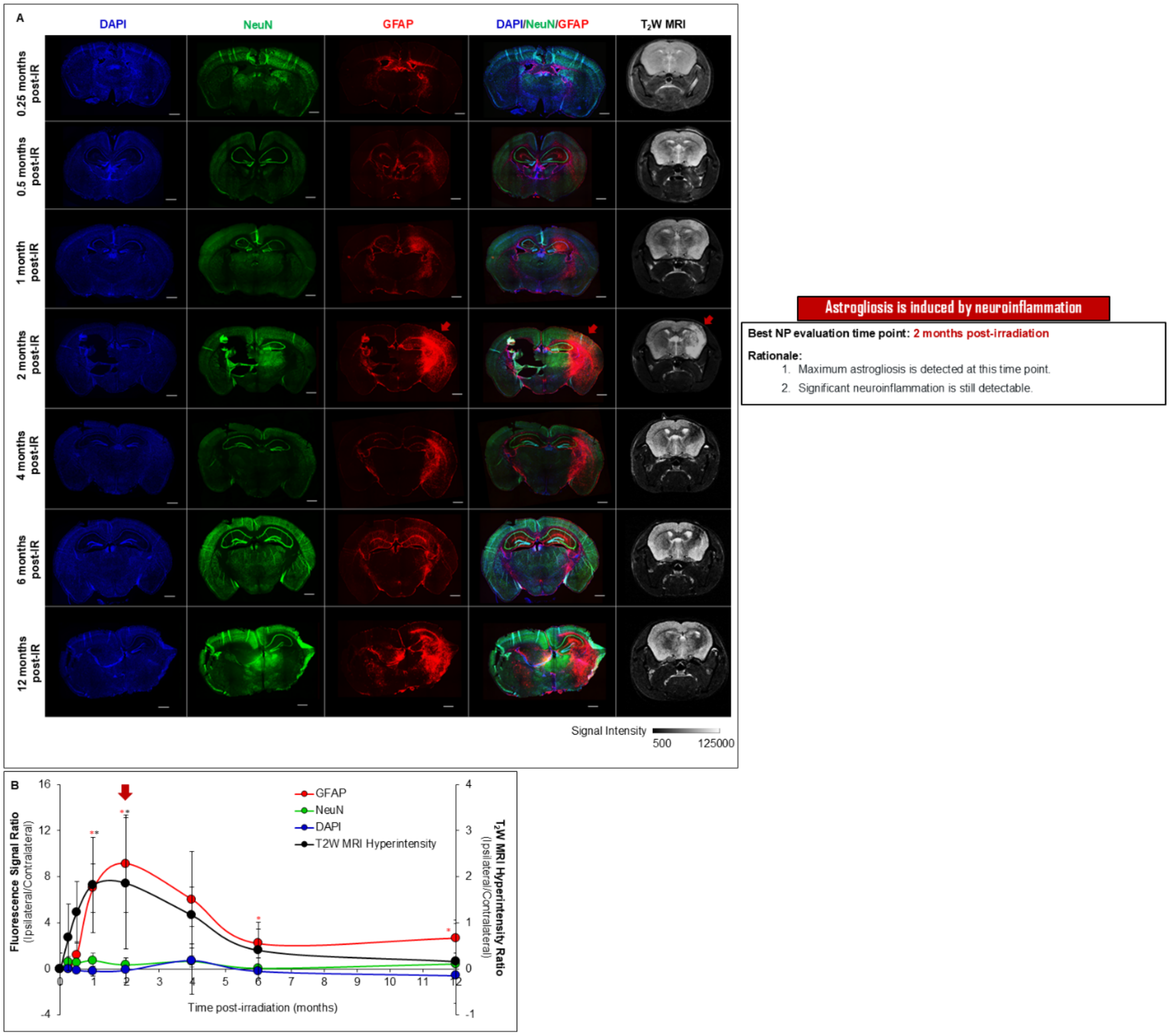

Supplemental Figure S11: Determination of the best nanoparticle evaluation time points. A) Detection of astrogliosis with GFAP (red) and neuronal loss with NeuN (green) immunohistochemistry at different time points post-irradiation in mice irradiated at 8-weeks-old and monitored for 12 months post-irradiation (2 mm scale bar). Maximum astrogliosis was detected two months post-irradiation and corresponded with the maximum *in vivo* T_2W MRI hyperintensity signal also detected two months post-irradiation. **B**) Quantification of the respective fluorescence signals and the T_2W MRI hyperintensity signals in the irradiated brain hemispheres normalized to the non-irradiated brain hemispheres at different time points post-irradiation (P <

0.05, n = 3). Maximum astrogliosis was detected at two months post-irradiation and corresponded with the maximum *in vivo* T_2W MRI hyperintensity signal also detected two-months post-irradiation. No statistically significant neuronal loss was detected within the study's 12-month post-irradiation time frame. Reprinted with permission from Teitz M, Velarde E, Yang X, Lee S, Lecksell K, Terrillion C, Bibic A, Ngen EJ. Developing Magnetic Resonance Imaging Biomarkers of Neuroinflammation, Cognitive Impairment, and Survival Outcomes for Radiotherapy-Induced Brain Injury in a Preclinical Mouse Model. Invest Radiol. 2026 Jan 1;61(1):10-25. doi: 10.1097/RLI.0000000000001173. PMID: 40095964; PMCID: PMC12353168. Copyright 2025 Wolters Kluwer Health, LLC.

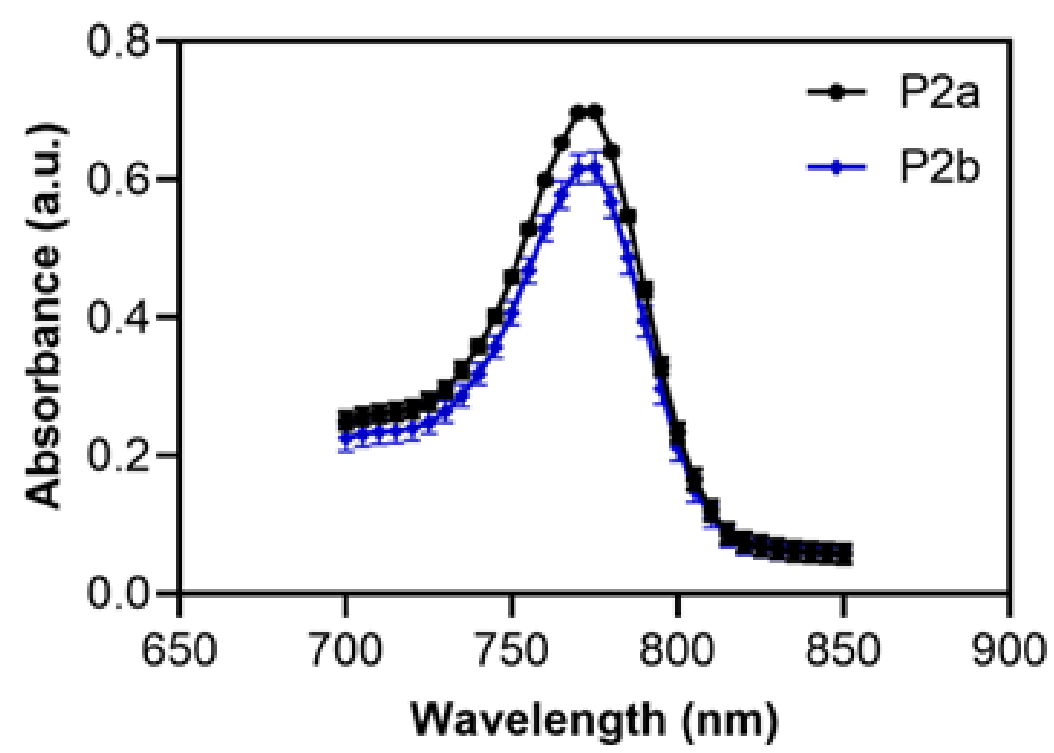

Supplemental Figure S12: Absorbance spectra of the respective polymers P2a and P2b in tetrahydrofuran (2 mg/mL) after conjugation to IRDye® 800CW.

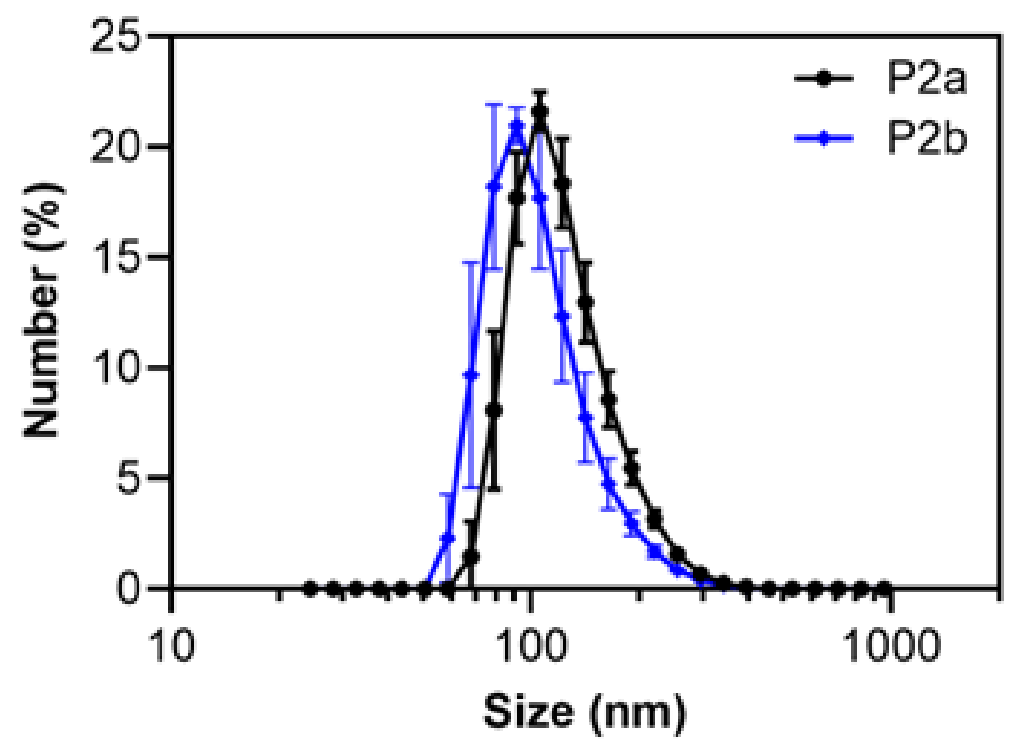

Supplemental Figure S13: Determination of the respective hydrodynamic diameters of IRDye® 800CW-conjugated and IRDye® 680RD-encapsulated polymeric nanoparticles P2a and P2b by dynamic light scattering (DLS) at pH of 7.4.

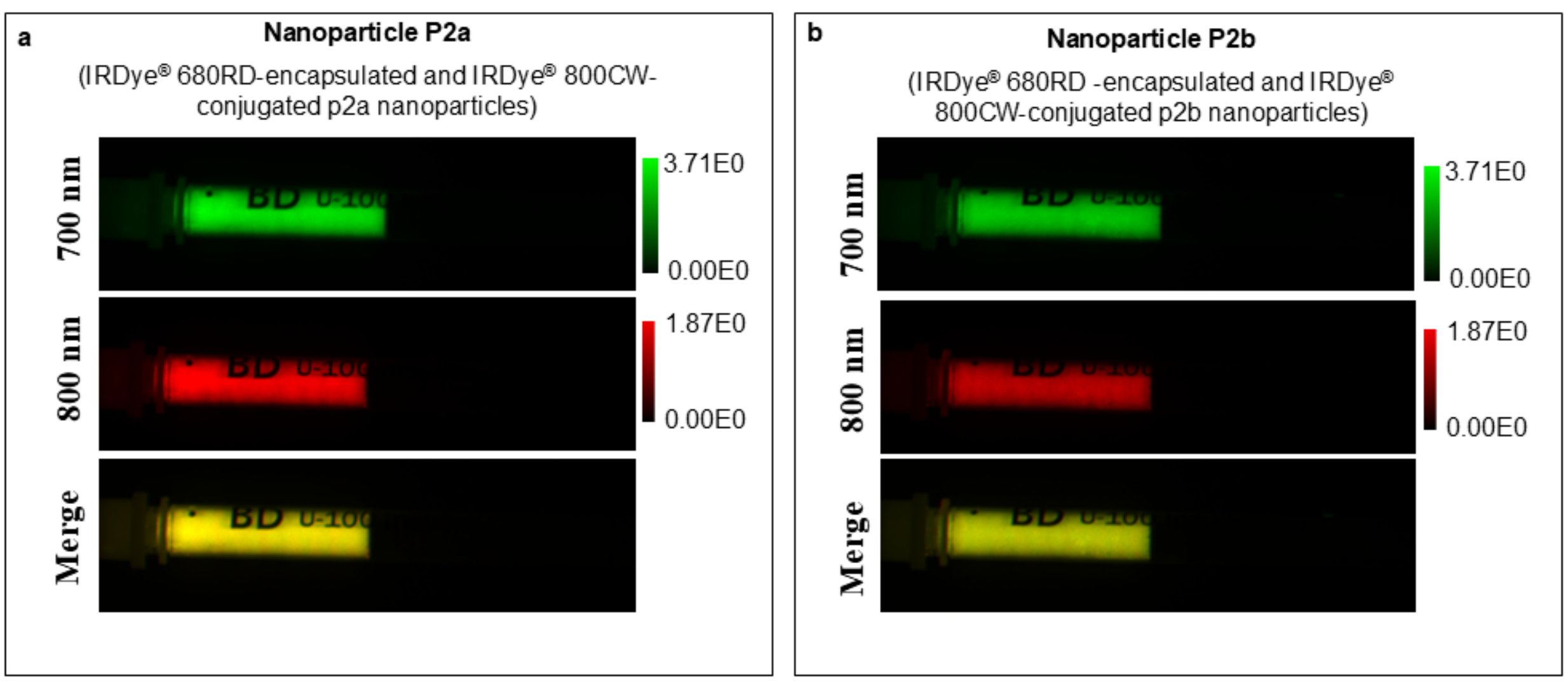

Supplemental Figure S14. Fluorescence images of IRDye® 800CW-conjugated and IRDye® 680RD-encapsulated nanoparticle P2a (**a**) and P2b (**b**) before *in vivo* administration in mice.

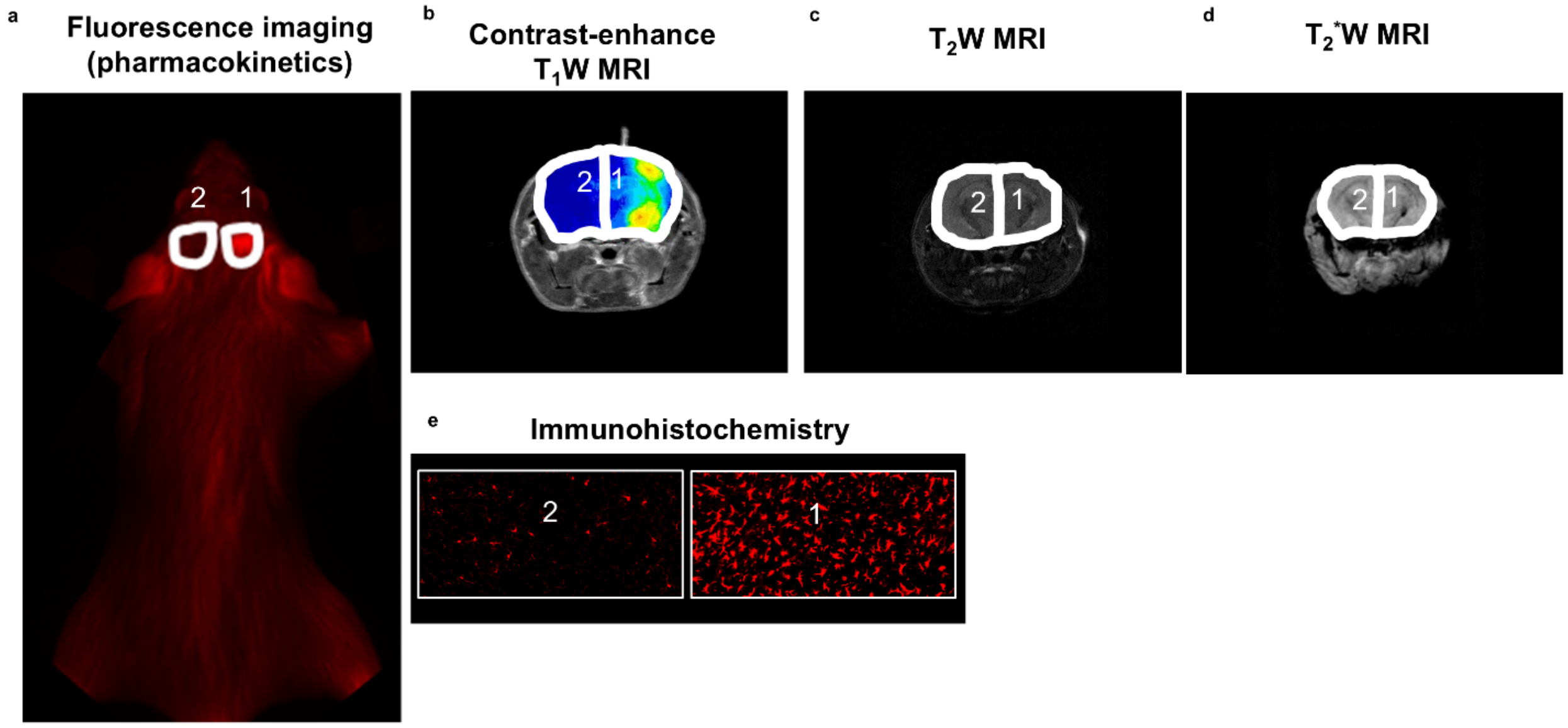

Supplemental Figure S15. Illustrations of the regions of interest (ROIs) used in the study. a) Illustration of the two ROIs used in the fluorescence imaging (pharmacokinetics) quantification. **b**) Illustration of the two ROIs used in the contrast-enhanced T_1W MRI quantification. **c**) Illustration of the two ROIs used in the respective T_2W MRI hyperintensity and hypointensity quantification. **d**) Illustration of the two ROIs used in the respective T_2^*W MRI hypointensity quantification. **e**) Illustration of the two ROIs used in the immunohistochemistry quantification.

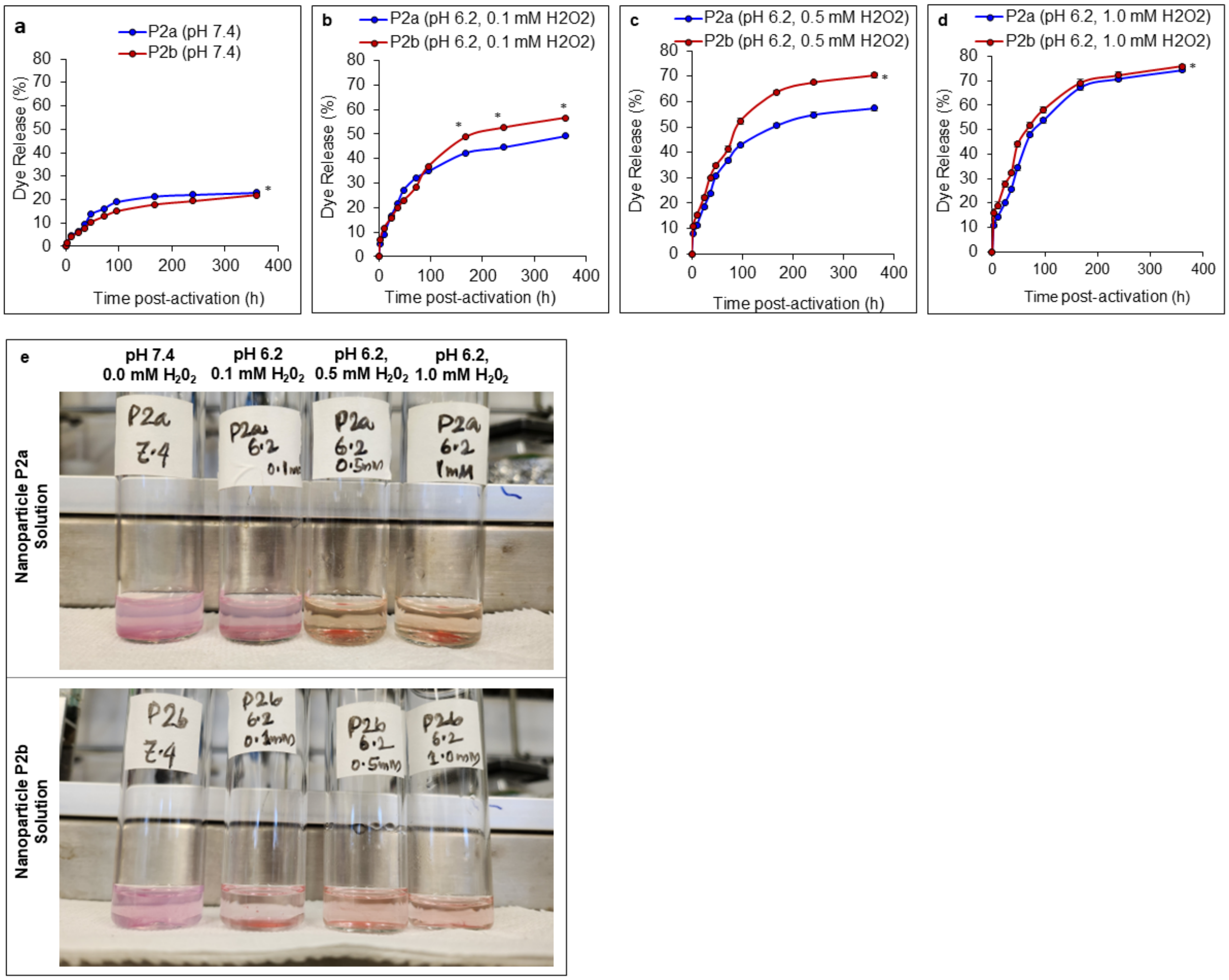

Supplemental Figure S16: Nanoparticle activation kinetics determination by fluorescence spectroscopy. Comparison of the kinetics of release of an encapsulated fluorophore (Nile Red®) from P2a compared to P2b at physiological conditions (pH 7.4 and no H_2O_2), and under varying degrees of oxidative stress (at pH 6.2 and varying concentrations of H_2O_2): **a**) Physiological conditions (pH 7.4 and no H_2O_2); **b**) 0.1 mM H_2O_2 at pH 6.2; **c**) 0.5 mM H_2O_2 at pH 6.2; **d**) 1 mM H_2O_2 at pH 6.2 ($p \leq 0.05$). Whereas the activation kinetics of nanoparticle P2a was significantly faster than that of nanoparticle P2b at physiological conditions ($P = 0.0024$, n = 3); the activation kinetics of nanoparticle P2b was significantly faster ($P < 0.05$, n = 3) than that of P2a under oxidative stress. **e**) Images of Nile Red® encapsulated nanoparticle solutions P2a and P2b under

physiological conditions (pH 7.4 and no H_2O_2) and varying degrees of oxidative stress (at pH 6.2 and varying concentrations of H_2O_2).

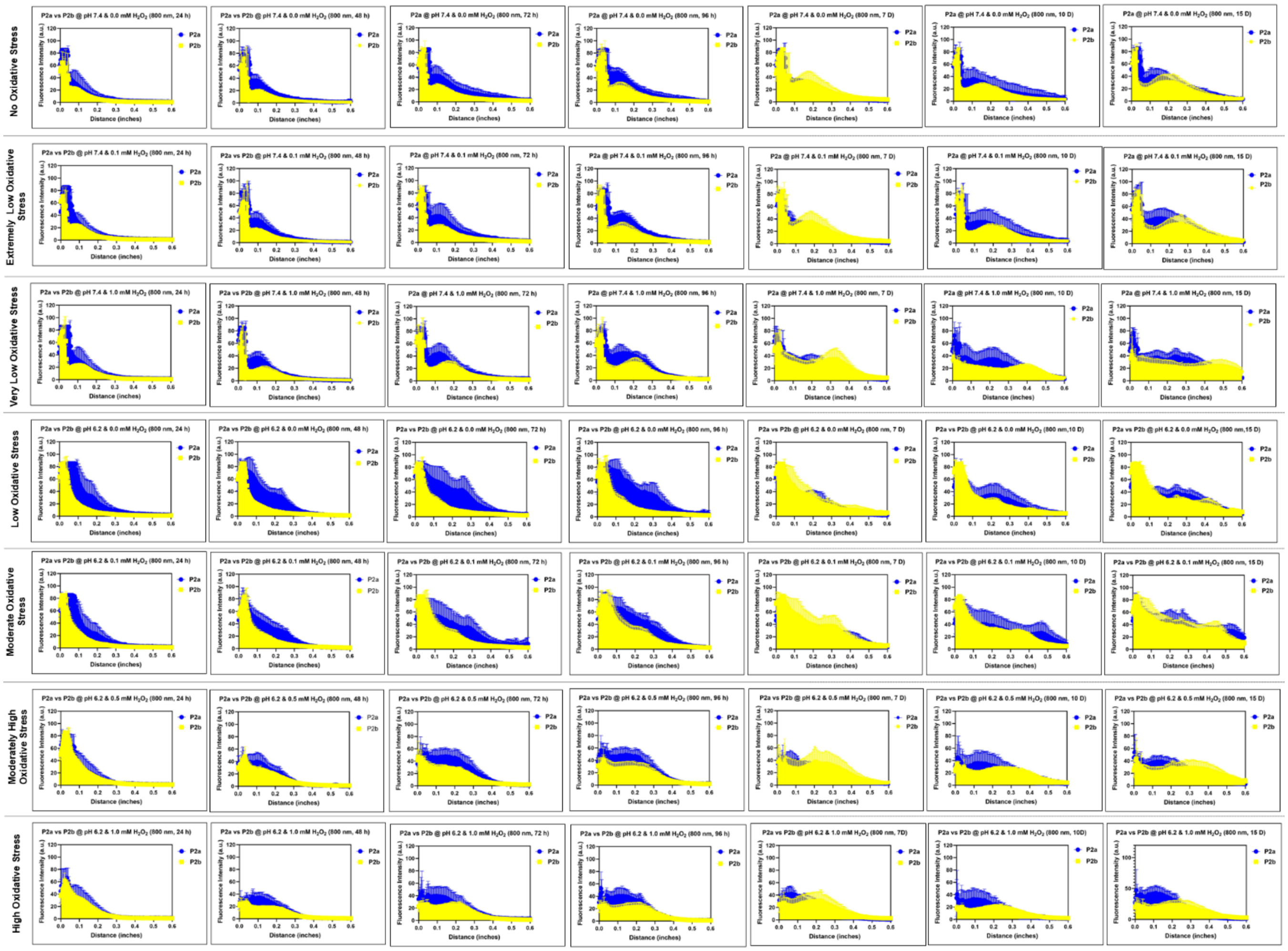

Supplemental Figure S17: Kinetics of encapsulated 800 nm fluorophore (IRDye® 800CW) release and diffusion after nanoparticle activation at different conditions of oxidative stress. Graph comparisons of the release and diffusion of the encapsulated 800 nm fluorophore (IRDye® 800CW) along the length of the agarose gel phantom tubes after nanoparticle activation at different conditions of oxidative stress. Overall, the release and diffusion kinetics of the encapsulated 800 nm fluorophore (IRDye® 800CW) released from the activated nanoparticle P2a (shown in blue)

was slower than that from nanoparticle P2b (shown in yellow). This suggested that the cargo released from nanoparticle P2a might be better retained in the tissues adjacent to the nanoparticle P2a, than that from P2b.

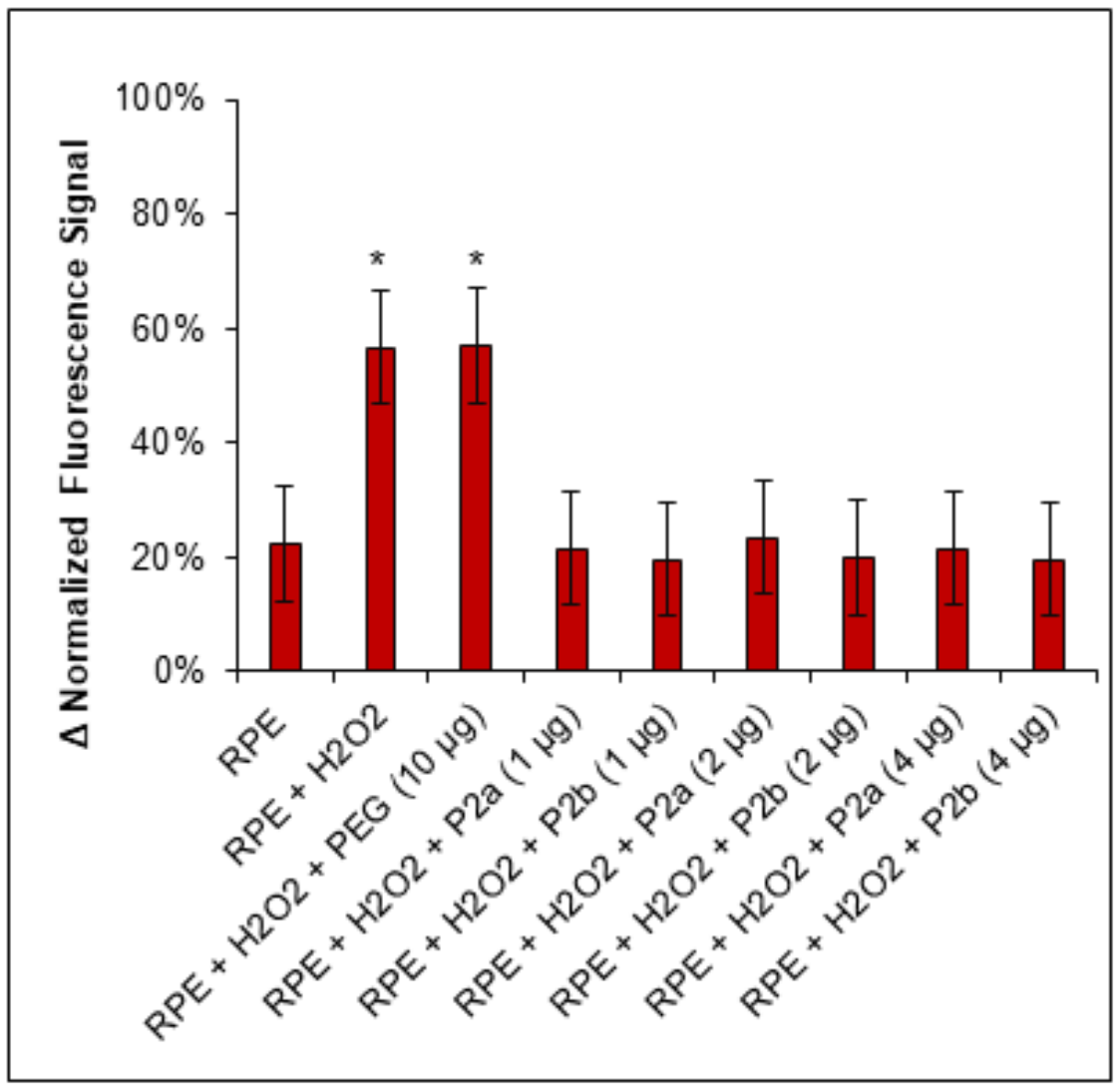

Supplemental Figure S18: Changes in the fluorescence signal intensity of RPE protein 60 minutes after exposure to oxidative stress (pH 7.0 and 10 mM H_2O_2) and its protection in the presence of nanoparticles P2a and P2b, respectively. Fluorescent RPE protection from degradation under oxidative stress was obtained with as little as 1 µg of nanoparticles P2a and P2b, respectively. 10 µg of PEG was ineffective in preventing the degradation of RPE under oxidative stress.

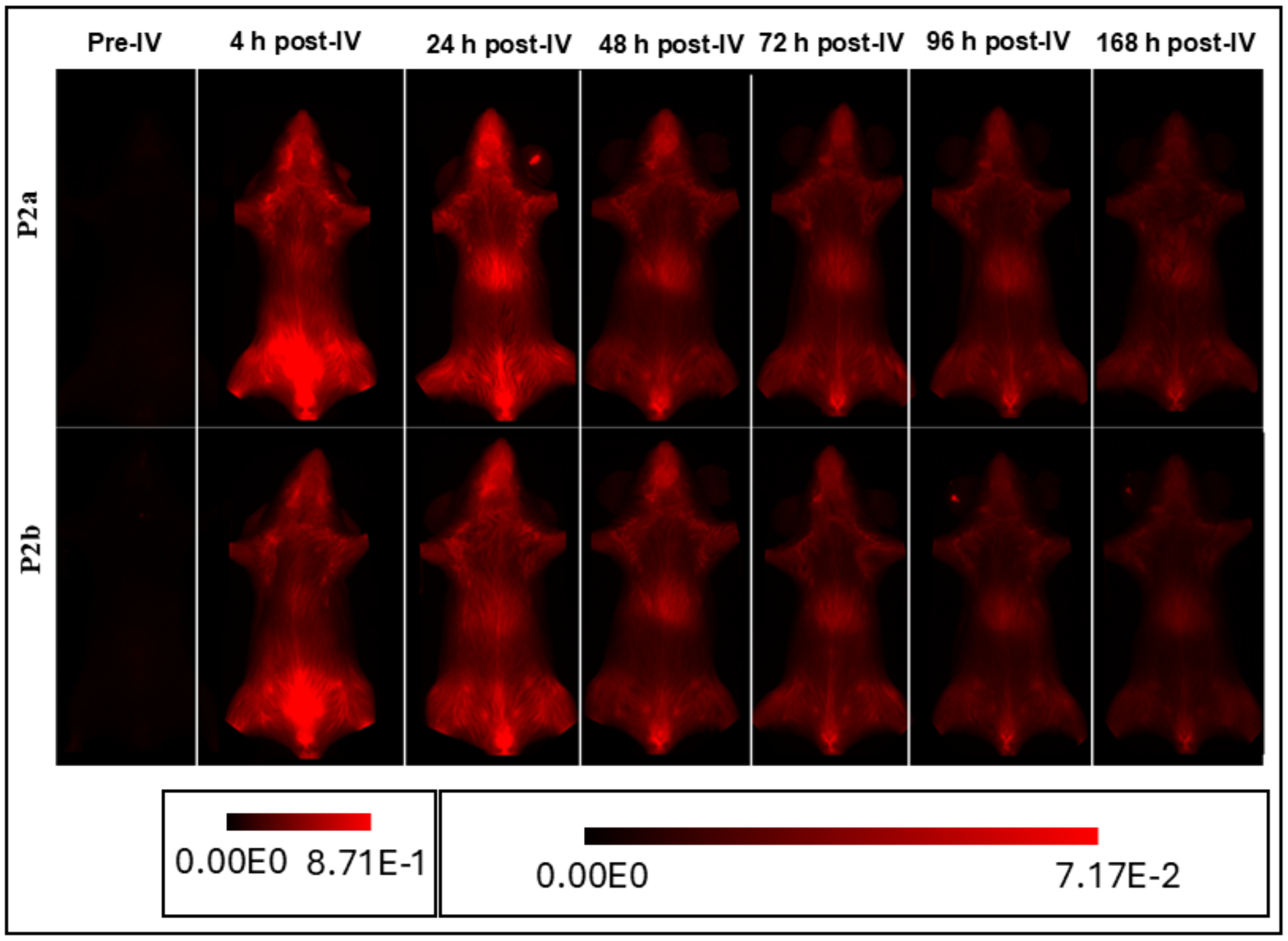

Supplemental Figure S19: Fluorescence images (820 nm) of nanoparticle accumulation in the liver after intravenous administration (0.8 mg/Kg).

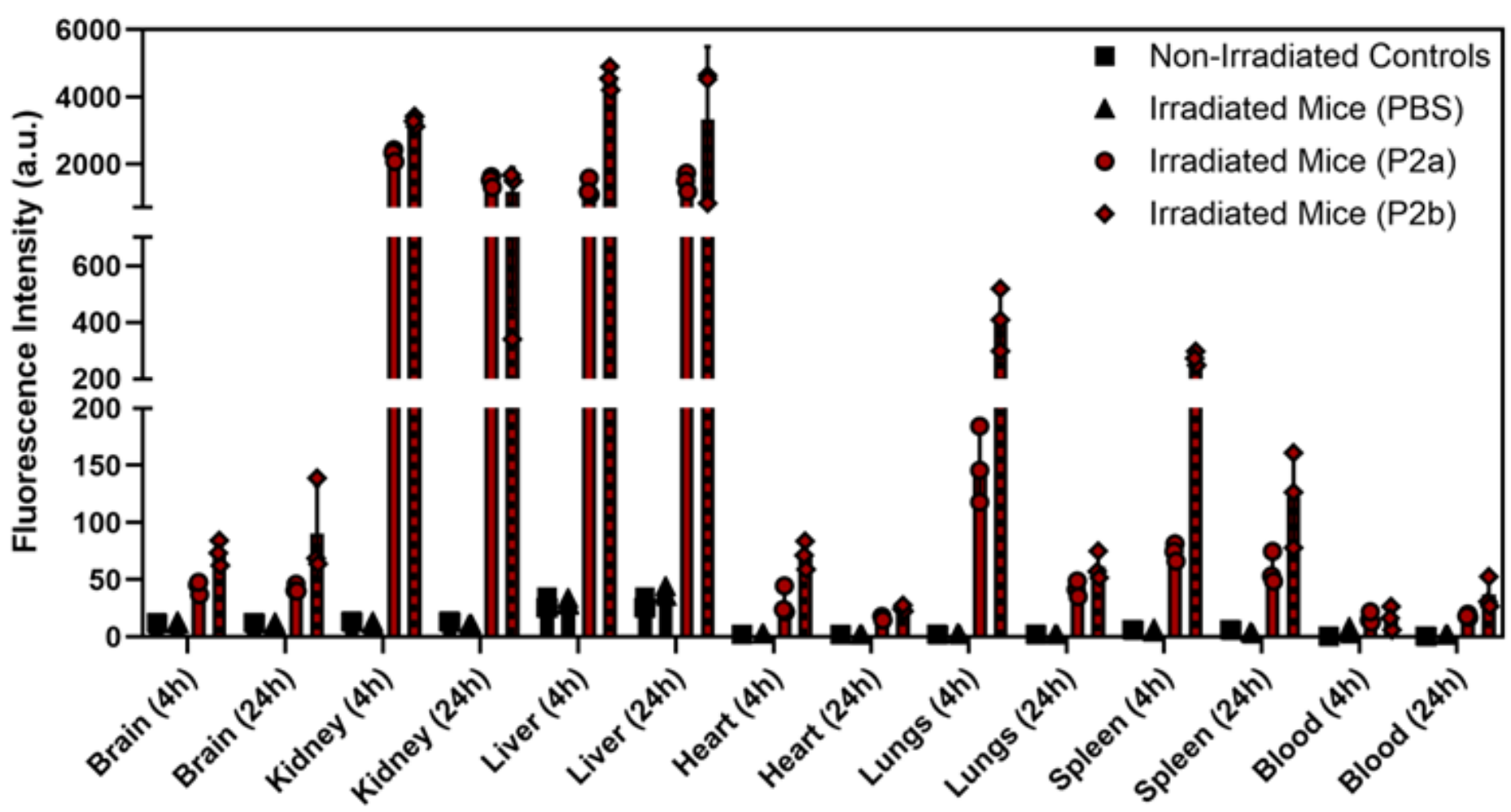

Supplemental Figure S20: Fluorescence imaging of nanoparticle biodistribution in irradiated mice. Quantification of the 820 nm fluorescence signal of the respective nanoparticles P2a and P2b in the different organs of mice after intravenous administration (0.8 mg/Kg). This

showed comparable fluorescence signals from organs such as the brain, liver and kidney at both 4 h and 24 h after intravenous nanoparticle administration.

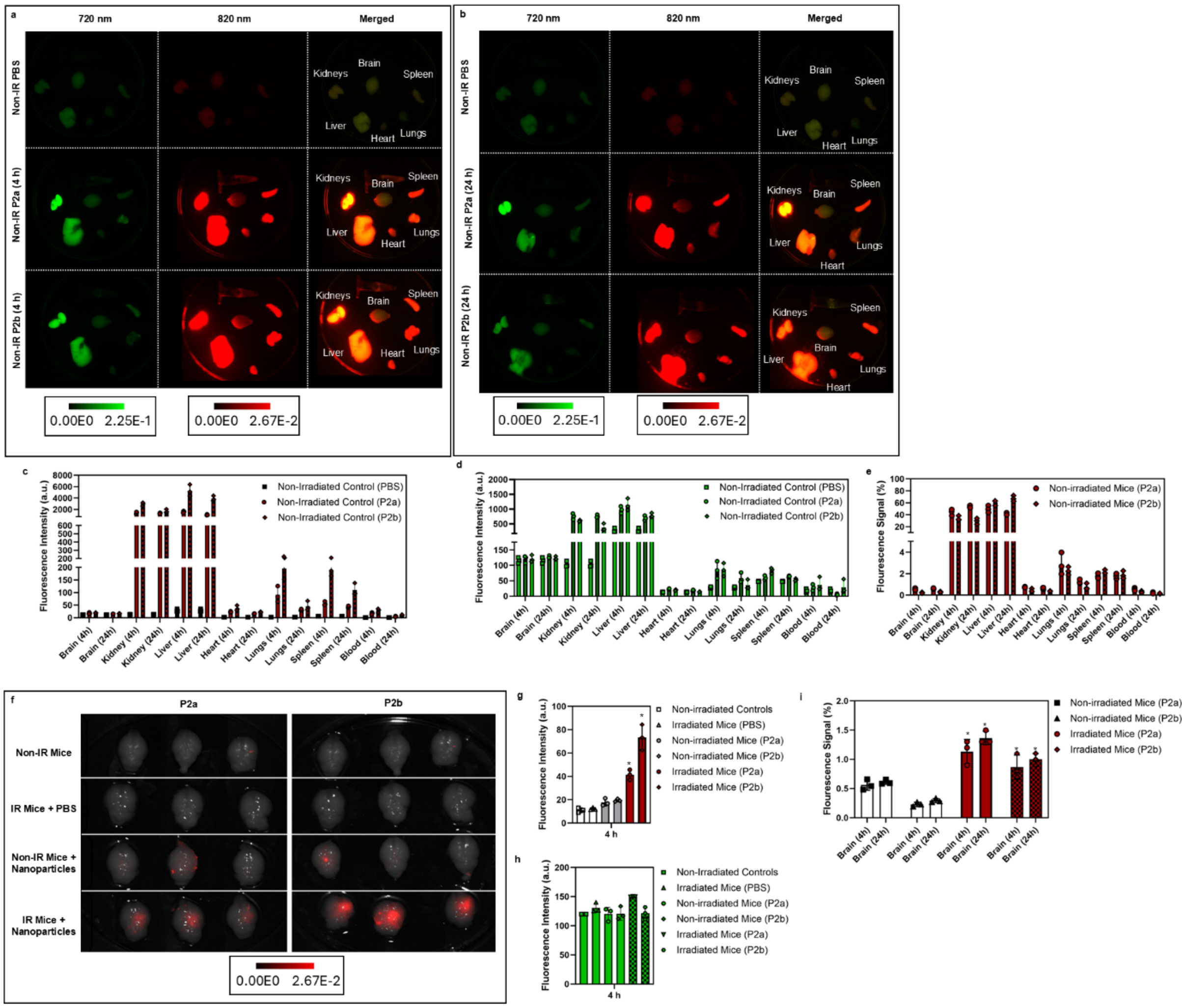

Supplemental Figure S21: Fluorescence imaging of nanoparticle biodistribution in non-irradiated mice. Fluorescence imaging of nanoparticle biodistribution in non-irradiated mice at 4 h (**a**) and 24 h (**b**) after intravenous nanoparticle administration (0.8 mg/Kg). This showed high nanoparticle uptake in the liver, kidneys, lungs, and spleen, but not in the brain. Quantification of the fluorescence signals of nanoparticle biodistribution in non-irradiated mice at 4 h and 24 h after intravenous nanoparticle administration: **c**) using the 820 nm fluorescence signal and **d**) using the

720 nm fluorescence signal. The 720 nm fluorescence images could not be used to effectively quantify the *in vivo* release of the encapsulated IRDye® 680RD dye from the activated nanoparticles, due to the high inherent background from the tissues at this wavelength **e**) Quantification of the percentage 820 nm fluorescence signals of nanoparticle biodistribution in non-irradiated mice at 4 h and 24 h after intravenous nanoparticle administration. This showed high nanoparticle uptake in the liver, kidneys, lungs, and spleen, but not in the brain. **f**) Comparison of the 820 nm fluorescence images of the nanoparticle biodistribution in the brains of non-irradiated and irradiated mice at 4 h after intravenous nanoparticle administration. Higher nanoparticle delivery was detected in the irradiated mouse brains than in the brains of non-irradiated control mice that received the nanoparticles. Quantification of the fluorescence signals of nanoparticle biodistribution in non-irradiated and irradiated mouse brains at 4 h and 24 h after intravenous nanoparticle administration: **g**) using the 820 nm fluorescence signal and **h**) using the 720 nm fluorescence signal. A higher 820 nm nanoparticle signal was detected in the irradiated mouse brains than in the brains of non-irradiated control mice that received the nanoparticles. The 720 nm fluorescence images could not be used to effectively quantify the *in vivo* release of the encapsulated fluorophore from the activated nanoparticles, due to the high inherent background from the tissues at this wavelength. **i**) Percentage quantification of the 820 nm fluorescence signals of nanoparticle biodistribution in non-irradiated and irradiated mouse brains at 4 h after intravenous nanoparticle administration. An ~ 3-fold higher nanoparticle delivery was detected in the irradiated mouse brain lesions than in the brains of non-irradiated control mice that received the nanoparticles.

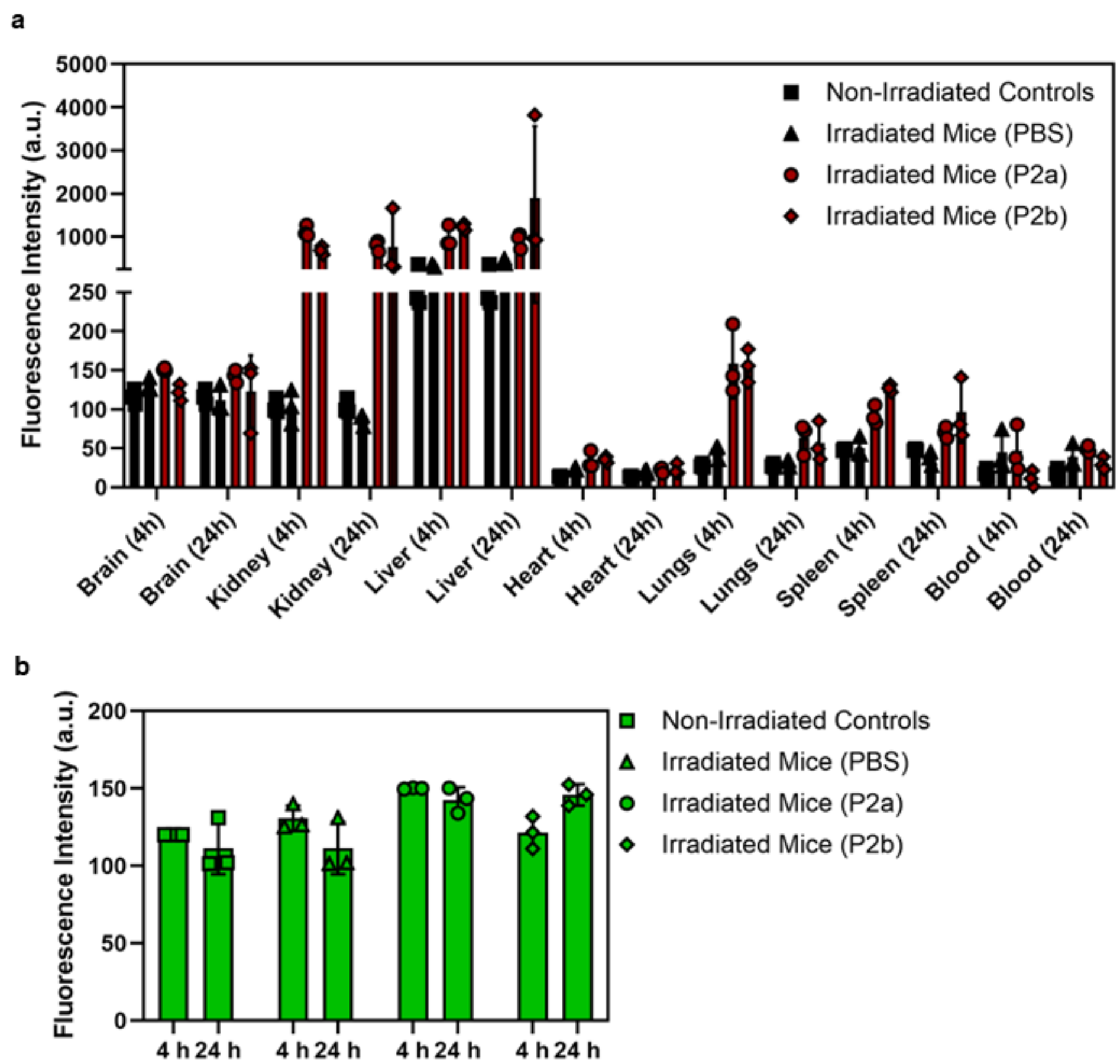

Supplemental Figure S22: Quantification of the 720 nm fluorescence signal of nanoparticle biodistribution in irradiated mice. a) Quantification of the 720 nm fluorescence signals of nanoparticle biodistribution in the organs of irradiated mice at 4 h and 24 h after intravenous nanoparticle administration. **b**) Quantification of the 720 nm fluorescence signals of nanoparticle biodistribution in the irradiated brains of mice at 4 h and 24 h after intravenous nanoparticle administration. The 720 nm fluorescence signal could not be used to effectively quantify the *in vivo* release of the encapsulated IRDye® 680RD dye from the activated nanoparticles, due to the high inherent background from the tissues at this wavelength.

Supplemental Digital Content 3

Table S1 2
Table S2 2
Table S3 3
Table S4 3
Table S5 4
Table S6 4
Table S7 4
Table S8 5
Table S9 5
Table S10 5
Table S11 6
Table S12 6
Table S13 6
Table S14 7
Table S15 7
Table S16 7
Table S17 8

Table S1: Statistics of the detection of nanoparticle P2a in the irradiated brain lesions by fluorescence imaging (820 nm)		
**Comparison (0 h versus time post-IV)**	**Statistical significance (*P*-value), sample size (n)**	**Statistical test used in the GraphPad Prism Software**
0 h versus 4 h post-IV	Yes, ($P = 0.0010$, n = 5)	Student's t-test, (Paired, two-tailed)
0 h versus 24 h post-IV	Yes, ($P < 0.0001$, n = 5)	Student's t-test, (Paired, two-tailed)
0 h versus 48 h post-IV	Yes, ($P < 0.0001$, n = 5)	Student's t-test, (Paired, two-tailed)
0 h versus 72 h post-IV	Yes, ($P < 0.0001$, n = 5)	Student's t-test, (Paired, two-tailed)
0 h versus 96 h post-IV	Yes, ($P < 0.0001$, n = 5)	Student's t-test, (Paired, two-tailed)
0 h versus 168 h post-IV	Yes, ($P < 0.0001$, n = 5)	Student's t-test, (Paired, two-tailed)

Table S2: Statistics of the detection of nanoparticle P2b in the irradiated brain lesions by fluorescence imaging (820 nm)		
**Comparison (0 h versus time post-IV)**	**Statistical significance (*P*-value), sample size (n)**	**Statistical test used in the GraphPad Prism Software**
0 h versus 4 h post-IV	Yes, ($P < 0.0001$, n = 5)	Student's t-test, (Paired, two-tailed)
0 h versus 24 h post-IV	Yes, ($P < 0.0001$, n = 5)	Student's t-test, (Paired, two-tailed)
0 h versus 48 h post-IV	Yes, ($P < 0.0001$, n = 5)	Student's t-test, (Paired, two-tailed)
0 h versus 72 h post-IV	Yes, ($P < 0.0001$, n = 5)	Student's t-test, (Paired, two-tailed)
0 h versus 96 h post-IV	Yes, ($P < 0.0001$, n = 5)	Student's t-test, (Paired, two-tailed)
0 h versus 168 h post-IV	Yes, ($P < 0.0001$, n = 5)	Student's t-test, (Paired, two-tailed)

Table S3: Statistics of the detection of nanoparticles P2a versus P2b in the irradiated brain lesions by fluorescence imaging (820 nm)		
**Comparison (P2a versus P2b)**	**Statistical significance (*P*-value), sample size (n)**	**Statistical test used in the GraphPad Prism Software**
4 h post-IV	No, ($P = 0.3737$, n = 5)	Student's t-test, (Paired, two-tailed)
24 h post-IV	No, ($P = 0.0722$, n = 5)	Student's t-test, (Paired, two-tailed)
48 h post-IV	No, ($P = 0.7176$, n = 5)	Student's t-test, (Paired, two-tailed)
72 h post-IV	Yes, ($P = 0.0002$, n = 5)	Student's t-test, (Paired, two-tailed)
96 h post-IV	No, ($P = 0.1957$, n = 5)	Student's t-test, (Paired, two-tailed)
168 h post-IV	No, ($P = 0.0106$, n = 5)	Student's t-test, (Paired, two-tailed)

Table S4: Statistics of blood-brain barrier (BBB) permeability changes in the control group versus the irradiated groups		
**Comparison (Control versus irradiated groups)**	**Statistical significance (*P*-value), sample size (n)**	**Statistical test used in the GraphPad Prism Software**
Control versus irradiated (PBS-treated)	Yes, ($P < 0.000001$, n = 3)	Student's t-test, (Unpaired, two-tailed)
Control versus irradiated (P2a-treated)	Yes, ($P = 0.000045$, n = 3)	Student's t-test, (Unpaired, two-tailed)
Control versus irradiated (P2b-treated)	Yes, ($P < 0.000397$, n = 3)	Student's t-test, (Unpaired, two-tailed)

Table S5: Statistics of blood-brain barrier (BBB) permeability changes in the irradiated (PBS-treated) group versus the other irradiated groups		
**Comparison (Irradiated (PBS-treated) versus other irradiated groups)**	**Statistical significance (*P*-value), sample size (n)**	**Statistical test used in the GraphPad Prism Software**
Irradiated (PBS-treated) versus irradiated (P2a-treated)	No, ($P = 0.2406$, n = 3)	Student's t-test, (Unpaired, two-tailed)
Irradiated (PBS-treated) versus irradiated (P2b-treated)	Yes, ($P = 0.0198$, n = 3)	Student's t-test, (Unpaired, two-tailed)

Table S6: Statistics of the T_2W MRI hyperintensity signal of the control group versus the irradiated groups		
**Comparison (Control versus irradiated groups)**	**Statistical significance (*P*-value), sample size (n)**	**Statistical test used in the GraphPad Prism Software**
Control versus irradiated (PBS-treated)	Yes, ($P = 0.0045$, n = 3)	Student's t-test, (Unpaired, two-tailed)
Control versus irradiated (P2a-treated)	No, ($P = 0.1763$, n = 3)	Student's t-test, (Unpaired, two-tailed)
Control versus irradiated (P2b-treated)	No, ($P < 0.2414$, n = 3)	Student's t-test, (Unpaired, two-tailed)

Table S7: Statistics of the T_2W MRI hyperintensity signal of the irradiated (PBS-treated) group versus the other irradiated groups		
**Comparison (Irradiated (PBS-treated) versus other irradiated groups)**	**Statistical significance (*P*-value), sample size (n)**	**Statistical test used in the GraphPad Prism Software**
Irradiated (PBS-treated) versus irradiated (P2a-treated)	Yes, ($P = 0.0071$, n = 3)	Student's t-test, (Unpaired, two-tailed)
Irradiated (PBS-treated) versus irradiated (P2b-treated)	Yes, ($P = 0.0130$, n = 3)	Student's t-test, (Unpaired, two-tailed)

**Table S8: Statistics of the T_2W MRI hypointensity signal of the control group versus the irradiated groups**		
**Comparison (Control versus irradiated groups)**	**Statistical significance (*P*-value), sample size (n)**	**Statistical test used in the GraphPad Prism Software**
Control versus irradiated (PBS-treated)	No, ($P = 0.1332$, n = 3)	Student's t-test, (Unpaired, two-tailed)
Control versus irradiated (P2a-treated)	No, ($P = 0.1001$, n = 3)	Student's t-test, (Unpaired, two-tailed)
Control versus irradiated (P2b-treated)	No, ($P < 0.1216$, n = 3)	Student's t-test, (Unpaired, two-tailed)

**Table S9: Statistics of the T_2W MRI hypointensity signal of the irradiated (PBS-treated) group versus the other irradiated groups**		
**Comparison (Irradiated (PBS-treated) versus other irradiated groups)**	**Statistical significance (*P*-value), sample size (n)**	**Statistical test used in the GraphPad Prism Software**
Irradiated (PBS-treated) versus irradiated (P2a-treated)	No, ($P = 0.1236$, n = 3)	Student's t-test, (Unpaired, two-tailed)
Irradiated (PBS-treated) versus irradiated (P2b-treated)	No, ($P = 0.1474$, n = 3)	Student's t-test, (Unpaired, two-tailed)

**Table S10: Statistics of the T_2^*W MRI hypointensity signal of the control group versus the irradiated groups**		
**Comparison (Control versus irradiated groups)**	**Statistical significance (*P*-value), sample size (n)**	**Statistical test used in the GraphPad Prism Software**
Control versus irradiated (PBS-treated)	Yes, ($P = 0.0085$, n = 3)	Student's t-test, (Unpaired, two-tailed)
Control versus irradiated (P2a-treated)	Yes, ($P = 0.00013$, n = 3)	Student's t-test, (Unpaired, two-tailed)
Control versus irradiated (P2b-treated)	Yes, ($P = 0.0268$, n = 3)	Student's t-test, (Unpaired, two-tailed)

**Table S11: Statistics of the T_2^*W MRI hypointensity signal of the irradiated (PBS-treated) group versus the other irradiated groups**		
**Comparison (Irradiated (PBS-treated) versus other irradiated groups)**	**Statistical significance (*P*-value), sample size (n)**	**Statistical test used in the GraphPad Prism Software**
Irradiated (PBS-treated) versus irradiated (P2a-treated)	No, (*P* = 0.3936, n = 3)	Student's t-test, (Unpaired, two-tailed)
Irradiated (PBS-treated) versus irradiated (P2b-treated)	No, (*P* = 0.7626, n = 3)	Student's t-test, (Unpaired, two-tailed)

**Table S12: CD68 immunohistochemistry statistics of the control group versus the irradiated groups**		
**Comparison (Control versus irradiated groups)**	**Statistical significance (*P*-value), sample size (n)**	**Statistical test used in the GraphPad Prism Software**
Control versus irradiated (PBS-treated)	Yes, (*P* = 0.0001, n = 3)	Student's t-test, (Unpaired, two-tailed)
Control versus irradiated (P2a-treated)	Yes, (*P* = 0.0001, n = 3)	Student's t-test, (Unpaired, two-tailed)
Control versus irradiated (P2b-treated)	Yes, (*P* = 0.039, n = 3)	Student's t-test, (Unpaired, two-tailed)

**Table S13: CD68 immunohistochemistry statistics of the irradiated (PBS-treated) group versus the other irradiated groups**		
**Comparison (Irradiated (PBS-treated) versus other irradiated groups)**	**Statistical significance (*P*-value), sample size (n)**	**Statistical test used in the GraphPad Prism Software**
Irradiated (PBS-treated) versus irradiated (P2a-treated)	No, (*P* = 0.3013, n = 3)	Student's t-test, (Unpaired, two-tailed)
Irradiated (PBS-treated) versus irradiated (P2b-treated)	Yes, (*P* = 0.0004, n = 3)	Student's t-test, (Unpaired, two-tailed)

**Table S14: IBA1 immunohistochemistry statistics of the control group versus the irradiated groups**		
**Comparison (Control versus irradiated groups)**	**Statistical significance (*P*-value), sample size (n)**	**Statistical test used in the GraphPad Prism Software**
Control versus irradiated (PBS-treated)	Yes, (*P* = 0.0003, n = 3)	Student's t-test, (Unpaired, two-tailed)
Control versus irradiated (P2a-treated)	Yes, (*P* = 0.0014, n = 3)	Student's t-test, (Unpaired, two-tailed)
Control versus irradiated (P2b-treated)	No, (*P* = 0.0912, n = 3)	Student's t-test, (Unpaired, two-tailed)

**Table S15: IBA1 immunohistochemistry statistics of the irradiated (PBS-treated) groups versus the other irradiated groups**		
**Comparison (Irradiated (PBS-treated) versus other irradiated groups)**	**Statistical significance (*P*-value), sample size (n)**	**Statistical test used in the GraphPad Prism Software**
Irradiated (PBS-treated) versus irradiated (P2a-treated)	Yes, (*P* = 0.0018, n = 3)	Student's t-test, (Unpaired, two-tailed)
Irradiated (PBS-treated) versus irradiated (P2b-treated)	Yes, (*P* = 0.0002, n = 3)	Student's t-test, (Unpaired, two-tailed)

**Table S16: GFAP immunohistochemistry statistics of the control group versus the irradiated groups**		
**Comparison (Control versus irradiated groups)**	**Statistical significance (*P*-value), sample size (n)**	**Statistical test used in the GraphPad Prism Software**
Control versus irradiated (PBS-treated)	Yes, (*P* = 0.0011, n = 3)	Student's t-test, (Unpaired, two-tailed)
Control versus irradiated (P2a-treated)	Yes, (*P* = 0.0009, n = 3)	Student's t-test, (Unpaired, two-tailed)
Control versus irradiated (P2b-treated)	Yes, (*P* = 0.0003, n = 3)	Student's t-test, (Unpaired, two-tailed)

**Table S17: GFAP immunohistochemistry statistics of the irradiated (PBS-treated) group versus the other irradiated groups**		
**Comparison (Irradiated (PBS-treated) versus other irradiated groups)**	**Statistical significance (*P*-value), sample size (n)**	**Statistical test used in the GraphPad Prism Software**
Irradiated (PBS-treated) versus irradiated (P2a-treated)	Yes, (*P* = 0.0030, n = 3)	Student's t-test, (Unpaired, two-tailed)
Irradiated (PBS-treated) versus irradiated (P2b-treated)	Yes, (*P* = 0.0019, n = 3)	Student's t-test, (Unpaired, two-tailed)
